# A proteomic roadmap of SaOS-2 osteoblast-to-osteocyte-like differentiation

**DOI:** 10.1016/j.bonr.2026.101961

**Published:** 2026-10-23

**Authors:** Britta Eggers, Sascha Roocke, Svitlana Rozanova, Karin Schork, Maximilian Schütte, Mira Struwe, Paul Zandner, Maximilian Hausherr, Greta Domroese, Morris Gellisch, Martin Eisenacher, Jacqueline Heinen-Weiler, Carsten Theiss, Annette Richter-Unruh, Katalin Barkovits, Katrin Marcus

**Affiliations:** a Medizinisches Proteom-Center, Medical Faculty, Ruhr University Bochum, Bochum, 44801, Germany; b Medical Proteome Analysis, Center for Protein Diagnostics (PRODI), Ruhr University Bochum, Bochum, 44801, Germany; c Core Unit for Bioinformatics (CUBiMed.RUB), Medical Faculty, Ruhr University Bochum, Bochum, 44801, Germany; d BioInfra.Prot, de.NBI - German Network for Bioinformatics Infrastructure, Medical Faculty, Ruhr University Bochum, Bochum, Germany; e Department of Anatomy and Molecular Embryology, Institute of Anatomy, Medical Faculty, Ruhr University Bochum; f Center for Medical Education, Ruhr University Bochum, Bochum, 44801, Germany; g Medical Imaging Center (MIC), Electron Microscopy Medical Analysis - Core Facility (EMMA^CF^), Medical Faculty, Ruhr University Bochum, 44801, Bochum, Germany; h Department of Cytology, Institute of Anatomy, Ruhr University Bochum, Bochum, 44801, Germany; i Clinic for Children and Adolescents, Pediatric Endocrinology, St. Josef-Hospital, Bochum, D-44791, Germany; j Hormonzentrum für Kinder und Jugendlich in ÜBAG MVZ Eberhard Partner Dortmund, Germany

**Keywords:** SaOS-2, Osteoblast, Osteocyte, Osteocyte-like cells, Differentiation, Proteomics, Extracellular matrix

## Abstract

Mineralized bone tissue is essential for skeletal integrity, mechanical stability, and mineral homeostasis. Osteocytes, the most abundant bone cell type, play central roles in bone remodeling and endocrine regulation but remain difficult to investigate because of their embedded localization within the mineralized matrix. Consequently, the molecular mechanisms underlying osteoblast-to-osteocyte differentiation remain incompletely understood.

Here, we performed a comprehensive quantitative proteomic analysis of the widely used human SaOS-2 cell line during osteoblast-to-osteocyte-like differentiation. More than 5000 proteins were quantified, providing a comprehensive proteomic roadmap of this differentiation process. Proteomic profiling demonstrated extensive molecular remodeling accompanied by extracellular matrix reorganization and dynamic changes in collagen abundance. Although differentiated SaOS-2 cells retained characteristics of late osteoblasts and did not fully recapitulate the phenotype of mature osteocytes, they acquired a distinct osteocyte-like proteomic signature. Furthermore, we identified and orthogonally validated several candidate protein markers associated with the osteoblast and osteocyte-like states.

Together, our study provides the first comprehensive protein-level characterization of SaOS-2 osteoblast-to-osteocyte-like differentiation. Beyond improving the molecular characterization of this widely used model, it provides a valuable resource for future studies investigating bone biology, extracellular matrix remodeling, and skeletal disease.

## Highlights

• Comprehensive proteomic roadmap of SaOS-2 osteoblast-to-osteocyte-like differentiation
• Dynamic extracellular matrix and collagen remodeling accompanies differentiation
• Novel candidate protein markers identified for osteoblast and osteocyte-like cell states

## Graphical abstract

**Figure:**
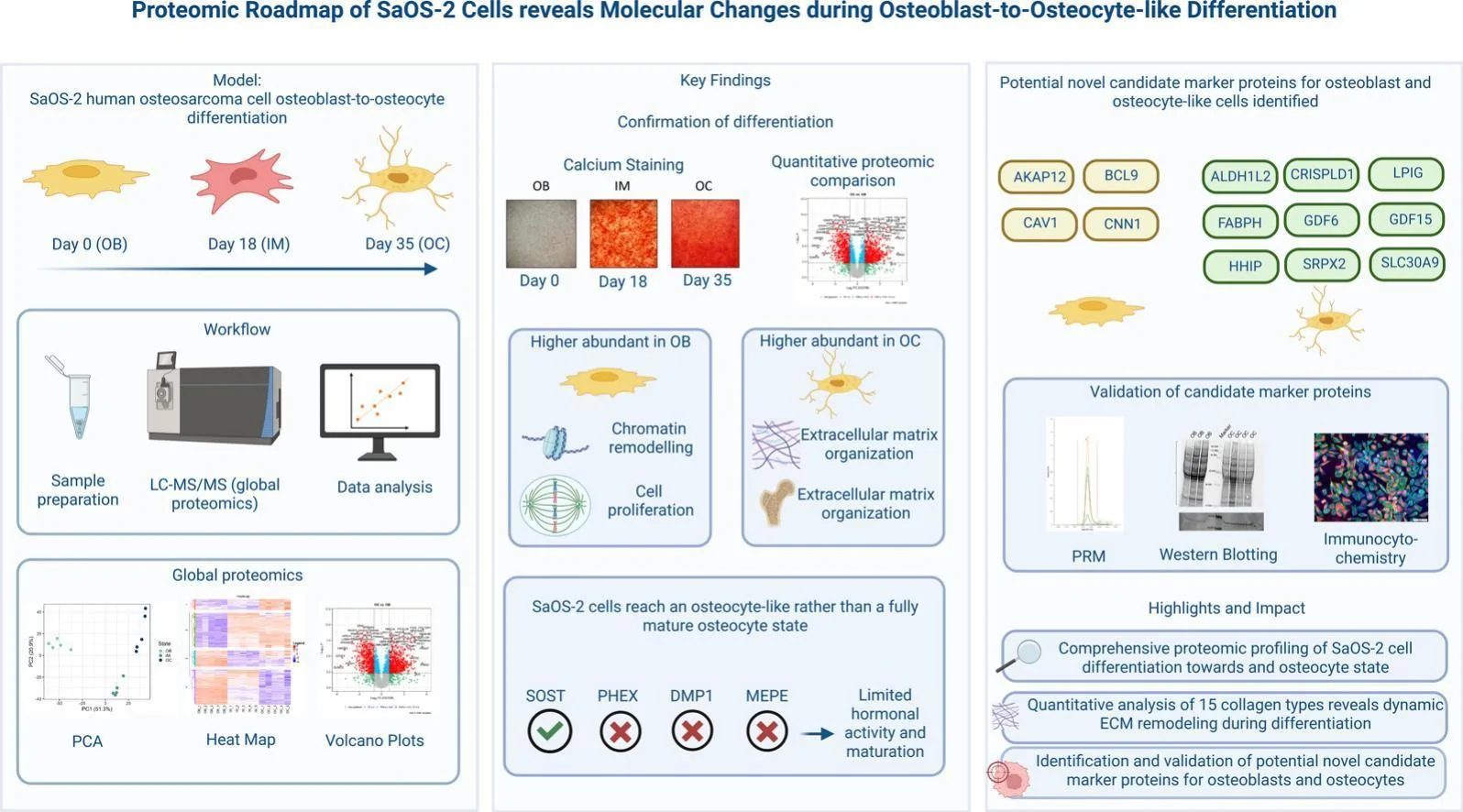

## 1 Introduction

Bone is a highly dynamic organ that continuously adapts to mechanical demands while fulfilling essential structural, metabolic, and endocrine functions. Maintaining skeletal integrity depends on the coordinated activity of osteoblasts (OB), osteocytes (OC), and osteoclasts, with OC now recognized as the central regulators of bone homeostasis (Florencio-Silva et al., 2015).

OB synthesize and organize the extracellular bone matrix and secrete numerous structural, enzymatic, and regulatory proteins that contribute to bone formation and systemic regulation of haematopoiesis, mineral homeostasis, and energy metabolism (Donsante et al., 2021). At the end of their life cycle, OB either undergo apoptosis, become bone-lining cells, or become embedded within the mineralized matrix, where they differentiate into metabolically active OC (Delgado-Calle and Bellido, 2022). This transition is accompanied by profound morphological and functional remodeling, including reduced proliferation, extensive extracellular matrix deposition, formation of the lacuno-canalicular network, and acquisition of mechanosensory and endocrine functions. Understanding the molecular programs orchestrating this differentiation process is essential for deciphering bone development, homeostasis, and disease.

Although OC account for approximately 90–95% of all bone cells and orchestrate skeletal remodeling, the molecular events underlying OB-to-OC differentiation remain incompletely understood. Mature OC are embedded within the mineralized bone matrix, making them difficult to isolate and analyze. Consequently, they remain among the least well-characterized cell types in vertebrates (Robling and Bonewald, 2020).

Several in vitro models have been established to investigate OB biology [5, 6]. However, suitable human models that recapitulate OB-to-OC differentiation remain scarce because primary OC are difficult to isolate and exhibit only limited proliferative capacity.

SaOS-2 cells currently represent the only widely used human cell model that recapitulates OB-to-OC-like differentiation in vitro. During differentiation, they form a mineralized extracellular matrix and express markers characteristic of OC-like cells after 28–35 days in differentiation medium (Prideaux et al., 2014).

Only few studies have investigated this differentiation process at the molecular level, and these have relied exclusively on transcriptomic approaches [7]. Although transcriptomics has provided valuable insights into OB-to-OC differentiation, mRNA abundance reflects protein abundance and activity only incompletely. Because proteins execute cellular function, comprehensive proteomic characterization is essential for understanding the molecular mechanisms underlying this transition.

To address this gap, we performed a comprehensive quantitative proteomic characterization of human SaOS-2 differentiation to establish a protein-level atlas of OB-to-OC differentiation. Beyond defining global proteomic remodeling during differentiation, we systematically evaluated established differentiation markers, identified previously unrecognized cell-state-associated proteins, and propose novel candidate protein markers for OB- and OC-like cells. Together, our study provides the first comprehensive proteomics roadmap of OB-to-OC differentiation and establishes a molecular resource for future studies of bone biology and skeletal disease.

## 2 Material and methods

### 2.1 Cell culture

SaOS-2 cells were obtained from DSMZ (German Collection of Microorganisms and Cell Cultures GmbH). Cells were cultured in αMEM supplemented with 10% fetal bovine serum (FBS; PAN Biotech) and 1% penicillin/streptomycin (Gibco, Thermo Fisher Scientific) at 37 °C in a humidified atmosphere containing 5% CO₂ to maintain an osteoblast (OB)-like phenotype. The culture medium was replaced two to three times per week, and cells were passaged using trypsin upon reaching confluence. For osteocyte (OC)-like cell differentiation, the culture medium was supplemented with 1.8 mM KH₂PO₄ and 50 μg/ml ascorbic acid (both Merck, Sigma-Aldrich), while the FBS concentration was reduced to 5%. Cells were differentiated for 18 days to achieve an intermediate (IM) cell type and up to 35 days to reach the OC-like cell state. To ensure comparable starting conditions, cells were counted using a Neubauer counting chamber and seeded at a density of 2 × 10^4^ cells/cm^2^ before differentiation was initiated. Differentiation was confirmed by Alizarin Red staining.

### 2.2 Calcium stainin

The degree of osteogenic differentiation was assessed by staining extracellular calcium with Alizarin Red S (Merck Millipore). Cells were seeded into 6-well plates (2 × 10^4^ cells/cm^2^), washed once with PBS, and fixed with 4% formaldehyde (AppliChem GmbH) for 10 min at 4 °C. After two additional washing steps with PBS, cells were incubated with 1 ml Alizarin Red S solution for 10 min at room temperature (RT). Excess dye was removed by repeated washing with PBS until the washing solution remained clear.

For quantitative analysis, stained cultures were incubated with 1 ml of 10% cetylpyridinium chloride (Merck, Sigma-Aldrich) for 45 min at 37 °C under gentle shaking. The extracted dye was transferred to a cuvette, and absorbance was measured at 470 nm using a spectrophotometer. Three independent biological replicates were analyzed for each differentiation stage (OB, IM, and OC-like cells).

### 2.3 Immunocytochemistry

30,000 cells seeded in 24 wells containing glass cover slips. Undifferentiated OBs and fully differentiated OC were washed with sterile PBS and fixated in 4% paraformaldehyde for 30 min at RT. For immunocytochemistry, cells were blocked with 5% BSA in sterile PBS for one hour. Incubation of primary antibodies was carried out over night at 4 °C (see supplementary Table S1). Subsequently cover slips were washed three times with sterile PBS. Incubation with secondary antibodies was carried out at RT for one hour followed by three washing steps (see supplementary Table S1). Cover slips were mounted on ultrafrost glas slides with mounting media containing DAPI (VECTASHIELD Antifade Mounting Medium with DAPI, Biozol, Hamburg Germany). An Olympus VS120 BX61VS virtual slide microscope and OlyVIA software (version 2.9) were utilized for fluorescence microscopy.

### 2.4 Standard cell lysis and protein extraction

For mass spectrometric analysis, SaOS-2 cells in OB, IM, and OC-like states were analyzed. Five biological replicates were harvested per condition by cell scraping and lysed according to a previously described protocol (Stratmann et al., 2022) with minor modifications.

Cells were washed twice with ice-cold PBS and scraped into PBS supplemented with protease inhibitors (cOmplete Protease Inhibitor Cocktail; Roche). After transfer to Protein LoBind tubes, samples were centrifuged at 400 ×*g* for 5 min at 4 °C, and the supernatant was discarded. The cell pellet was weighed, and 8 M urea buffer was added at 1.4 times the pellet volume. Samples were homogenized using a stainless-steel pestle and sonicated for 8 min in a VialTweeter ultrasonic homogenizer (UP200St, Hielscher Ultrasonics GmbH) using 30 s pulses separated by 15 s pauses at 60% power. After centrifugation at 14,000 ×*g* for 15 min at 10 °C, the supernatant was collected, and the protein concentration was determined using a Bradford assay.

### 2.5 Western blotting

Western blotting was performed with an independent cohort of OB (n = 3) and OC-like cells (n = 4) cultured and processed as described above. Quantitative Western blotting was performed according to previously published protocol (Barkovits et al., 2021; Eggers et al., 2021).

Protein concentration was determined using the Bradford assay, and 20 μg of total protein per sample was separated on a 12% Bis-Tris gel (50 V for 15 min followed by 180 V for 1 h) using MOPS running buffer (50 mM MES, 50 mM Tris, 2 mM EDTA). Quantitative protein detection was performed using the Smart Protein Layers (SPL) Western Blotting system (NH DyeAGNOSTICS GmbH, Halle, Germany), according to the manufacturer's instructions. Semi-dry Western Blotting was performed as described in (Barkovits et al., 2021; Eggers et al., 2021). Membrane blocking was performed for 1 h at RT. Primary antibodies (see supplementary Table S1) were incubated overnight at 8 °C. After three washing steps with TBS, incubation of secondary antibodies was carried out for 1 h at RT (see supplementary Table S1) Protein abundance was quantified using the accompanying SPL LabImage software, and results are reported as normalized protein volumes (SPL normalized volume).

### 2.6 Spectral library generation

An in-depth spectral library was generated for subsequent data-independent acquisition (DIA) analysis. Proteins from five biological replicates of each differentiation state were combined to generate pooled OB, IM, and OC-like samples.

The pooled samples were fractionated by SDS-PAGE using a 12% Bis-Tris gel run at 50 V for 15 min followed by 180 V for 1 h. Gels were stained with Coomassie and destained overnight (Supplementary Fig. S23). Each of the three lanes was cut into ten fractions, which were subjected to in-gel digestion as previously described in (Plum et al., 2020).

In parallel, pooled samples were also processed by standard in-solution digestion.

### 2.7 In solution digestion

Tryptic in solution digestion was carried out as described in (Stratmann et al., 2022). In brief 10 μg of cell lysate was added to 50 mM ammonium bicarbonate (AmBic). Samples were reduced with 5 mM dithiothreitol (DTT) for 20 min at 56 °C, 350 rpm and alkylated with 15 mM iodoacetamide (IAA) for 15 min in the dark at RT. For digestion, trypsin (Promega Trypsin Gold) was added at a ratio of 1:50 (trypsin to protein) and incubated over night at 37 °C. Digestion was stopped by acidification with TFA. Finally, samples were evaporated in a vacuum centrifuge and replenished to a final volume of 40 μl with 0.1% TFA.

### 2.8 LC-MS/MS analyses

Peptide concentration was determined by amino acid analysis [44], and 200 ng of peptides were injected for LC-MS/MS analysis. Samples from the differentiation experiment were analyzed on an Ultimate 3000 RSLC nano-LC system (Thermo Fisher Scientific, Bremen, Germany) coupled online to an Orbitrap Fusion Lumos mass spectrometer (Thermo Fisher Scientific, Bremen, Germany). Data-independent acquisition (DIA) was performed for quantitative proteome analysis.

Samples used for spectral library generation were analyzed on a Vanquish Neo UHPLC system (Thermo Fisher Scientific, Bremen, Germany) coupled online to an Orbitrap Fusion Lumos mass spectrometer in data-dependent acquisition (DDA) mode. In addition, parallel reaction monitoring (PRM) was performed to validate selected on/off protein candidates.

Detailed information on chromatographic conditions, mass spectrometric settings, and the peptides included in the PRM assay is provided in the Supplementary Methods. The mass spectrometry proteomics data have been deposited with the ProteomeXchange Consortium via the PRIDE partner repository (Perez-Riverol et al., 2025) under the dataset identifier PXD073069.

### 2.9 Raw data processing

DIA data were processed using Spectronaut (version 19.5.241126.62635; Biognosys, Schlieren, Switzerland). A spectral library was generated using the Pulsar Search Engine as previously described (Eggers et al., 2021).

DIA data were analyzed using the default BGS settings, with trypsin specified as the digestion enzyme, carbamidomethylation of cysteine as fixed modification, and oxidation of methionine as variable modification. The proteotypicity filter for quantification was set to protein group-specific, and normalisation was disabled.

### 2.10 Parallel reaction monitoring raw data analysis

Parallel reaction monitoring (PRM) data were analyzed as previously described by Wulf et al. (2022). Proteins of interest were selected based on the results of the global proteomic analysis (Supplementary Methods Table 1). Peptide-level output was processed using the same workflow as the protein-group data. Peptides assigned to the selected proteins were evaluated based on fold change, retention time, and charge state.

PRM data were further assessed using Skyline software (version 24.1.0.199) (Pino et al., 2020). Group comparisons were based on MS2 peak areas, and Benjamini-Hochberg-adjusted p-values <0.05, were considered as statistically significant.

### 2.11 Statistical data analysis

Statistical data analysis of the DIA data performed using R (version 4.2.2) (R Core Team, 2021), RStudio (version 2023.06.0), Microsoft Excel (Microsoft 365, Version 2310) and Perseus (version 2.0.10.0) (Tyanova et al., 2016).

Non-normalized protein intensity values were log2-transformed and normalized by locally weighted scatterplot smoothing (LOESS) using the ‘ProtStatsWF’ R-package (version 1.0.0) (see supplementary Table S2). Proteins detected in fewer than three out of five biological replicates in a given group were excluded from statistical analysis. Remaining missing values were imputed from the normal distribution.

Differences among the three differentiation states were assessed by analysis of variance (ANOVA). Proteins with a permutation-based false discovery rate–adjusted *p*-value ≤0.01 were considered significantly differentially abundant. Unsupervised hierarchical clustering was used to group proteins with similar abundance profiles across differentiation states, using Pearson correlation as the distance measure.

Pairwise comparisons between differentiation states were performed using Welch's *t*-test. Benjamini–Hochberg-adjusted p-values <0.01 were considered statistically significant.

Statistical analyses and visualizations were generated using the ProtStatsWF R package (version 1.0.0) and EnhancedVolcano (version 1.22.0).

### 2.12 Gene ontology enrichment analysis

Gene ontology (GO)-term enrichment analysis was performed with DAVID (Da Huang et al., 2009; Sherman et al., 2022). GO-term analysis was performed on the basis of biological processes (BP) and only terms with a p-value <0.05 were considered.

## 3 Results

### 3.1 Differentiation of SaOS-2 cells into OC-like cells is confirmed by progressive matrix mineralization and extensive functional proteome remodeling

As matrix mineralization is a hallmark of OC-like differentiation, calcium staining assay was performed on undifferentiated OB (day 0, 0d), fully differentiated OC (day 35, 35d), and partly differentiated SaOS-2 cells in an intermediate (IM) state (day 18, 18 d). Consistent with previous work, a progressive increase in mineralization during the course of differentiation could be verified (see supplementary Fig. 1A,B and supplementary Table S3).

To characterize the molecular processes accompanying SaOS-2 differentiation, we compared the proteomic profiles of OB, IM, and OC-like cells. ANOVA identified proteins with significant abundance changes across the three differentiation states (permutation-based FDR ≤ 0.01; Supplementary Table S4). Hierarchical clustering grouped these proteins into four clusters with distinct abundance profiles (Fig. 1). Cluster 1 was found to contain proteins being of higher abundance in OC-like cells. Confirmatively GO-term enrichment analysis on the basis of biological processes revealed the term “osteoblast differentiation” being among the Top5 terms displaying the lowest p-value. Other highly significant terms were mainly associated with vesicular structures, such as “Golgi to plasma membrane transport” and “endocytic recycling”. In contrast proteins enriched in OB cells (Cluster 4), were found to be associated with terms such as “mRNA processing”, “mRNA splicing, via spliceosome and “RNA splicing”.

These findings indicate a transition from a proliferative OB state toward a functionally specialized OC-like phenotype.

**Fig. 1:**
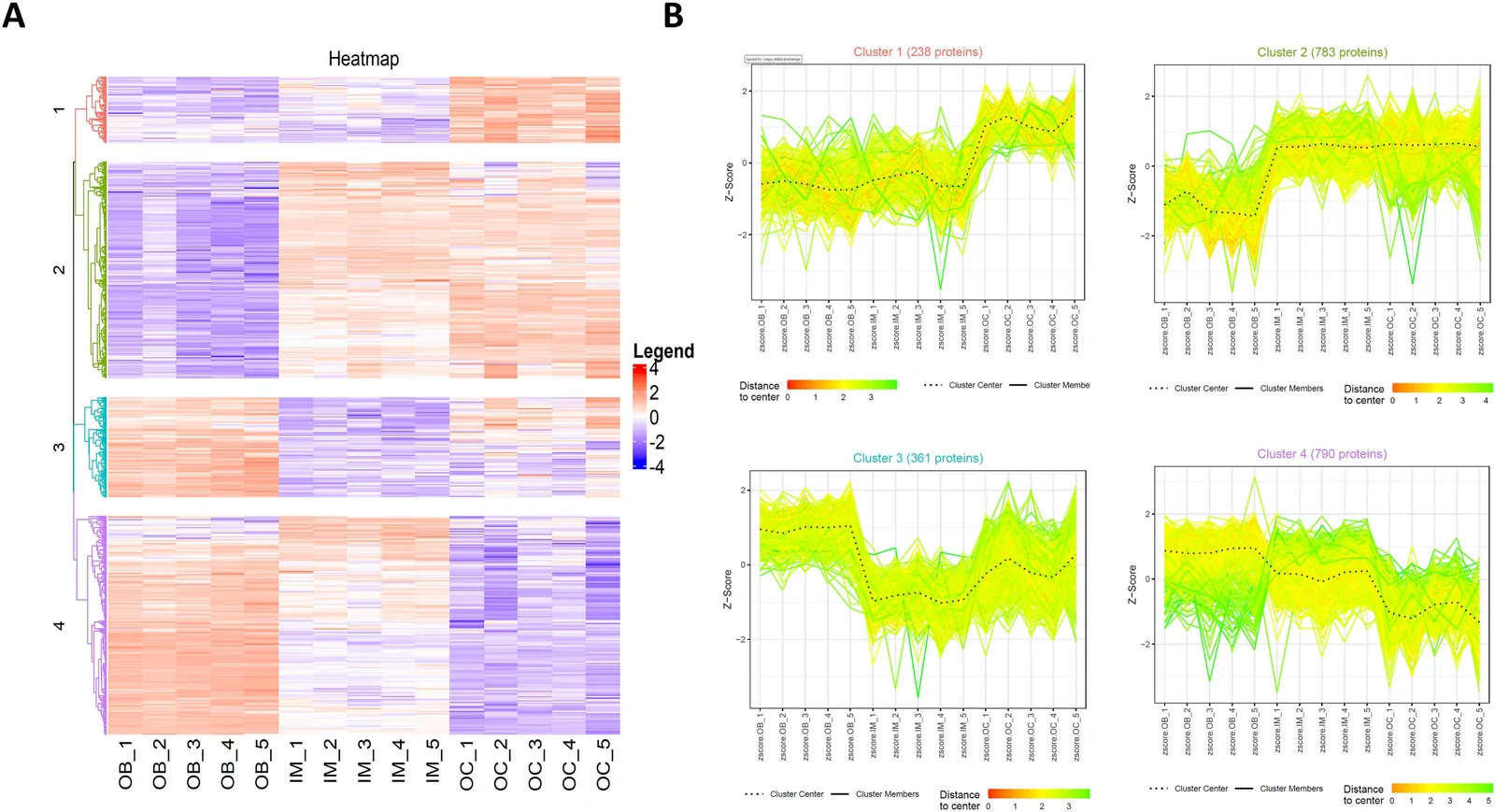
Hierarchical clustering of proteins differentially abundant during SaOS-2 differentiation. Mean protein intensities of osteoblasts (OB), intermediate cells (IM), and osteocyte-like cells (OC-like cells) were z-score normalized and visualized as a heatmap (A, ANOVA, permutation-based FDR ≤ 0.01; n = 5 per differentiation state). Hierarchical clustering identified four protein clusters with distinct abundance profiles, which are additionally shown as line plots (B).

The extensive proteomic remodeling during differentiation was also reflected in principal component analysis, which clearly separated OB, IM, and OC-like samples (Fig. 2A). Pairwise comparisons identified numerous differentially abundant proteins between the differentiation states (Welch's *t*-test, Benjamini–Hochberg-adjusted p-value ≤0.01; Supplementary Table S5). More than 30% of all quantified proteins differed between OB and OC-like cells (Fig. 2B). A similarly pronounced shift was observed between OB and IM cells (Fig. 2C). By contrast, IM and OC-like cells showed the smallest difference, with 13% of quantified proteins being differentially abundant (Fig. 2D), consistent with their greater proteomic similarity.

To define proteins that most strongly distinguished the OB and OC-like states, we next examined the 20 proteins with the largest fold changes (Table 1, Table 2). Proteins enriched in OB included craniofacial development protein 1 (CFDP1), high mobility group box 3 (HMGB3), and protein LBH (LBH), which have been linked to chromatin regulation and osteogenic processes [8–10]. LBH has been reported to suppress runt-related transcription factor 2 (RUNX2) activity and delay bone mineralization [10], consistent with its higher abundance in the less differentiated OB state. Several OB-enriched proteins, including melanoma-associated antigen 9 (MAGEA9), nuclear ubiquitous casein and cyclin-dependent kinase substrate 1 (NUCKS1), and acidic leucine-rich nuclear phosphoprotein 32 family member B (ANP32B), have also been associated with malignant cellular phenotypes [11,12], reflecting the osteosarcoma origin of SaOS-2 cells.

**Fig. 2:**
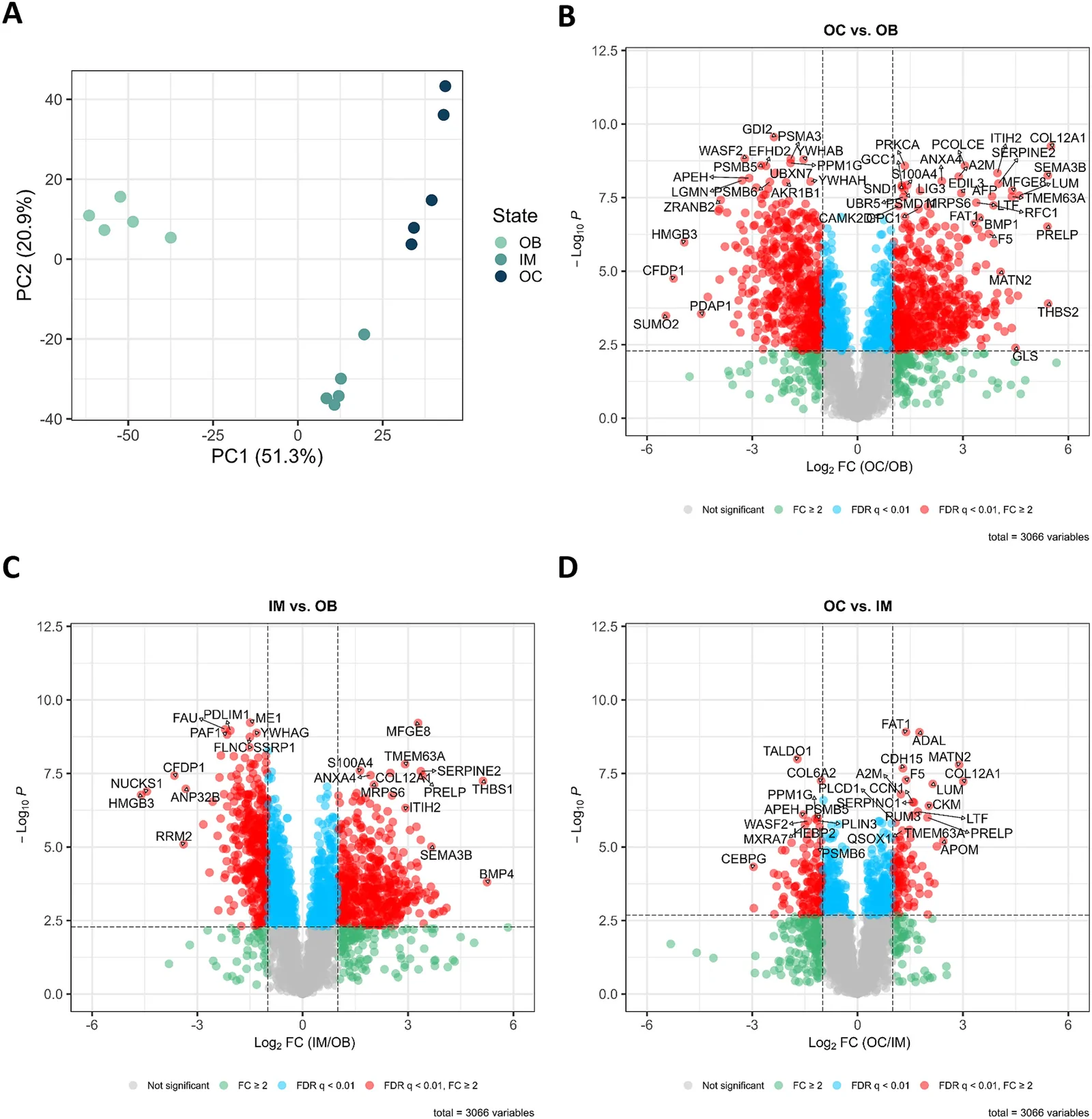
Proteomic remodeling during SaOS-2 differentiation. (A) Principal component analysis of OB, IM, and OC-like samples (n = 5 per differentiation state). (B–D) Volcano plots showing pairwise comparisons between OB and OC-like cells (B), IM and OB cells (C), and IM and OC-like cells (D). Proteins meeting the predefined significance criteria are highlighted. The numbers of differentially abundant proteins were 1088, 850, and 227, respectively.

**Table 1:** Top 20 proteins with increased abundance in OB compared with OC-like cells. Gene name and protein name are indicated as well as calculated *q*-values and fold changes (FC) between OB and osteocyte (OC) state.

Gene name	Protein name	Welch's *t*-test *q*-value	FC OB vs OC
MAGEA9	Melanoma-associated antigen 9	<0.00001	3739.16
NUCKS1	Nuclear ubiquitous casein and cyclin-dependent kinase substrate 1	<0.00001	93.13
SUMO2	Small ubiquitin-related modifier 2	0.0011	44.04
CFDP1	Craniofacial development protein 1	0.0001	37.82
HMGB3	High mobility group box 3	0.0000	30.64
PDAP1	28 kDa heat- and acid-stable phosphoprotein	0.0010	21.93
TMSB10	Thymosin beta-10	0.0004	19.26
ANP32B	Acidic leucine-rich nuclear phosphoprotein 32 family member B	0.0000	15.61
WBP11	WW domain-binding protein 11	0.0000	15.29
NCKAP1	Nck-associated protein 1	0.0001	15.25
ZRANB2	Zinc finger Ran-binding domain-containing protein 2	0.0000	14.92
RPL19	Ribosomal protein L19	0.0000	13.73
TBC1D5	TBC1 domain family member 5	0.0000	13.19
LBH	Protein LBH	0.0001	11.68
LRRFIP1	LRR binding FLII interacting protein 1 (Fragment)	0.0019	11.50
ATOX1	Copper transport protein ATOX1	0.0000	11.16
PPP1R14B	Protein phosphatase 1 regulatory subunit 14B	0.0000	10.66
NASP	Nuclear autoantigenic sperm protein	0.0011	10.61
IAH1	Isoamyl acetate-hydrolyzing esterase 1 homolog	0.0005	10.47
BIN1	Myc box-dependent-interacting protein 1	0.0000	10.13

**Table 2:** Top 20 proteins with increased abundance in OC-like cells compared with OB. Gene name and protein name are indicated as well as calculated *q*-values and fold changes (FC) between osteoblast (OB) and OC cell state.

Gene name	Protein name	Welch's T-test *q*-value	FC OC vs OB
THBS1	Thrombospondin-1	0.0000	80.19
BMP4	Bone morphogenetic protein 4	0.0002	69.42
COL12A1	Collagen type XII alpha 1 chain	0.0000	45.46
THBS2	Thrombospondin 2	0.0006	43.04
SEMA3B	Semaphorin 3B	0.0000	42.75
PRELP	Prolargin	0.0000	42.51
LUM	Lumican	0.0000	24.37
PI15	Peptidase inhibitor 15	0.0004	23.68
GLS	Glutaminase	0.0083	22.59
MFGE8	Milk fat glOBule EGF and factor V/VIII domain containing;Lactadherin	0.0000	21.17
TMEM63A	CSC1-like protein 1	0.0000	21.04
NIPSNAP1	Protein NipSnap homolog 1	0.0024	20.97
APOM	Apolipoprotein M	0.0005	20.23
PLCD1	1-phosphatidylinositol 4,5-bisphosphate phosphodiesterase delta-1	0.0003	19.72
STOML2	Stomatin-like protein 2, mitochondrial	0.0029	18.21
MATN2	Matrilin-2	0.0001	16.83
SERPINE2	Glia-derived nexin	0.0000	16.13
WNT11	Protein Wnt-11	0.0021	15.97
ITIH2	Inter-alpha-trypsin inhibitor heavy chain H2	0.0000	15.82
RIOK1	Serine/threonine-protein kinase RIO1	0.0003	15.74

Proteins enriched in OC-like cells were predominantly associated with extracellular matrix organization, cell adhesion, and osteogenesis. These included thrombospondin-1 and 2 (THBS1, THBS2), semaphorin 3B (SEMA3B), prolargin (PRELP), lumican (LUM), matrilin-2 (MATN2), and the collagen isoforms collagen type X alpha 1 chain (COL10A1), collagen type XI alpha 1 chain (COL11A2), and collagen type XII alpha 1 chain (COL12A1). Bone morphogenetic protein 4 (BMP4) also showed markedly increased abundance in OC-like cells, consistent with its established role in osteogenesis [13]. These protein-level changes support the GO-term enrichment results and demonstrate extensive extracellular matrix remodeling during differentiation.

Because SaOS-2 cells are derived from osteosarcoma, we compared their proteomic profiles with a published dataset of non-malignant human bone marrow-derived osteoblasts (hOB) reported by Aasebø et al. [14]. The hOB dataset comprised 5346 proteins, compared with 4213 proteins in SaOS-2 OB and 4284 proteins in OC-like cells. A total of 2838 proteins were shared across all three datasets, while 3008 proteins overlapped between hOB and SaOS-2 OB and 3017 between hOB and OC-like cells (Supplementary Fig. S1). This broad proteomic overlap supports the biological relevance of SaOS-2 cells as a model of hOB biology, while their osteosarcoma origin and incomplete acquisition of a mature osteocyte phenotype must be considered when interpreting differentiation-associated changes.

### 3.2 Proteomic characterization of established osteoblast and osteocyte markers

Previous transcriptomic studies of SaOS-2 differentiation have established a panel of marker genes commonly used to distinguish OB from OC. Because mRNA abundance does not necessarily reflect protein abundance or activity, we examined whether these established markers showed the expected abundance profiles at the protein level (Fig. 3; Supplementary Table S6) [7,15].

The early OB marker RUNX2 showed the expected pattern, with the highest abundance in OB and progressively lower levels in IM and OC-like cells. COL1A1 differed from the previously reported transcript-level profile, showing its highest protein abundance in OC-like cells rather than in OB or IM cells.

**Fig. 3:**
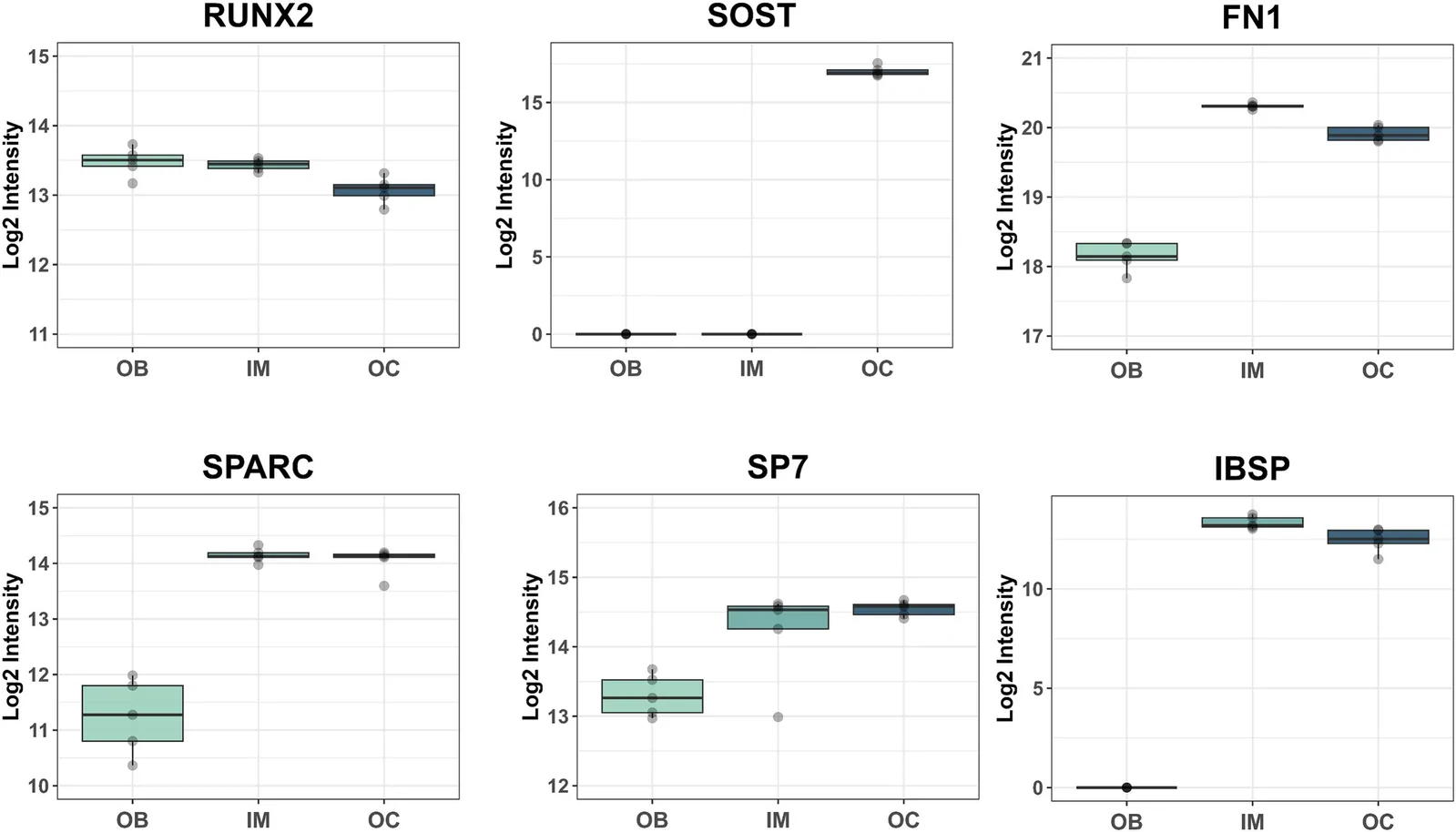
Protein abundance profiles of established osteoblast- and osteocyte-associated markers during SaOS-2 differentiation. Selected literature-derived markers were evaluated in osteoblasts (OB), intermediate cells (IM), and osteocyte-like cells (OC-like cells) based on log2-transformed protein intensities (n = 5 per differentiation state).

Several markers associated with mature OB, including fibronectin (FN1), secreted protein acidic and cysteine rich (SPARC), SP7 transcription factor (SP7/Osterix), and integrin-binding sialoprotein (IBSP), were also quantified [16,17]. Their highest protein abundances were observed in OC-like cells. This pattern indicates that differentiated SaOS-2 cultures retain features of mature OB and do not fully acquire the molecular phenotype of mature OC.

This interpretation was further supported by the absence of several established OC markers, including phosphate-regulating neutral endopeptidase (PHEX) and the SIBLING family proteins dentin matrix protein 1 (DMP1) and matrix extracellular phosphoglycoprotein (MEPE). These proteins were not detected in the analyzed cell lysates, although their transcripts have previously been reported during SaOS-2 differentiation [7].

By contrast, sclerostin (SOST), an established OC-associated marker, was not detected in OB or IM cells but showed high abundance in OC-like cells. The combined marker profile therefore supports differentiation toward an OC-like state while also indicating incomplete acquisition of a mature OC phenotype.

### 3.3 Collagen remodeling accompanies osteoblast-to-osteocyte-like differentiation

Collagens constitute the major structural framework of the bone extracellular matrix and play essential roles in matrix organization, mineralization, and bone cell differentiation. We therefore investigated whether OB-to-OC-like differentiation is accompanied by changes in collagen composition that reflect extracellular matrix remodeling.

To this end, collagen family proteins were compared across OB, IM, and OC-like cells (Fig. 4; Supplementary Table S6). Fifteen collagen isoforms were identified, revealing extensive remodeling of the extracellular matrix during differentiation. Most collagen proteins progressively accumulated in OC-like cells, consistent with ongoing matrix maturation.

In contrast, collagen alpha-1(VI) (COL6A1), collagen alpha-2(VI) (COL6A2), and collagen alpha-1(VII) (COL7A1) showed their highest abundance in OB. This finding suggests that these collagen isoforms contribute primarily to early extracellular matrix organization before acquisition of the OC-like phenotype.

**Fig. 4:**
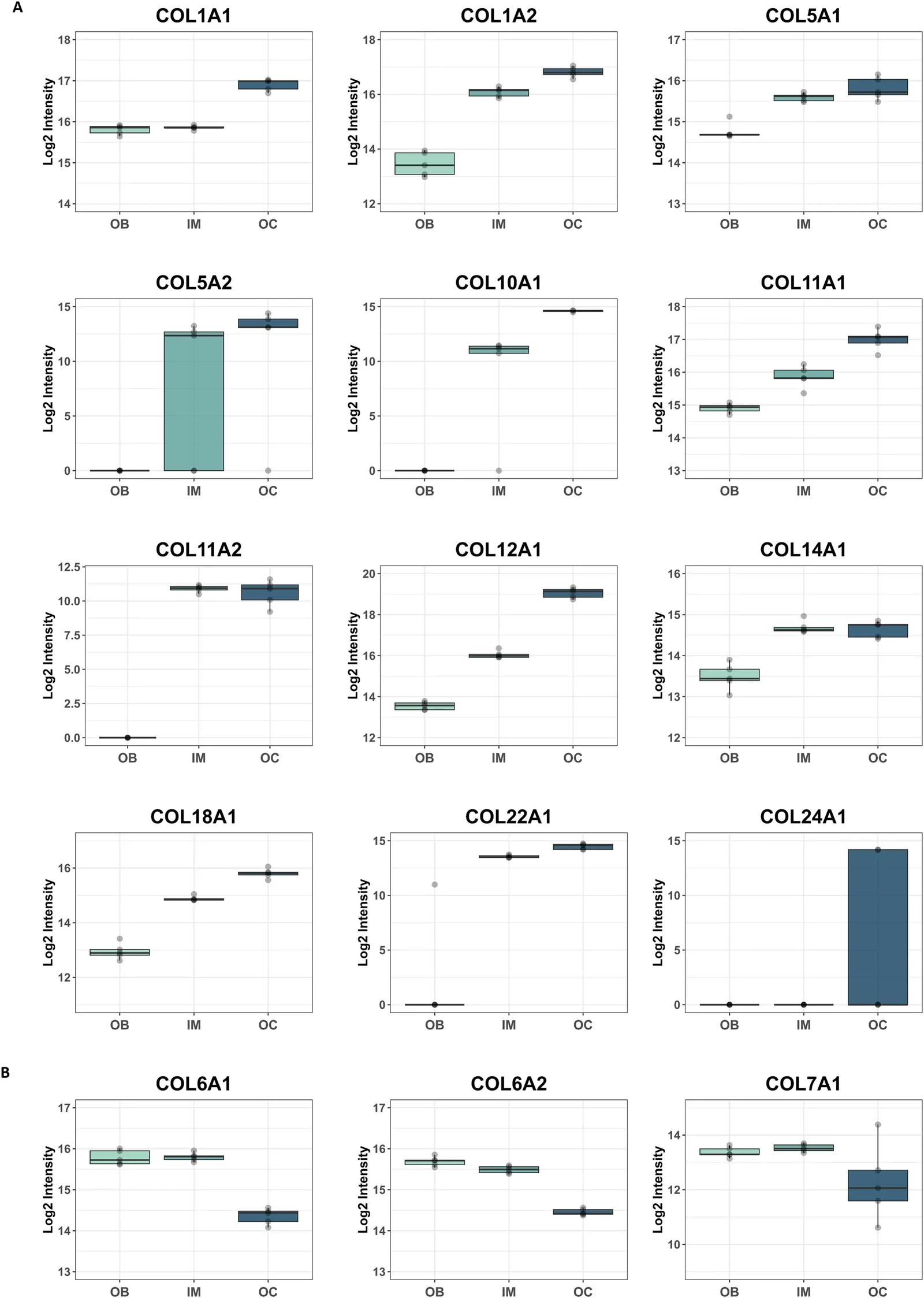
Remodeling of collagen family proteins during SaOS-2 differentiation. Log2-transformed protein abundances of collagen isoforms identified in at least two of five biological replicates are shown for osteoblasts (OB), intermediate cells (IM), and osteocyte-like cells (OC-like cells) (n = 5 per differentiation state). (A) Collagen isoforms showing progressively increasing abundance during differentiation. (B) Collagen isoforms displaying highest abundance in OB or IM compared with OC-like cells.

To validate the proteomic data, COL6A1 abundance was quantified by Western blotting in an independent biological cohort. Consistent with the proteomic analysis, COL6A1 showed significantly higher abundance in OB than in OC-like cells (Supplementary Fig. S3).

Together, these findings demonstrate that OB-to-OC-like differentiation is accompanied by coordinated remodeling of the collagen network rather than uniform of individual collagen isoforms, reflecting progressive maturation of the extracellular matrix regulation.

### 3.4 Novel protein markers distinguish OB and OC-like cell states

Beyond validating established differentiation markers, our proteomic dataset identified additional proteins displaying highly stage-specific abundance patterns. These proteins represent candidate markers that may further refine the molecular characterization of OB and OC-like cells.

Nine proteins were exclusively detected in OB, whereas 48 proteins were exclusively detected in OC-like cells (Supplementary Table S7). These candidate on/off markers were subsequently evaluated by targeted parallel reaction monitoring (PRM) to validate their stage-specific detection.

PRM analysis confirmed several proteins as preferential markers of either the OB or OC-like state (Fig. 5A–C and I–K; Supplementary Methods; Supplementary Figs. S4–S21). A-kinase anchoring protein 12 (AKAP12), caveolin-1 (CAV1), and calponin-1 (CNN1) consistently marked the OB state, whereas aldehyde dehydrogenase 1 family member L2 (ALDH1L2), COL1A1, cysteine rich secretory protein LCCL domain containing 1 (CRISPLD1), fatty acid binding protein 3 (FABP3), growth differentiation factor 6 and 15 (GDF6, GDF15), and hedgehog-interacting protein (HHIP) were preferentially detected in OC-like cells.

**Fig. 5:**
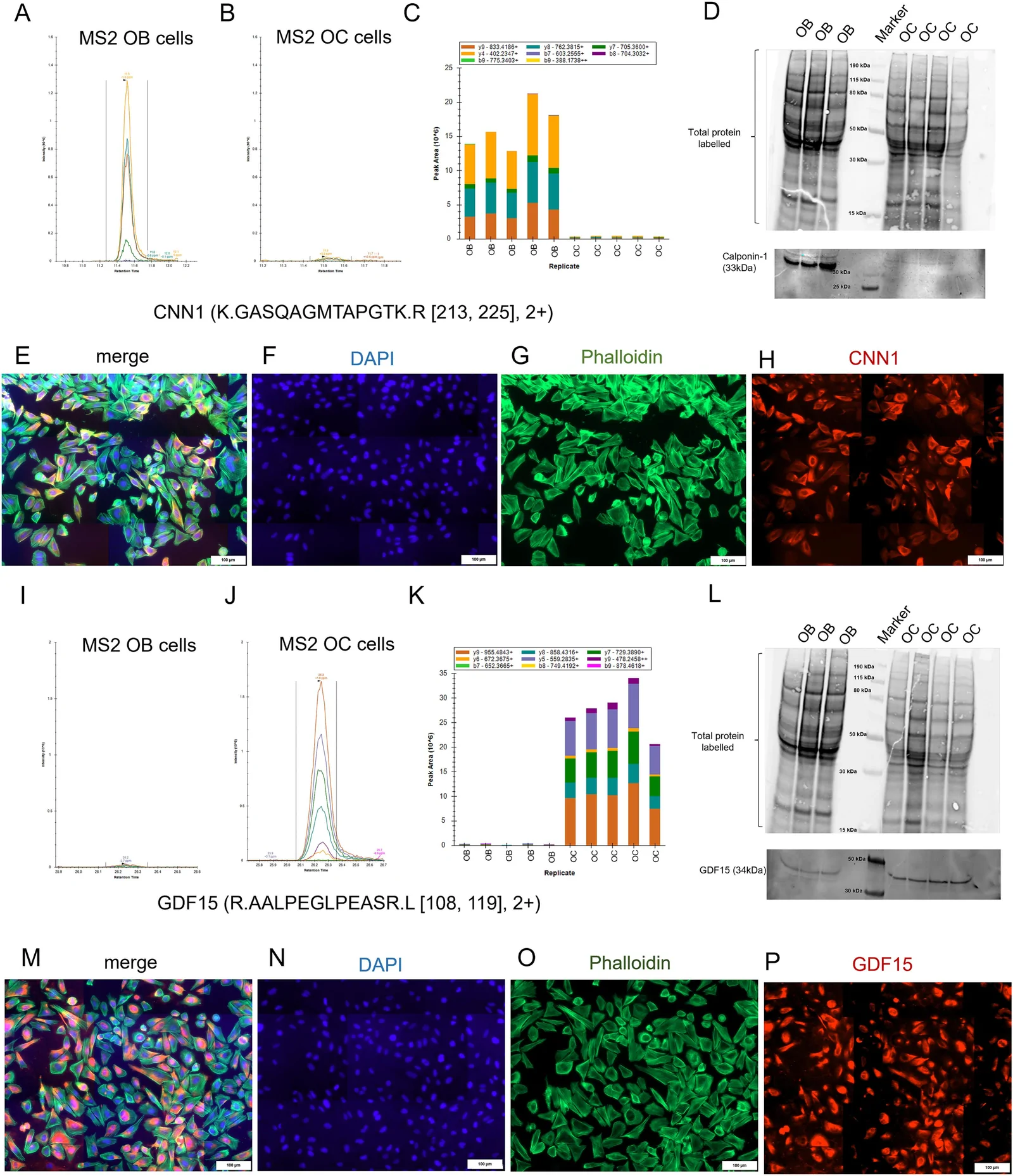
Validation of candidate osteoblast (OB) and osteocyte (OC)-like marker proteins. (A–C) Parallel reaction monitoring (PRM) validation of calponin-1 (CNN1) using the representative peptide GASQAGMTAPGTK. (D) Quantitative Western blot validation of CNN1. (E–H) Immunocytochemical validation of CNN1. (I–K) PRM validation of growth differentiation factor 15 (GDF15) using the representative peptide AALPEGLPEASR. (L) Quantitative Western blot validation of GDF15. (M–P) Immunocytochemical detection of GDF15.

To further validate the targeted proteomics results, CNN1 and GDF15 were selected for quantitative Western blotting and immunocytochemistry using an independent biological cohort (Fig. 5D–P; Supplementary Figs. S21 and S22).

CNN1 was confirmed as an OB-specific marker by both orthogonal approaches, supporting its suitability as a robust marker of the OB state (Fig. 5D–H). In contrast, GDF15 was also detected in OB by Western blotting and immunocytochemistry, indicating that although enriched in OC-like cells, it does not represent a strictly OC-like-specific marker (Fig. 5L–P).

## 4 Discussion

Due to the limited availability and characterization of human bone cell models, research on bone biology and bone-related disorders has largely relied on animal models (Beck-Nielsen et al., 2019). Although these models have provided important insights into skeletal development and disease, they only partially reflect human bone biology and offer limited opportunities for studying disease initiation or introducing disease-associated genetic alterations. Consequently, there is growing interest in human in vitro models that enable precise genetic manipulation and mechanistic investigations.

Among the available models, SaOS-2 cells are unique in their ability to differentiate toward an OC-like phenotype. However, despite their widespread use, their molecular characterization at the protein level has remained incomplete. To address this gap, we generated a comprehensive proteomic roadmap of SaOS-2 differentiation from OB to OC-like cells.

Progressive matrix mineralization, demonstrated by increased calcium staining, provided initial evidence of successful SaOS-2 differentiation. This transition was accompanied by extensive proteomic remodeling across the three differentiation states. Proteins enriched in OB were mainly associated with chromatin organization and proliferative cellular functions, whereas proteins with higher abundance in OC-like cells were linked to extracellular matrix organization, cell adhesion, ossification, and other osteogenic processes. Together, these changes support a transition from an OB toward a functionally distinct OC-like state.

We next examined established transcript- and protein-level markers of OB-to-OC differentiation. Although SaOS-2 cells underwent substantial phenotypic and proteomic changes over 35 days, their marker profile did not fully correspond to that of mature osteocytes. Several proteins commonly associated with mature OB, including FN1, SP7, SPARC, and IBSP, showed their highest abundance after 35 days of differentiation. However, SP7, SPARC, and IBSP have also been detected at the protein level in human adipose stem cell-derived OC-like cells and in the murine OC cell line MLO-Y4 (Santos et al., 2021). Their persistence in differentiated SaOS-2 cells may therefore reflect overlapping molecular features of late OB and OC rather than a failure of differentiation. Among these proteins, SPARC showed particularly high abundance in both OC-related model systems, supporting its association with the differentiated bone cell phenotype, although not its specificity as an OC marker.

Several proteins commonly associated with mature OC, including PHEX, DMP1, and MEPE, were reported at the transcript level (Prideaux et al., 2014) but were not detected in the proteomic analysis of differentiated SaOS-2 cells. This discrepancy may reflect differences between transcript and protein abundance, but it may also be influenced by technical factors. PHEX is membrane-associated, whereas DMP1 and MEPE are extracellular proteins, and both classes can be underrepresented in conventional whole-cell lysates.

Future studies could therefore combine optimized extraction protocols for membrane-associated proteins with secretome analysis. Such approaches may improve the detection of OC-associated proteins and provide a more complete view of extracellular signaling and intercellular communication during bone cell differentiation (Hamrick, 2012; Song et al., 2019; Poschmann et al., 2022).

The absence of these proteins at the proteome level is however also consistent with a recognized limitation of the SaOS-2 model. Previous studies have shown that differentiated SaOS-2 cells acquire many characteristics of OC but do not fully recapitulate the phenotype of mature OC, including aspects of their endocrine function (Prideaux et al., 2014; Yevlashevskaya et al., 2023).

Several approaches have been proposed to promote further maturation of OC-like cells, including stimulation with BMP-2 (Li et al., 2008; Sun et al., 2015) or other Wnt pathway activators, mechanical stimulation in three-dimensional culture systems, genetic or epigenetic manipulation, and exposure to conditioned medium from primary OB. In addition, modulation of 1,25-dihydroxyvitamin D3 levels has been reported to induce fibroblast growth factor 23 (FGF23) protein expression (Wu et al., 2007; Gayan-Ramirez and Janssens, 2021), further supporting the concept that external physiological cues may promote a more mature OC-like phenotype.

Despite the limitations of SaOS-2 model, our proteomic analysis identified several candidate markers that may support the assessment of successful differentiation (see Fig. 6). Most proteins with stage-specific on/off patterns have previously been linked to bone cell biology, although their potential cell state-specific abundance has not been systematically described.

**Fig. 6:**
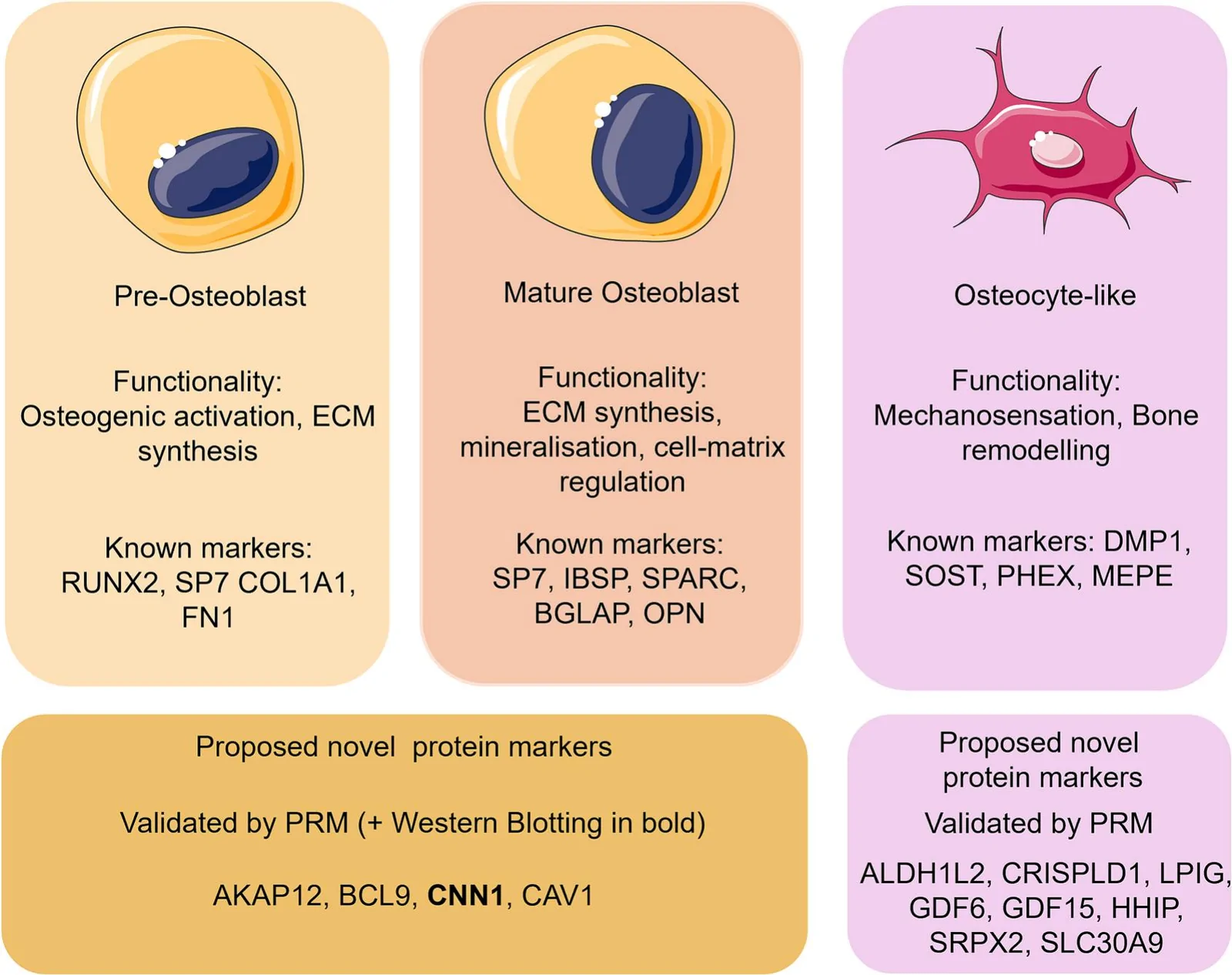
Overview of osteoblast differentiation toward osteocyte-like cells and candidate protein markers identified in this study. The schematic summarizes the major functional characteristics and established marker proteins of pre-osteoblasts, mature osteoblasts, and osteocytes. In addition, candidate protein markers identified by quantitative proteomics for the osteoblast (OB) and osteocyte-like (OC-like) states are shown. Selected candidates were validated by parallel reaction monitoring (PRM), Western blotting (bold), and immunocytochemistry. Illustrations were adapted from Servier Medical Art (https://smart.servier.com) and are licensed under CC BY 4.0 (https://creativecommons.org/licenses/by/4.0/).

Candidate proteins associated with the OB state included AKAP12, a regulator of bone remodeling (Chen et al., 2024); BCL9, a Wnt/β-catenin transcriptional cofactor involved in OB differentiation (Orikasa et al., 2022); CAV1 which has been linked to purinergic receptor signaling in OB (Gangadharan et al., 2015); and CNN1, which has been implicated in OB function and osteoclast formation [27]. AKAP12, CAV1, and CNN1 were also detected in a published dataset of human bone marrow-derived osteoblasts (hOB) (Aasebø et al., 2021), supporting their association with the OB state.

PRM confirmed the detection of AKAP12, CAV1, and CNN1 in OB. CNN1 was further validated by Western Blotting and immunohistochemistry in an independent biological cohort. These findings identify AKAP12, CAV1, and particularly CNN1 as promising protein-level indicators of the OB state that may complement established transcriptional markers.

In addition to the established OC-associated marker SOST, our data identified several candidate markers of the OC-like state, including ALDH1L2, CRISPLD1, endothelial lipase (LIPG) (Rutkowsky et al., 2024), FABP3, GDF6 (Tam et al., 2025), GDF15 (Westhrin et al., 2015), HHIP (Smith et al., 2023), sushi repeat-containing protein X-linked 2 (SRPX2), and zinc transporter 9 (SLC30A9) (Huang and Tepaamorndech, 2013). Several of these proteins have previously been linked to bone biology. For example, GDF15 has been associated with bone remodeling under hypoxic conditions (Hinoi et al., 2012), whereas HHIP has been implicated in ossification and skeletal development (Alman, 2015). However, GDF15 is also expressed in a variety of human cancers (Kim et al., 2008; Strelau et al., 2008; Zhang et al., 2009), illustrating one of the inherent limitations of using an osteosarcoma-derived cell line as a differentiation model.

Interestingly, ALDH1L2, CRISPLD1, GDF6, GDF15, and SRPX2 were also detected in the OC-like cell line MLO-Y4 (Santos et al., 2021), supporting their association with a differentiated OC-like phenotype. PRM analysis further confirmed ALDH1L2, COL1A1, CRISPLD1, FABPH, GDF15, HHIP, and SRPX2 as candidate markers preferentially detected in OC-like cells. For several of these proteins, including ALDH1L2, CRISPLD1, FABP3, COL1A1, and SRPX2, little is currently known about their potential roles in osteocyte biology. Although GDF15 showed increased abundance in OC-like cells in the discovery proteomics and PRM analyses, orthogonal validation detected GDF15 expression in both OB and OC-like cells. GDF15 therefore appears to reflect differentiation-associated changes rather than representing a specific marker of the OC-like state.

Beyond the candidate markers discussed above, our proteomic data revealed pronounced changes in extracellular matrix-associated proteins, particularly collagens. Although collagen type I is the predominant structural component of bone, the roles and differentiation-dependent abundance patterns of many other collagen isoforms remain incompletely understood.

Several collagen proteins showed distinct abundance profiles across the differentiation states. COL5A2, COL10A1, COL11A2, and COL22A1 were not detected in OB but were present in OC-like cells, whereas COL6A1, COL6A2, and COL7A1 showed their highest abundance in OB. Although the latter proteins were also detected in OC-like cells, their lower abundance after differentiation suggests a stronger association with the early OB state and initial extracellular matrix organization.

The higher abundance of COL6A1 in OB was confirmed by Western blotting in an independent biological cohort. These findings indicate that OB-to-OC-like differentiation involves coordinated remodeling of the collagen network and highlight extracellular matrix composition as an underexplored molecular feature of bone cell differentiation. Whether individual collagen isoforms can serve as reliable differentiation markers will require validation in additional human bone cell models.

Interestingly, previous studies have already implicated COL6A1 in osteogenesis. Mutations in collagen VI genes cause Ullrich congenital muscular dystrophy (UCMD) and Bethlem myopathy (BM), disorders that are also associated with skeletal abnormalities. During OB differentiation, COL6A1 has been shown to localize to matrix bridges connecting adjacent cells, and the formation of these structures is impaired in COL6A1-deficient OB (Izu et al., 2016). Similarly, COL12A1 has been identified as an essential component of matrix bridge formation, which is particularly interesting given its increased abundance in OC-like cells observed in our study. Consistent with these findings, COL6A1-deficient mice exhibit osteopenia and abnormal osteoblast morphology (Izu et al., 2012). Together, these observations support the concept that collagen remodeling is not merely a consequence of differentiation but may actively contribute to the establishment and maintenance of the OB and OC extracellular matrix.

In summary, our study supports SaOS-2 cells as a useful in vitro model for investigating the transition from an OB to an OC-like state. The comparison of established differentiation markers at the transcript and protein levels revealed substantial differences between both molecular layers, highlighting the added value of proteomic analyses for characterizing bone cell differentiation. We further identified several candidate protein markers associated with the OB and OC-like states and generated a comprehensive proteomic roadmap of SaOS-2 differentiation. Together, these findings provide a molecular resource for future studies of bone formation, extracellular matrix remodeling, and skeletal disease.

## 5 Limitations

This study has several limitations. It was designed primarily to characterize proteomic changes during SaOS-2 differentiation; functional experiments addressing the mechanistic roles of individual candidate proteins were therefore beyond its scope. The proposed markers should consequently be validated in additional human bone cell models and primary material before broader applicability can be established.

We also analyzed whole-cell lysates and did not assess the SaOS-2 secretome. Secretome profiling could provide complementary information on extracellular signaling and differentiation status and may improve the detection of secreted OC-associated proteins such as FGF23.

The SaOS-2 model itself has inherent limitations. These cells originate from an osteosarcoma and acquire an OC-like rather than a fully mature OC phenotype. Their proteomic profile may therefore reflect both differentiation-associated changes and features related to their malignant origin. In addition, the present study examined a single cell line and differentiation protocol, which limits the generalizability of the identified protein patterns.

Despite these constraints, SaOS-2 cells offer several practical advantages. Their differentiation can be standardized, they can be maintained over extended periods, and they are amenable to genetic manipulation. Compared with primary cells, which show limited expansion capacity and substantial donor variability, and induced pluripotent stem cell-derived models, which are more resource-intensive and may yield heterogeneous differentiated populations, SaOS-2 cells provide a reproducible system for initial screening and proof-of-concept studies. They therefore occupy a useful position between reductionist cell culture systems, primary human material, and animal models.

## CRediT authorship contribution statement

**Britta Eggers:** Writing – review & editing, Writing – original draft, Validation, Project administration, Methodology, Investigation, Formal analysis, Data curation, Conceptualization. **Sascha Roocke:** Writing – review & editing, Methodology, Investigation, Formal analysis, Conceptualization. **Svitlana Rozanova:** Writing – review & editing, Visualization, Software, Formal analysis. **Karin Schork:** Writing – review & editing, Visualization, Software, Formal analysis. **Maximilian Schütte:** Writing – review & editing, Validation, Investigation. **Mira Struwe:** Writing – review & editing, Validation, Investigation. **Paul Zandner:** Writing – review & editing, Validation, Investigation. **Maximilian Hausherr:** Writing – review & editing, Methodology, Validation. **Greta Domroese:** Writing – review & editing, Validation, Investigation. **Morris Gellisch:** Writing – review & editing, Investigation. **Martin Eisenacher:** Writing – review & editing, Software, Resources, Funding acquisition. **Jacqueline Heinen-Weiler:** Writing – review & editing, Validation, Resources, Methodology. **Carsten Theiss:** Writing – review & editing, Resources, Funding acquisition. **Annette Richter-Unruh:** Writing – review & editing, Project administration, Funding acquisition. **Katalin Barkovits:** Writing – review & editing, Supervision, Project administration, Funding acquisition, Conceptualization. **Katrin Marcus:** Writing – review & editing, Project administration, Funding acquisition, Conceptualization.

## Declaration of Generative AI and AI-assisted technologies in the writing process

During the preparation of this work, the authors used ChatGPT to improve the clarity and readability of the manuscript, including shortening sentences, and improving the use of clear and concise language. After using this tool, the authors reviewed and edited the content as required and take full responsibility for the content of the published article.

## Funding

This research was funded by “Georg E. and Marianne Kosing-Stiftung” (T0503/37590/2021/sm), whose generous support made this project possible.

This work was supported by the Protein Research Unit Ruhr within Europe (PURE), funded by the Ministry of Innovation, Science and Research (10.13039/501100009591MIWF) of North Rhine-Westphalia, Germany (grant no. 233-1.08.03.03-031-68079), and by the Center for Protein Diagnostics (PRODI), funded by the Ministry of Culture and Science (MKW) of the State of North Rhine-Westphalia, Germany (grant no. 111.08.03.05-133974).

Additional support was provided by the German Network for Bioinformatics Infrastructure (de.NBI/ELIXIR-DE, W-de.NBI-005) and CUBiMed.RUB, a scientific infrastructure center of the Medical Faculty of Ruhr University Bochum.

Parallel reaction monitoring was performed using a Vanquish Neo UHPLC–Orbitrap Exploris 480 mass spectrometer funded by the German Federal Ministry of Education and Research (BMBF; grant no. 13N16290) within the Federal Security Research Programme (www.sifo.de).

## Declaration of competing interest

The authors declare the following financial interests/personal relationships which may be considered as potential competing interests: Carsten Theiss, Annette Richter-Unruh, Katrin Marcus report financial support was provided by Georg E. and Marianne Kosing-Stiftung. If there are other authors, they declare that they have no known competing financial interests or personal relationships that could have appeared to influence the work reported in this paper.

## Data Availability

The mass spectrometry proteomics data have been deposited to the ProteomeXchange Consortium via the PRIDE partner repository (Perez-Riverol et al., 2025) with the dataset identifier PXD073069.

## Appendix A Supplementary data

Supplementary figures

Supplementary Table S1 Primary and secondary antibodies, including information on vendor, purchase number and lot number as well as dilutions.

Supplementary Table S2 Quantified proteins in osteoblasts (OB) intermediate cell state (IM) and osteocytes (OC). Information on protein accession, gene name and protein name are given. Displayed are the LOESS normalized log2 intensities for each protein in each sample.

Supplementary Table S3 Quantification of calcium production by alizarin red staining and measurement of staining intensity at 470 nm. Measurements of cell culture supernatant was conducted for osteoblasts (OB,0d), intermediate differentiation state (IM, 18d) and osteocytes (OC, 35d)

Supplementary Table S4 ANOVA between all three samples types to determine signficantly regulated proteins. Information on protein accession, gene name and protein name are given. Normalised log2 intensities for each sample are displayed. Statistical evaluation was conducted, whereby an ANOVA was carried out, as well as an adjustment by FDR. To determine signficantly regulated proteins between two samples Tukey posthoc tests were conducted. Fold changes were calculated by substracting average log2 intensities of one groups with the average log2 intensities of the second group. Supplementary Table S4: Heatmap clusters. Information on which protein was sorted into which cluster can be seen in column clusters. Additionally information on protein accession, gene name and protein name are given, as well as log2 intensities for each protein in each sample. Supplementary Table S4: Gene Ontology enrichment analysis of Cluster 1 and 4. Terms were filtered by p-value <0.05.

Supplementary Table S5 Differentially abundant proteins between osteoblasts and osteocyte-like cells, osteoblasts and intermediate cells and intermediate and osteocyte-like cells, including the Top100 proteins being of higher abundance (based on fold change)) Information on protein accession, gene name and protein name are given. Statistical evaluation was carried out by conducting Welch's t-tests. Resulting p-values were adjusted by Benjamini Hochberg resulting in q-values. The Welch's t-test difference corresponds to the log2 fold change, calculated by subtracting average log2 intensities of one groups with the average log2 intensities of the second group.

Supplementary Table S6 Normalized log2 intensities on the basis of mass spectrometric measurements of of known mRNA markers of osteoblasts (OB) intermediate cell state (IM) and osteocytes (OC) and all identified collagens. Information on gene name and corresponding log2 intensities for every replicate of OB, IM and OC cells are given.

Supplementary Table S7 Determination of on and off proteins in osteocytes (OC) compared to osteoblasts (OB) and the intermediate cell state (IM) on the basis of quantified proteins in data dependent- and data independent mass spectrometric measurements. Information on protein accession, protein name and gene name are given.
