# Supplementary material for: A proteomic roadmap of SaOS-2 osteoblast-to-osteocyte-like differentiation
Source: Bone Rep. 2026 Oct 23;31:101961. doi: 10.1016/j.bonr.2026.101961 (PMC13722065; doi:10.1016/j.bonr.2026.101961)
Supplement: Supplementary file 1 [file mmc1.pdf]

Global LC-MS analyses

Peptide concentration was determined via amino acid analysis and 200 ng of the digested sample was utilized for global LC-MS/MS analyses. For mass spectrometric measurement of the global studies, data-independent acquisition (DIA) was performed.Liquid chromatography (LC) was performed on an Ultimate 3000 RSLC nano-LC system (Thermo Fisher Scientific, Bremen, Germany), which was online-coupled to an Orbitrap Fusion Lumos Tribrid mass spectrometer (Thermo Fisher Scientific, Bremen Germany). Heated Trap-and Elute Injection was used to load and pre-concentrated the peptides onto the trap column (Acclaim™PepMap™ 100 C18, 100 μm ID x 2 cm, particle size 5 μm, pore size 100 Å, Thermo Fisher Scientific). Then, using a DNV PepMap™ 1500 bar C18 analytical column (75 mm x 150 mm, 2 mm particle size, 100 Å pore size, Thermo Fisher Scientific), the peptide separation was carried out. Peptides were separated using a linear gradient using 5 to 40% solvent B (solvent A: 0.1% FA, solvent B: 0.1% FA and 0.1% FA), with a flow rate of 400 nL/min for 60 min. DIA MS scans were acquired from 350 to 1400 m/z at a resolution of 120000 at 200 m/z for the detection of precursor ions (normalized AGC target 300 %, 54 ms maximum injection time). For fragmentation, the mass window was divided in 46 windows and MS/MS fragments were generated by high-energy collision-induced dissociation (HCD) with normalized collision energy (NCE) of 30. The fragments were analyzed in an Orbitrap analyzer with a 15000 resolution at 200 m/z (AGC Standard, maximum injection time 130 ms).

For spectral library generation data dependent acquisition was performed. For that approximately 200ng of peptides were subjected to LC-MS/MS. Liquid chromatography (LC) was performed using the Vanquish Neo UHPLC system (ThermoFisher Scientific, Bremen, Germany). Samples were loaded on a a precolumn (PepMap Neo C18 Trap Cartridge 300 μm × 0.5 cm, particle size 5 μm) and then separated on an analytical column (DNV PepMapTM Neo, 75 μm x 150 mm, C18, particle size 2 μm, pore size 100 Å). The peptides were separated at a flow rate of 400 nl/min and a solvent gradient of 1% B to 22% B (B: 84 % acetonitrile, 0.1% formic acid) for 40 min and a subsequent increase to 40% for 20min. The column was rinsed with 99 % B for 5 min. The chromatography was online coupled to an Exploris480 mass

spectrometer (ThermoFisher Scientific, Bremen Germany). Prior entering the mass spectrometer peptides were ionised by ESI. The capillary temperature was set to 275°C and the spray voltage to 1800 V. MS1 spectra were recorded in the range from 350 to 1500 m/z with a resolution of 120,000 at 200 m/z (normalised AGC-target 250%, maximum injection time 150 ms. The Top25 peptides were chosen for MS2 fragmentation and set on a dynamic exclusion list for 80 s. MS2 spectra were acquired at 30,000 resolution with a normalised AGC target of 150 % and a maximum injection time of 50 ms. Fragmentation was achieved by HCD and a normalized collision energy of 31%.

Parallel reaction monitoring (PRM)

PRM measurements were conducted to confirm identified on/off proteins from DIA analysis. Comparable to DIA analysis, 200ng of peptides were utilized for mass spectrometric measurements. Liquid chromatography (LC) was performed using the Vanquish Neo UHPLC system (ThermoFisher Scientific, Bremen, Germany). Samples were loaded on a a precolumn (PepMap Neo C18 Trap Cartridge 300 µm × 0.5 cm, particle size 5 µm) and then separated on an analytical column (DNV PepMapTM Neo, 75 µm x 150 mm, C18, particle size 2 µm, pore size 100 Å). The peptides were separated at a flow rate of 400 nl/min and a solvent gradient of 1% B to 22% B (B: 84% acetonitrile, 0.1% formic acid) for 40 min and a subsequent increase to 41 % for 20min. The column was rinsed with 99% B for 5 min. The chromatography was online coupled to an Exploris480 mass spectrometer (ThermoFisher Scientific, Bremen Germany). Prior entering the mass spectrometer peptides were ionised by ESI. The capillary temperature was set to 275°C and the spray voltage to 1800 V. MS1 spectra were recorded in the range from 350 to 1500 m/z with a resolution of 120,000 at 200 m/z (normalised AGC-target 250%, maximum injection time 150 ms. An inclusion list of 80 peptides was included for PRM analysis (see table 1 below). MS2 scans were acquired at a resolution of 30,000, an isolation window of 0.8 an AGC target of 2500% and an injection time of 50ms. MS2 fragments were generated with a normalized collision energy of 31%. Data analysis was carried out in

Skyline as described in the main manuscript. Results of PRM analysis can be found in table 2 below, as well as in supplementary figure S3-20.

Table 1: Peptide inclusion list for parallel reaction monitoring of potential on/off markers. Information on the corresponding proteins, targeted peptide sequences, peptide charge state, polarity, estimated retention time (RT) in min and definied collision energy (CID) are given.

**Protein**/corresponding peptides	m/z	z	Polarity	RT time (min)	CID Collision Energy (%)
**RFT1**					
LQTLPVSR (light)	457.276.909	2	Positive	19.7	27
**TP53I11**					
HSQTDLVSR (light)	521.767.437	2	Positive	10	27
ILGVGGEDDDGEVHR (light)	523.249.747	3	Positive	21.7	27
ISQVLGNEIK (light)	550.819.138	2	Positive	22.2	27
COL24A1					
NDAYIETPFVK (light)	64.882.716	2	Positive	32.3	27
VNNAFLFSIR (light)	590.827.297	2	Positive	37.3	27
VLSEDTFTEGK (light)	613.300.975	2	Positive	22.5	27
VTGLSLFK (light)	432.763.103	2	Positive	30	27
VTNEPSVDNHLDLR (light)	536.937.392	3	Positive	22	27
**SRPX2**					
GGNYHSSLGTR (light)	574.775.793	2	Positive	8.6	27
GPEPGSHFPEGEHVIR (light)	872.923.717	2	Positive	18.6	27
LLIISAPDPSNR (light)	648.369.526	2	Positive	33.2	27
HVTIIELVGQPPQEVGR (light)	624.682.775	3	Positive	35.3	27
EQQLSANIIEELR (light)	771.909.744	2	Positive	38.9	27
SYFNMVLIDK (light)	615.315.374	2	Positive	39.3	27
**ALDH1L1**					
HGSIIYHPSLLPR (light)	497.280.653	3	Positive	23.3	27
LPQPEEGATYEGIQK (light)	830.414.859	2	Positive	24.8	27
**FABP3**					
SLGVGFATR (light)	454.253.434	2	Positive	23.2	27
QVASMTKPTTIIEK (light)	773.929.091	2	Positive	20.5	27
NGDILTLK (light)	437.255.642	2	Positive	23.4	27
LGVEFDETTADDR (light)	734.333.535	2	Positive	28.6	27
SIVTLDGGK (light)	4.452.531	2	Positive	19.2	27
WDGQETTLVR (light)	602.801.477	2	Positive	23.5	27
**GALNT3**					
TTNLSVEEQK (light)	574.793.317	2	Positive	13.1	27
TVHSVLYSSPAILLK (light)	543.318.774	3	Positive	33.6	27
DQLLYNPFLK (light)	625.842.613	2	Positive	44.3	27
**GDF6**					
VVPHEYMLSIYR (light)	753.892.311	2	Positive	29.4	27
GLDDLSHTPLR (light)	612.322.576	2	Positive	24.9	27
AAWGELDAGEAEAR (light)	723.336.412	2	Positive	27.8	27

GPQQPPPPDLR (light)	601.319.837	2	Positive	18	27
ALLVVFTR (light)	459.792.195	2	Positive	34.7	27
**PUSL1**					
LNSVEPVR (light)	457.258.716	2	Positive	14.2	27
TDAGVHALSNAAHLDVQR (light)	625.653.767	3	Positive	23.4	27
VSVSPGQASPLVTPEESR (light)	920.475.979	2	Positive	29.2	27
TILESQDPLGK (light)	60.082.716	2	Positive	26.6	27
**HHIP**					
QPVGALHSGDGSQR (light)	470.235.772	3	Positive	10.1	27
GLLSLAFHPNYK (light)	453.918.833	3	Positive	33.4	27
LYVSYTTNQER (light)	687.338.423	2	Positive	20.6	27
VVEYTVSR (light)	476.758.549	2	Positive	15.3	27
NGNFLTLQQSPVTK (light)	773.914.829	2	Positive	30.8	27
**GDF15**					
ASFPGPSELHSEDSR (light)	539.249.747	3	Positive	21	27
YEDLLTR (light)	45.523.745	2	Positive	21.4	27
ILTPEVR (light)	414.252.903	2	Positive	19.3	27
AALPEGLPEASR (light)	605.824.952	2	Positive	26.2	27
AANMHAQIK (light)	492.258.193	2	Positive	8.4	27
TDTGVSLQTYDDLLAK (light)	870.438.531	2	Positive	40.6	27
**CRISPLD1**					
AITDNDMQSILDLHNK (light)	609.968.364	3	Positive	34.7	27
AAIHYGIIDNDGGWVDITR (light)	696.016.594	3	Positive	40.1	27
YQSANSFTVSK (light)	616.301.309	2	Positive	17	27
AAVHAGVVR (light)	440.261.593	2	Positive	9.2	27
**PDLIM3**					
PQTVILPGPAPWGFR (light)	545.971.708	3	Positive	45.5	27
LSGGIDFNQPLVITR (light)	815.451.579	2	Positive	41.6	27
VLQGMVDDGSDDRPAGTR (light)	630.298.772	3	Positive	20.6	27
**CCNH**					
FYLNNSVMEYHPR (light)	557.264.563	3	Positive	29.4	27
VDEFNVSSPQFVGNLR (light)	603.303.964	3	Positive	39.6	27
ESPLGQEK (light)	444.227.082	2	Positive	10.2	27
YPILENPEILR (light)	678.879.727	2	Positive	37.4	27
TADDFLNR (light)	476.230.156	2	Positive	20.2	27
**BCL9**					
STAPLNTQISALR (light)	686.383.165	2	Positive	28.3	27
IPVEGPLSPSR (light)	576.324.588	2	Positive	24.9	27
LRPGGSDMLPAQQK (light)	499.932.925	3	Positive	17.5	27
SPPVLGSAAASPVHLK (light)	510.959.469	3	Positive	26.2	27
**CNN1**					
GPAYGLSAEVK (light)	546.290.213	2	Positive	21.2	27
NIIGLQMGTNK (light)	396.885.024	3	Positive	21.2	27
GASQAGMTAPGTK (light)	588.787.512	2	Positive	11.6	27
**AKAP12**					
SAESPTSPVTSETGSTFK (light)	906.928.528	2	Positive	23.5	27
TQEVLQAVAEK (light)	60.833.261	2	Positive	21.6	27

VVGQTTPESFEK (light)	661.335.349	2	Positive	19.3	27
ERPPAPSSFVFQEETK (light)	616.975.735	3	Positive	27.7	27
STPVIVSATTK (light)	552.318.971	2	Positive	18.9	27
GLSSDLEGEK (light)	517.753.661	2	Positive	16.6	27
SVPEDDGHALLAER (light)	754.870.619	2	Positive	21.8	27
CAV1					
YVDSEGHLYTVPIR (light)	5.502.825	3	Positive	27.2	27
AMADELSEK (light)	497.231.507	2	Positive	15.6	27
HLNDDVVK (light)	470.248.349	2	Positive	10.3	27
**MORF4L2**					
NLEPALPGR (light)	483.771.991	2	Positive	23.1	27
SAENPPSGSVR (light)	550.770.176	2	Positive	10.2	27
ADPTVESEEAFK (light)	661.809.164	2	Positive	22.2	27
**ZNF629**					
SFTQSSHLVQHQR (light)	518.930.572	3	Positive	10.8	27
SFIQSSELTQHQR (light)	520.930.349	3	Positive	19.5	27

Table 2: Results of targeted mass spectrometry analysis of determined on/off proteins. Information on protein is indicated by gene name. Fold changes were calculated by dividing area under the curves (AUC) of OB vs OC cells. Thus fold changes > 1 are found to be enriched in OB cells and fold changes < 1 in OC cells. Signficant enrichment was verified by adjusted p-values. Further it was indicated in which condition the proteins is thought to be an on or off protein.

Protein (Gene name)	Fold Change Result OB vs OC (confidence intervall)	Adjusted P-Value	on protein in	off protein in
AKAP12	10.76 (95% CI:7.79 to 14.84)	0.00	OB	OB
BCL9	4.61 (95% CI:3.5 to 6.07)	0.00	OB	OB
CAV1	9.15 (95% CI:7.62 to 10.98)	0.00	OB	OB
CCNH	1.9 (95% CI:1.46 to 2.47)	0.00	OB	OB
CNN1	36.89 (95% CI:28.34 to 48.02)	0.00	OB	OB
MO4L2	3.8 (95% CI:2.99 to 4.84)	0.00	OB	OB
PDLI3	5.58 (95% CI:3.8 to 8.17)	0.00	OB	OB
ZN629	1.56 (95% CI:1.09 to 2.24)	0.02	OB	OB
ALDH1L2	0.03 (95% CI:0.02 to 0.05)	0.00	OC	OB
COL1A1	0.02 (95% CI:0.01 to 0.03)	0.00	OC	OB
CRISPLD1	0.07 (95% CI:0.05 to 0.09)	0.00	OC	OB
FABPH	0.08 (95% CI:0.06 to 0.12)	0.00	OC	OB
GDF15	0.03 (95% CI:0.02 to 0.05)	0.00	OC	OB
GDF6	0.01 (95% CI:0 to 0.02)	0.00	OC	OB
HHIP	0.03 (95% CI:0.02 to 0.04)	0.00	OC	OB
TP53I11	0.13 (95% CI:0.08 to 0.21)	0.00	OC	OB
PUSL1	0.21 (95% CI:0.14 to 0.32)	0.00	OC	OB
RFT1	0.32 (95% CI:0.18 to 0.56)	0.00	OC	OB
SRPX2	0.03 (95% CI:0.03 to 0.04)	0.00	OC	OB
