# Supplementary material for: A proteomic roadmap of SaOS-2 osteoblast-to-osteocyte-like differentiation
Source: Bone Rep. 2026 Oct 23;31:101961. doi: 10.1016/j.bonr.2026.101961 (PMC13722065; doi:10.1016/j.bonr.2026.101961)
Supplement: Supplementary file 2 [file mmc2.pdf]

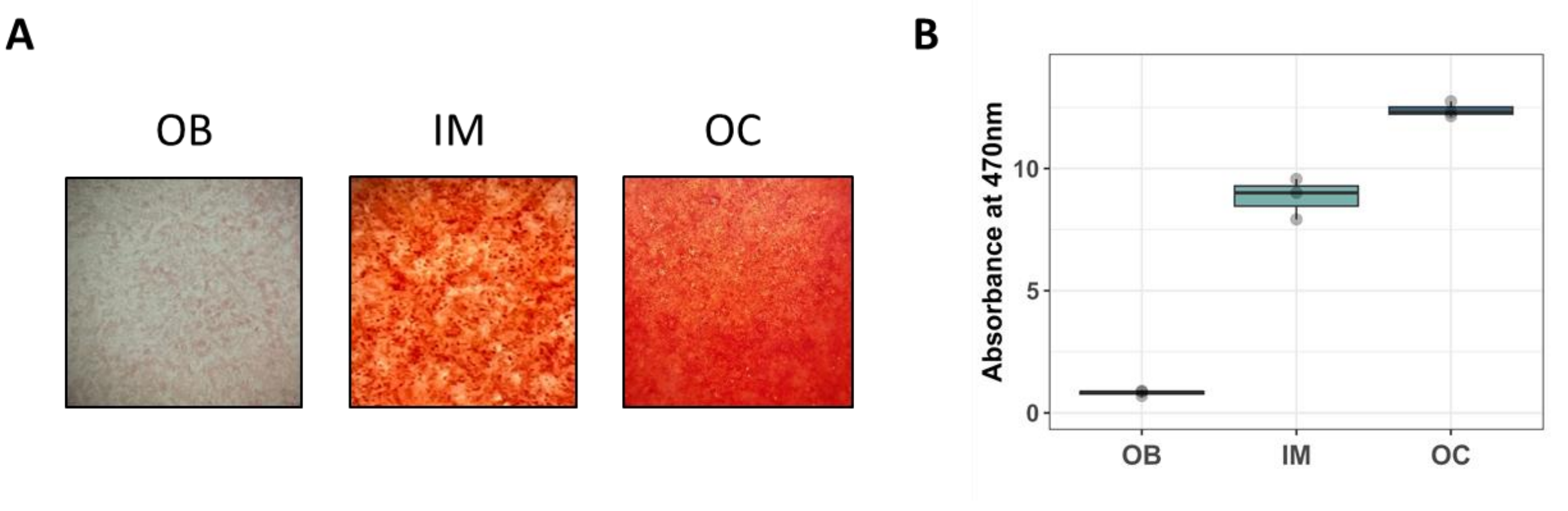

Supplementary figure S1: Verification of SaOS-2 differentiation by calcium staining. Differentiation was assessed in undifferentiated osteoblasts (OB, day 0), intermediate cells (IM, day 18), and osteocyte-like cells (OC-like cells, day 35). (A) Representative images of Alizarin Red S staining (Merck Millipore) demonstrate progressive matrix mineralization during differentiation. (B) Quantification of Alizarin Red S staining by absorbance measurement at 470 nm. Data are shown as box plots (n = 3 per group).

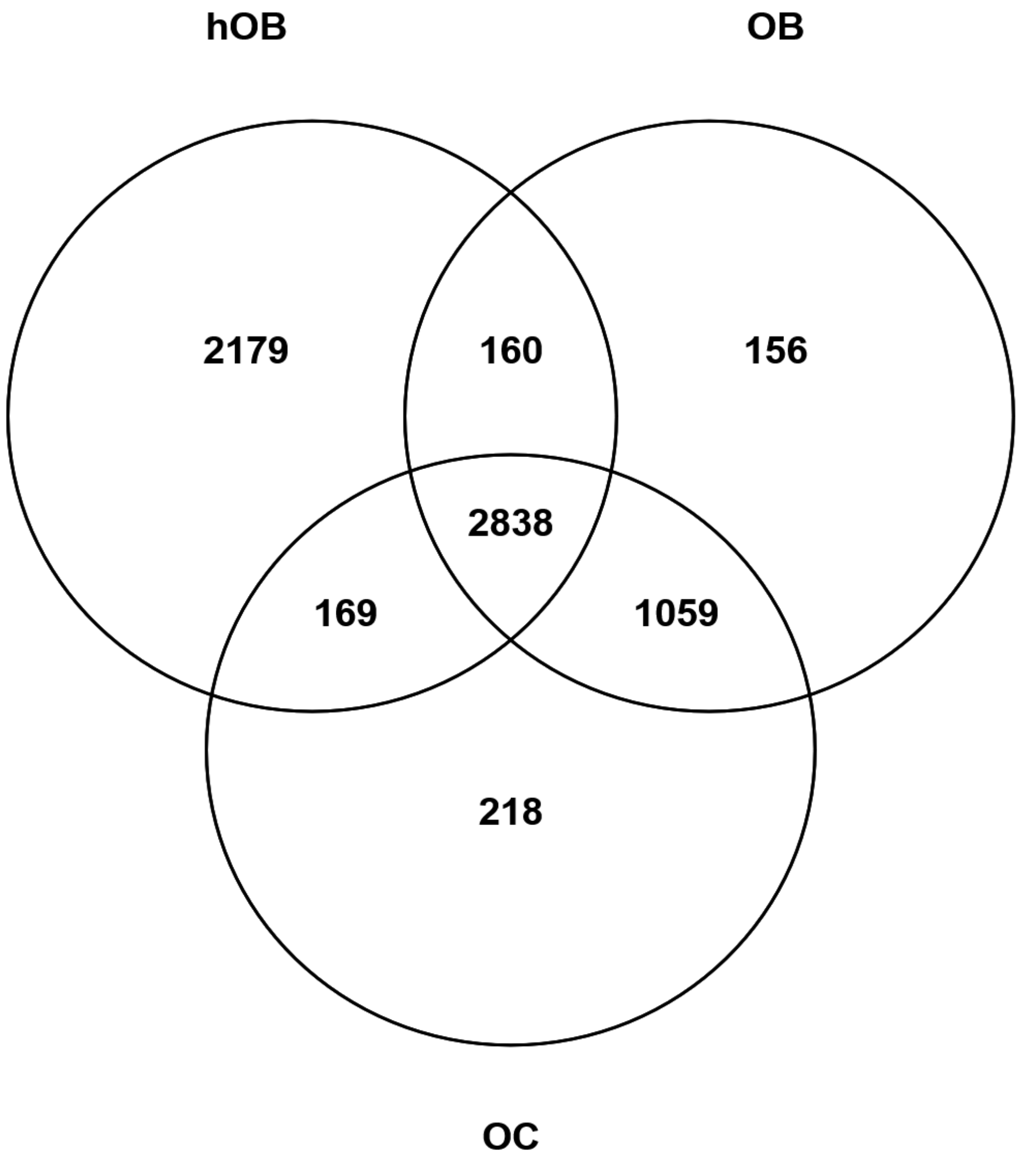

Supplementary figure 2: Comparison of proteome overlaps between datasets of bone cells. Proteins identified in osteoblasts (OB) and osteocytes (OC) identified in our manuscript were compared to human bone marrow derived osteoblasts (hOB) by Aasebø et al, whereby an overlap of 2838 proteins was determined.

COL6A1

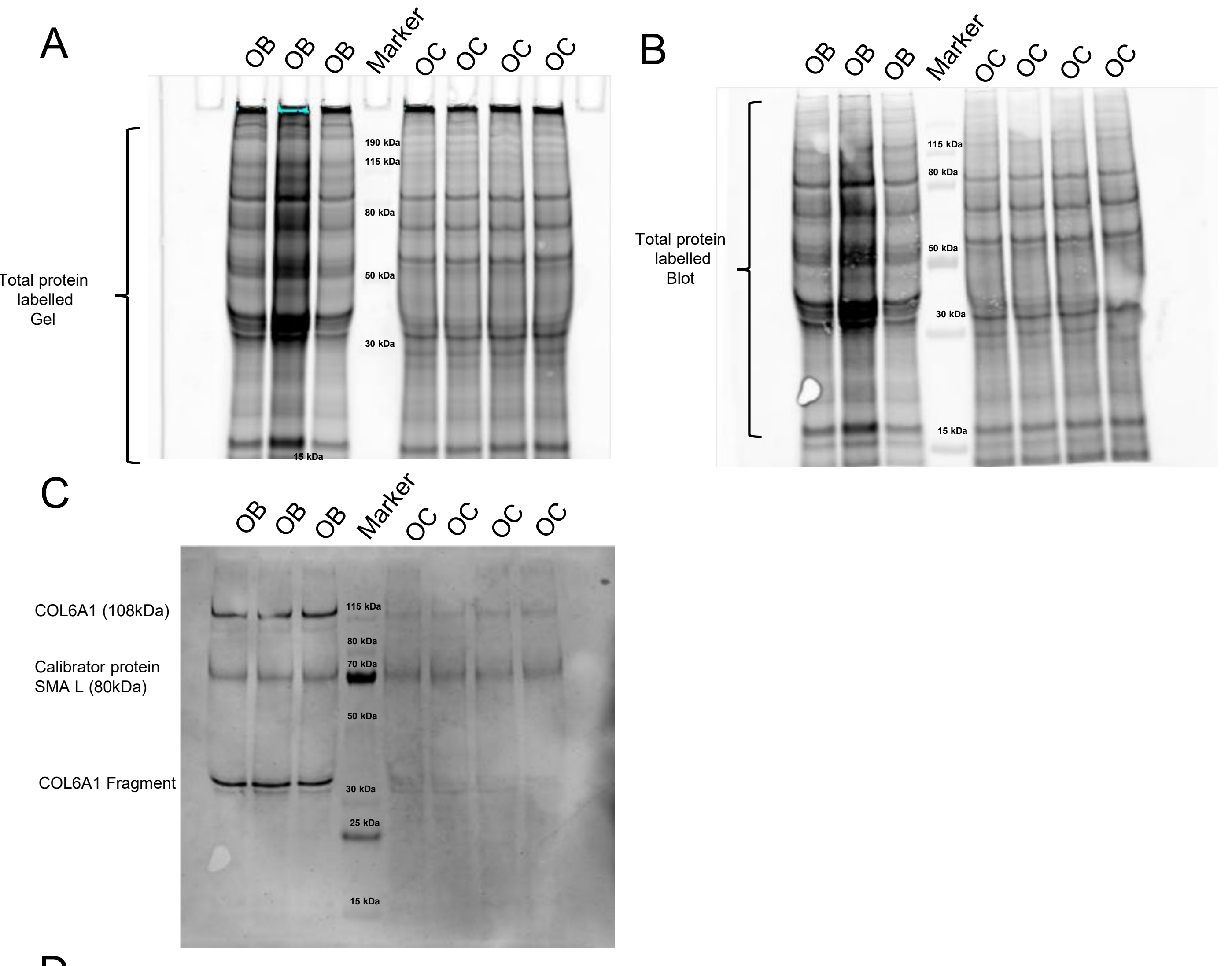

D

Sample	Lane	SPL normalised volume	log2 SPL normalised volume
OB	Lane 1 Band 1	198031	17.60
OB	Lane 2 Band 1	146463	17.16
OB	Lane 3 Band 1	282041	18.11
OC	Lane 4 Band 1	65883	16.01
OC	Lane 5 Band 1	84048	16.36
OC	Lane 6 Band 1	44844	15.45
OC	Lane 7 Band 1	40745	15.31

Av OB SPL norm Vol	Av OC SPL norm Vol	Av OB SPL norm log2 Vol	Av OB SPL norm log2 Vo	p-value	Fold Change
208845	58880	17.62	15.78	0.0079	3.55

Supplementary figure S3: Quantitative Smart Protein Layers (SPL) Western Blot analysis of osteoblast (OB) and osteocyte (OC) cells,. SPL Western Blotting system (NH DyeAGNOSTICS GmbH) was used for the detection of quantitative differences of collagen type VI alpha 1 chain (COL6A1) between OB and OC cells. A) Resulting BisTris Gel and B) corresponding Blot, whereby total protein was pre-labeled with a red fluorescent fluorophore (700nm) and a 80 kDa green fluorescence-labeled standard protein (SMA L, 800 nm) was spiked in, enabling error correction of differing sample loading and data normalization between experiments. A primary antibody against COL6A1 was visualized by infrared secondary antibodies (IRDye goat anti-mouse 800nm, C). D) Quantification of SPL volumes was carried out in the vendor software and a significant enrichment of COL6A1 was verified by Student's t-test p-value (p-value < 0,05). An enrichment of 3.55 was determined by calculating the fold change by dividing the average (av) normalized volume of OB cells by the average normalized volume of OC cells.

AKAP12 (K.SAESPTSPVTSETGSTFK.K [280, 297], 2+)

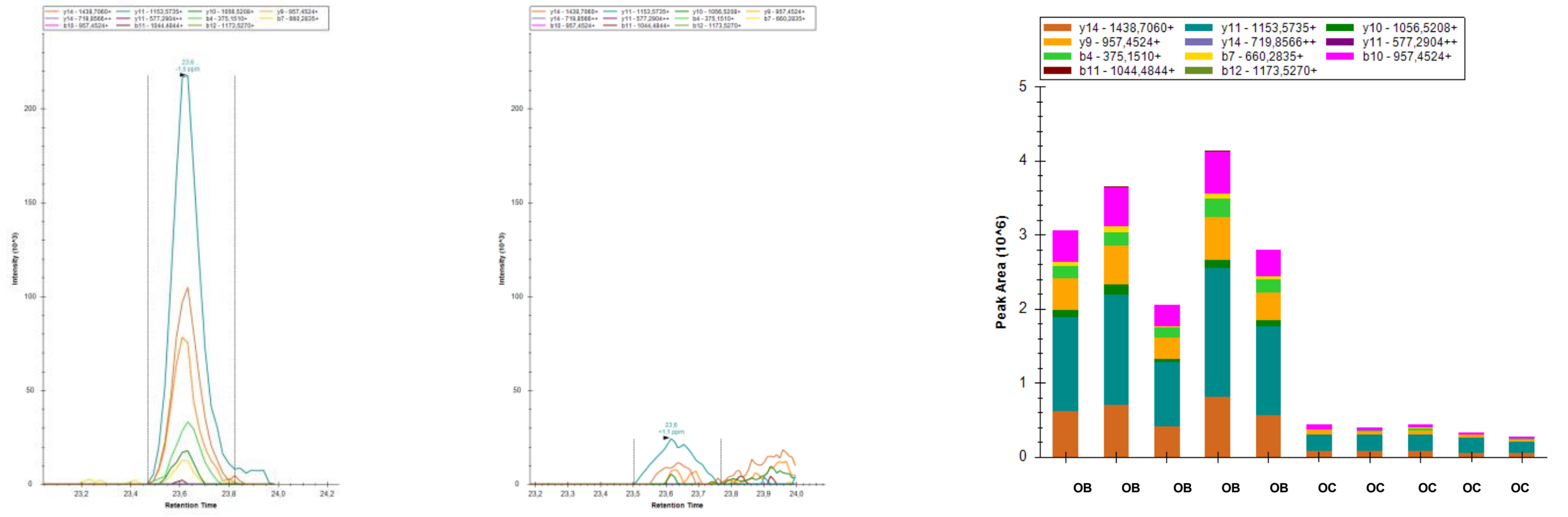

AKAP12 (R.TQEVLQAVAEK.V [1039, 1049], 2+)

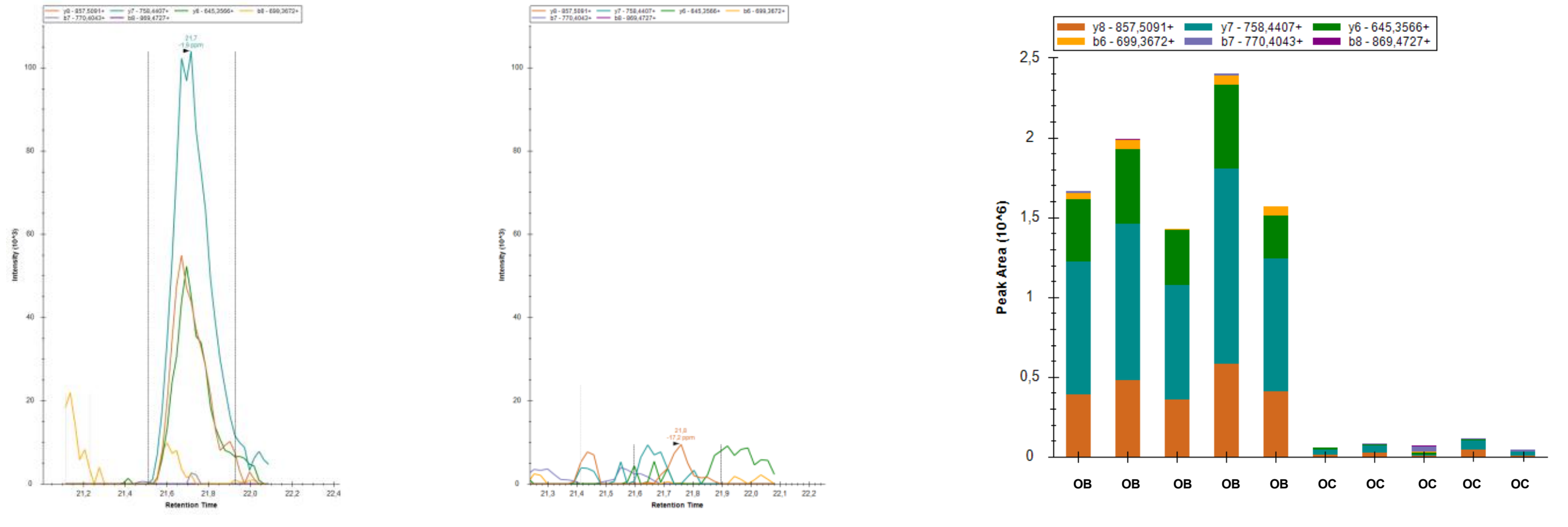

AKAP12 (K.VVGQTTPESFEK.A [1111, 1122], 2+)

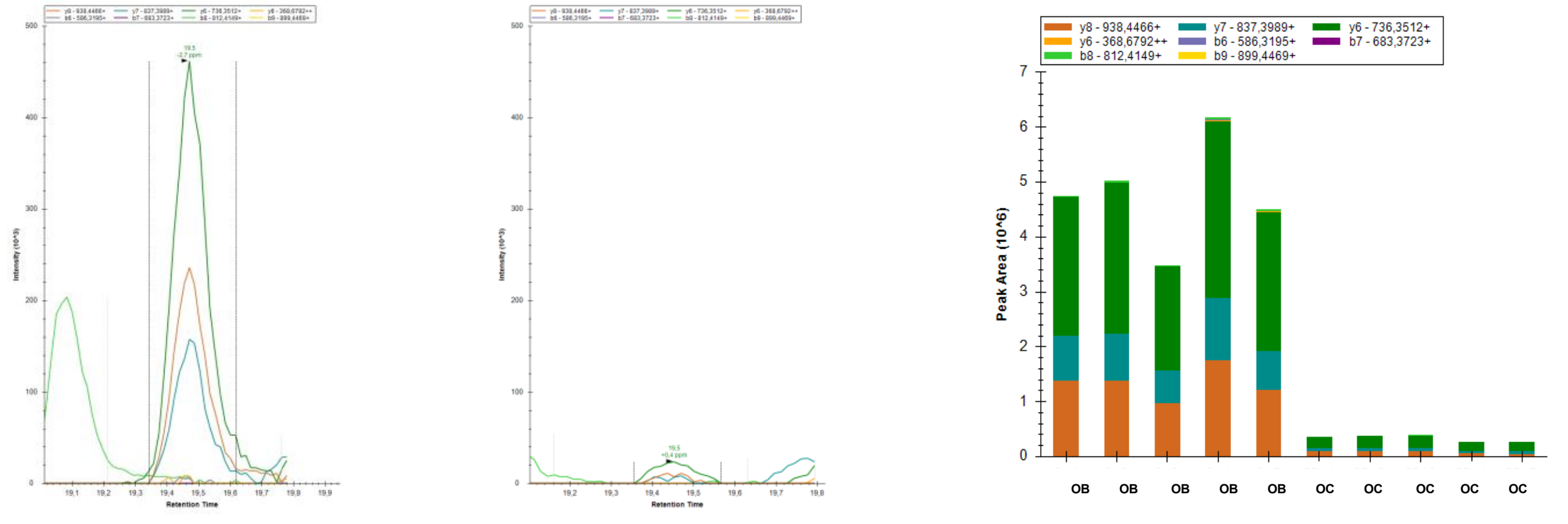

AKAP12 (K.ERPPAPSSFVFQEETK.E [1219, 1234], 3+)

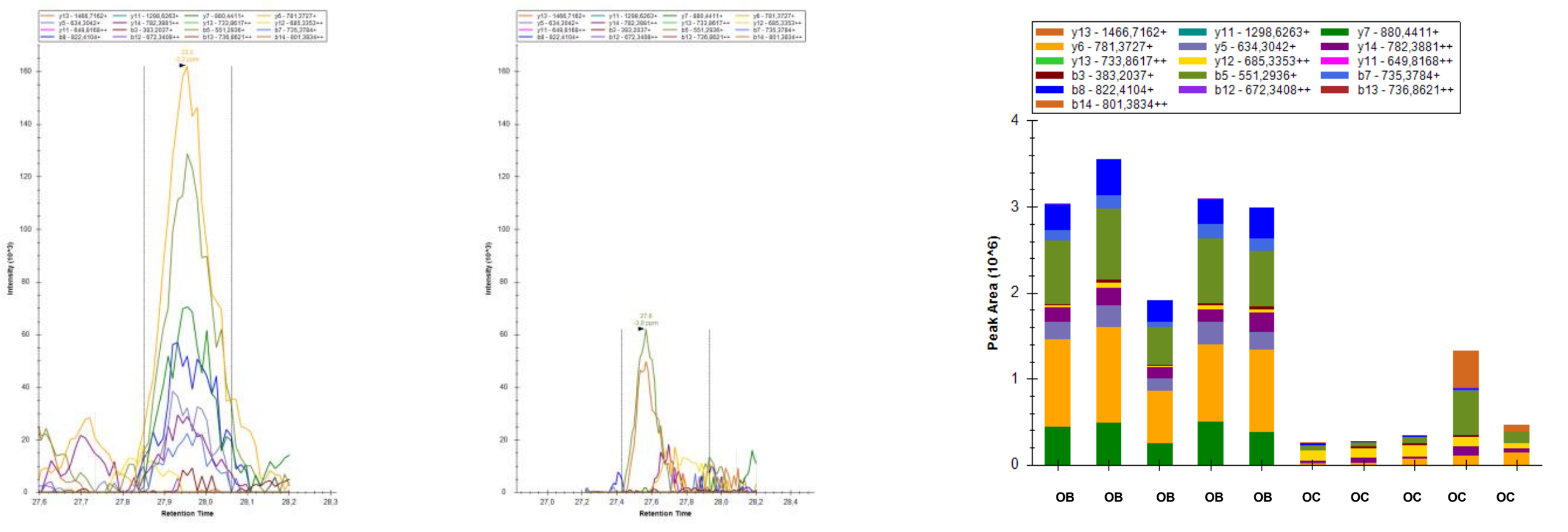

AKAP12 (K.STPVIVSATTK.K [1483, 1493], 2+)

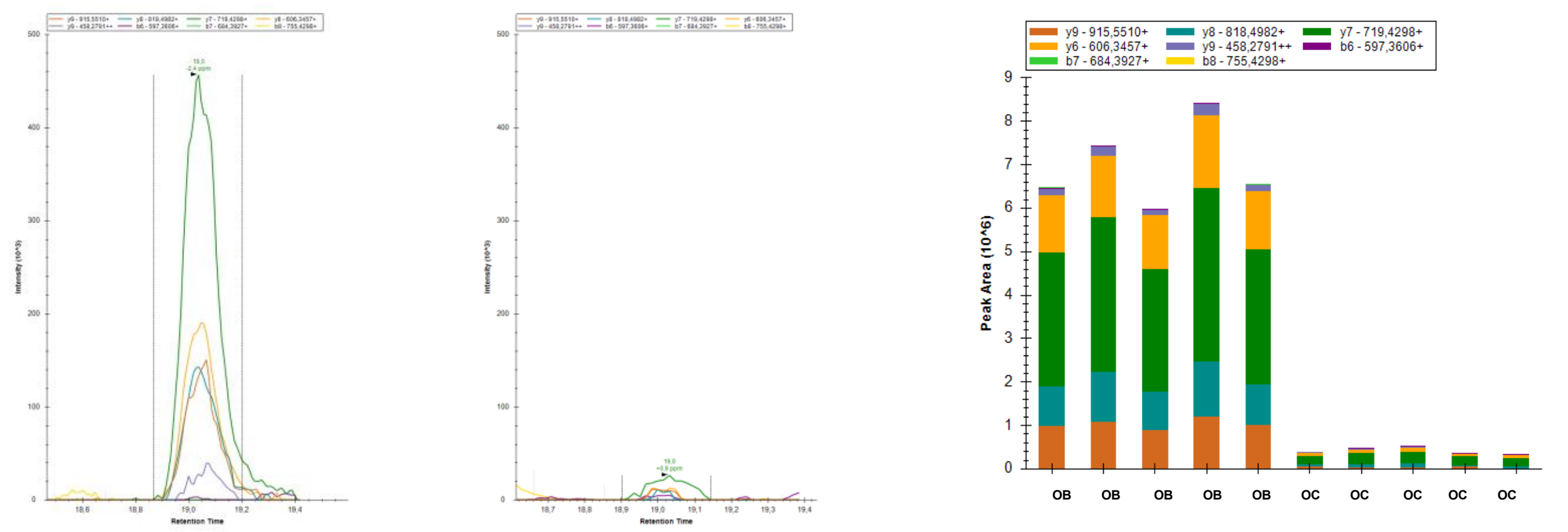

AKAP12 (K.GLSSDLEGEK.T [1495, 1504], 2+)

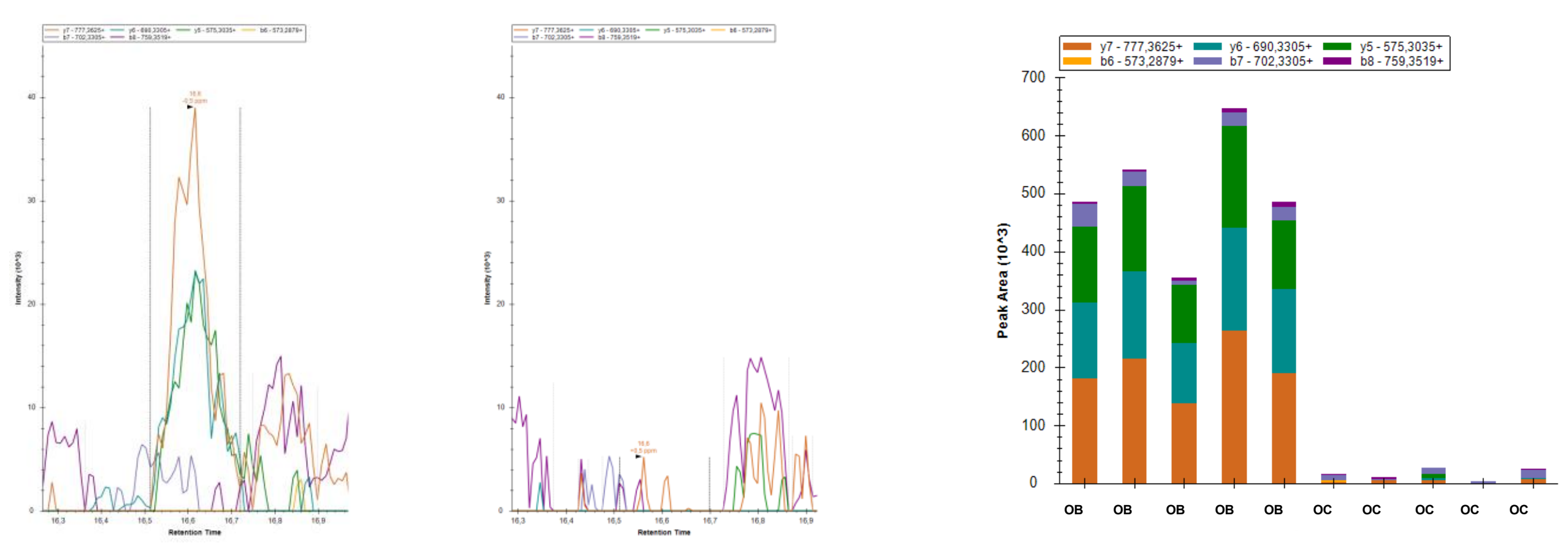

AKAP12 (K.SVPEDDGHALLAER.I [1674, 1687], 2+)

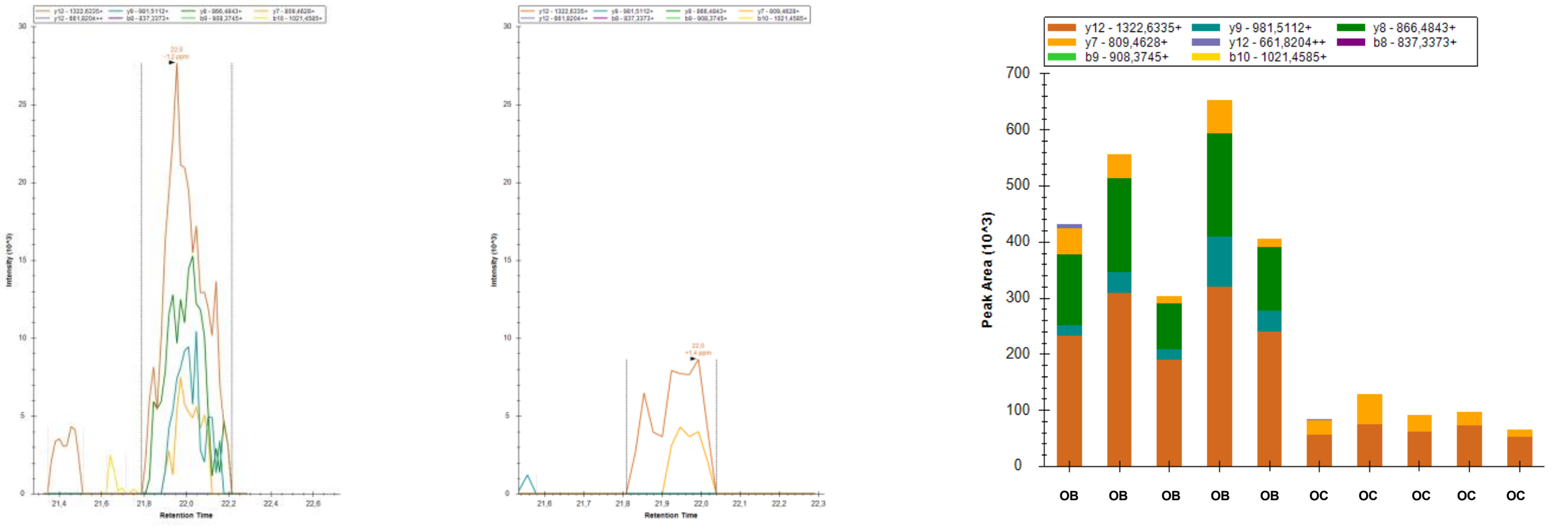

Supplementary figure S4: Representative MS2 Intensities for each peptide included in the paralell reaction monitoring (PRM) measurements for AKAP12. The headder includes information on the peptide sequence, as well as peptide position within the protein, and charge state. For each peptide MS2 signals for osteoblast cells (OB, left) and osteocytes (OC, middle) are shown, as well as stacked bar charts (right) for MS2 fragment ion intensities for each replicate (n=5 per sample type).

BCL9 (R.STAPLNTQISALR.N [216, 228], 2+)

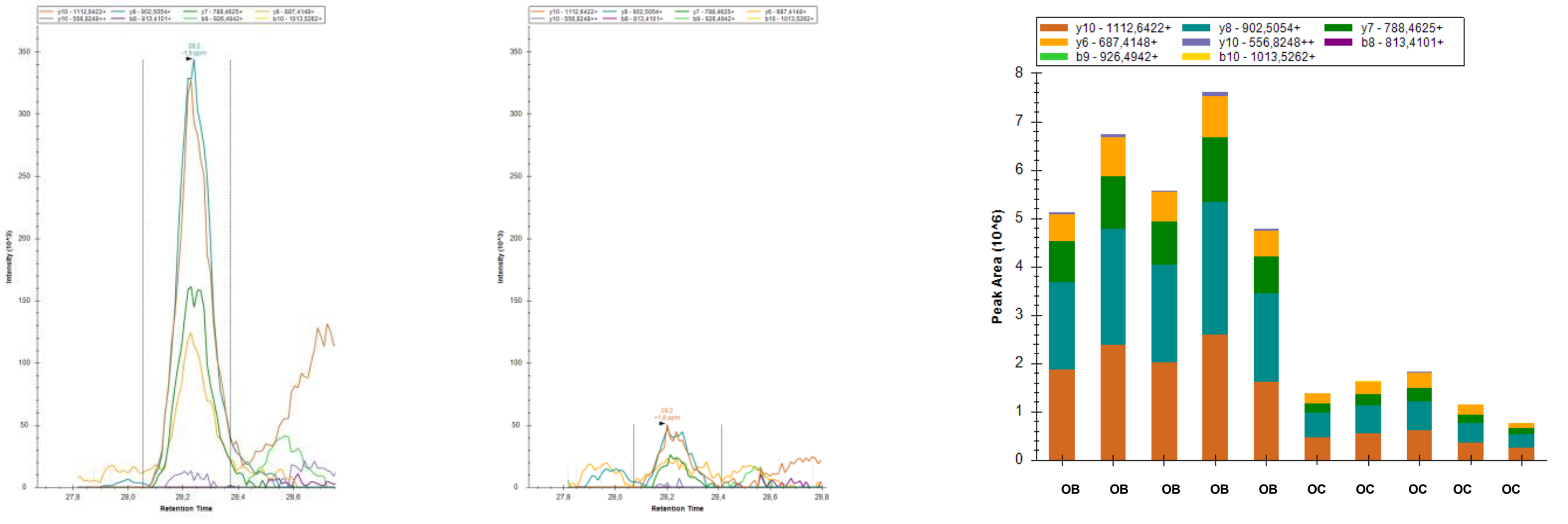

BCL9 (R.IPVEGPLSPSR.G [680, 690], 2+)

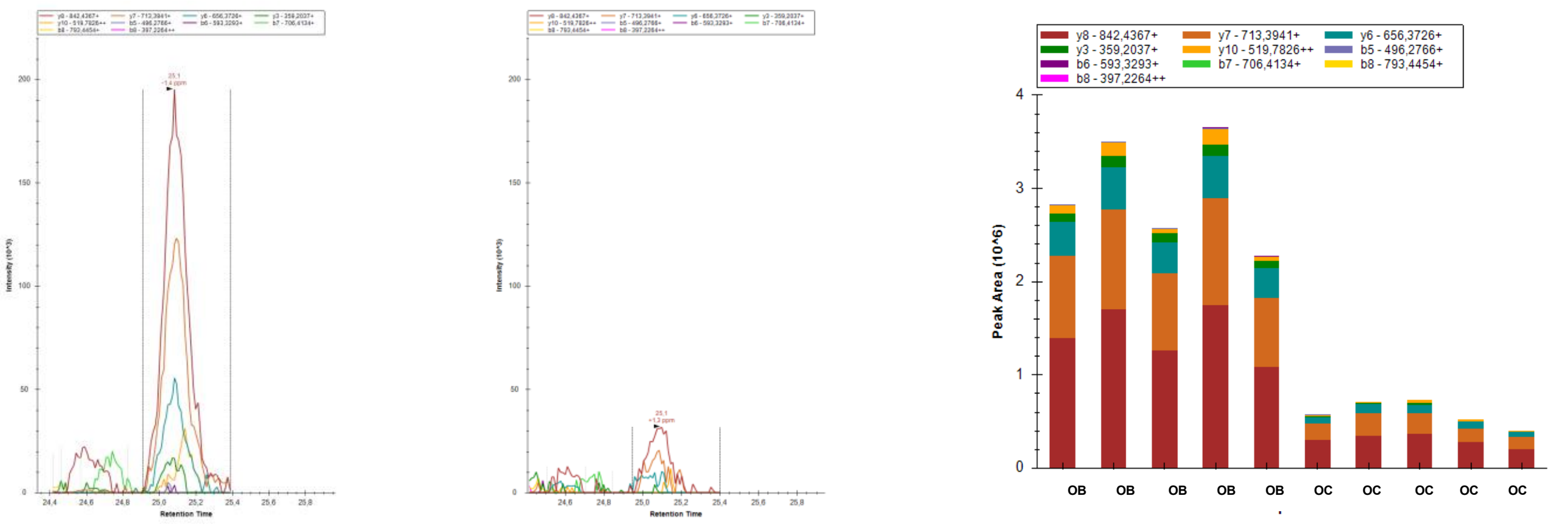

BCL9 (K.LRPGGSDMLPAQQK.M [750, 763], 3+)

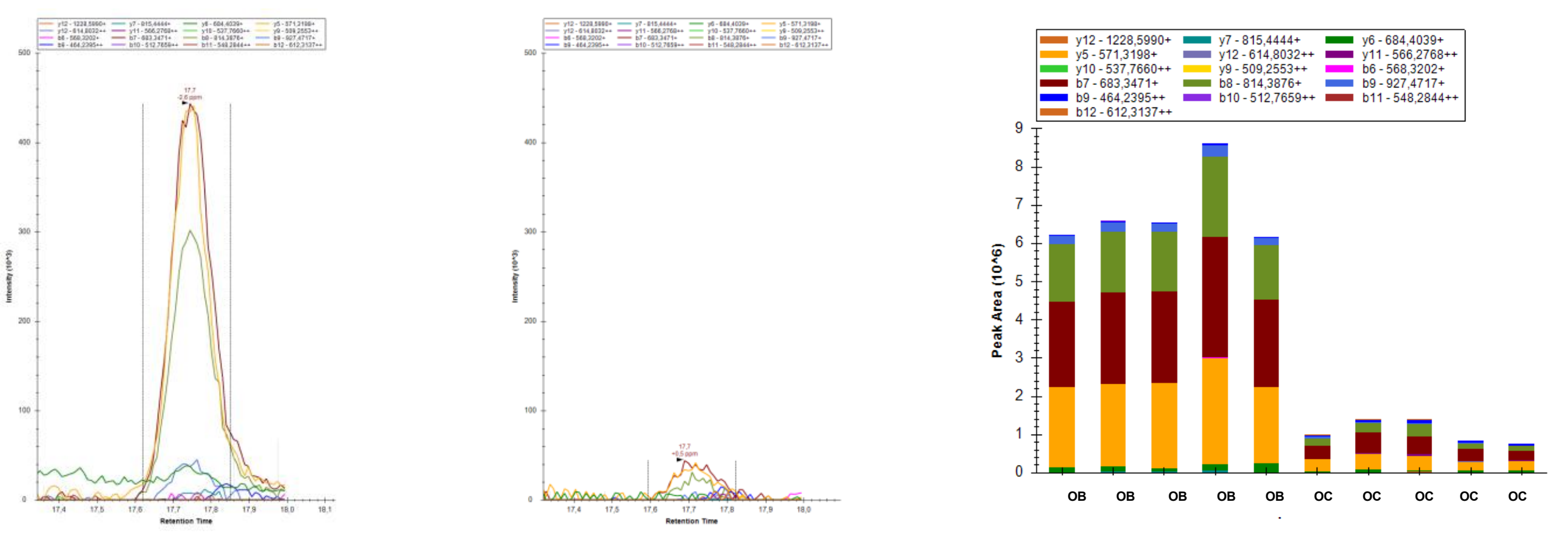

BCL9 (K.SPPVLGSAAASPVHLK.S [907, 922], 3+)

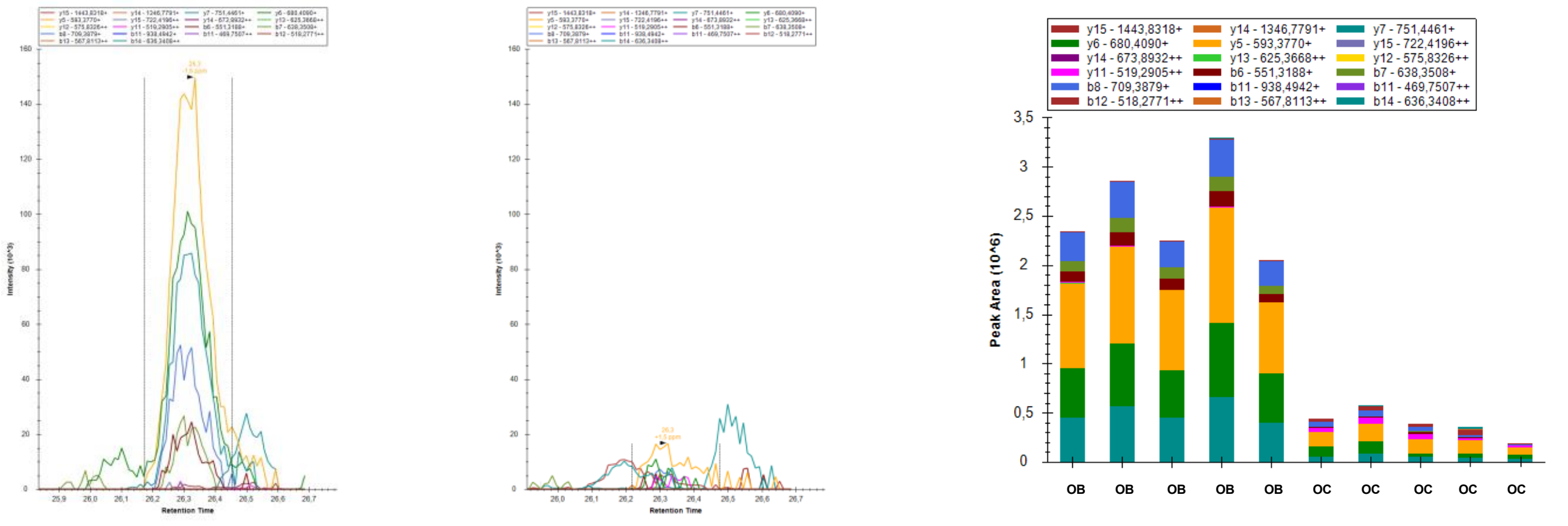

Supplementary figure S5: Representative MS2 Intensities for each peptide included in the paralell reaction monitoring (PRM) measurements for BCL9. The headder includes information on the peptide sequence, as well as peptide position within the protein, and charge state. For each peptide MS2 signals for osteoblast cells (OB, left) and osteocytes (OC, middle) are shown, as well as stacked bar charts (right) for MS2 fragment ion intensities for each replicate (n=5 per sample type).

CAV1 (K.YVDSEGHLYTVPIR.E [6, 19], 3+)

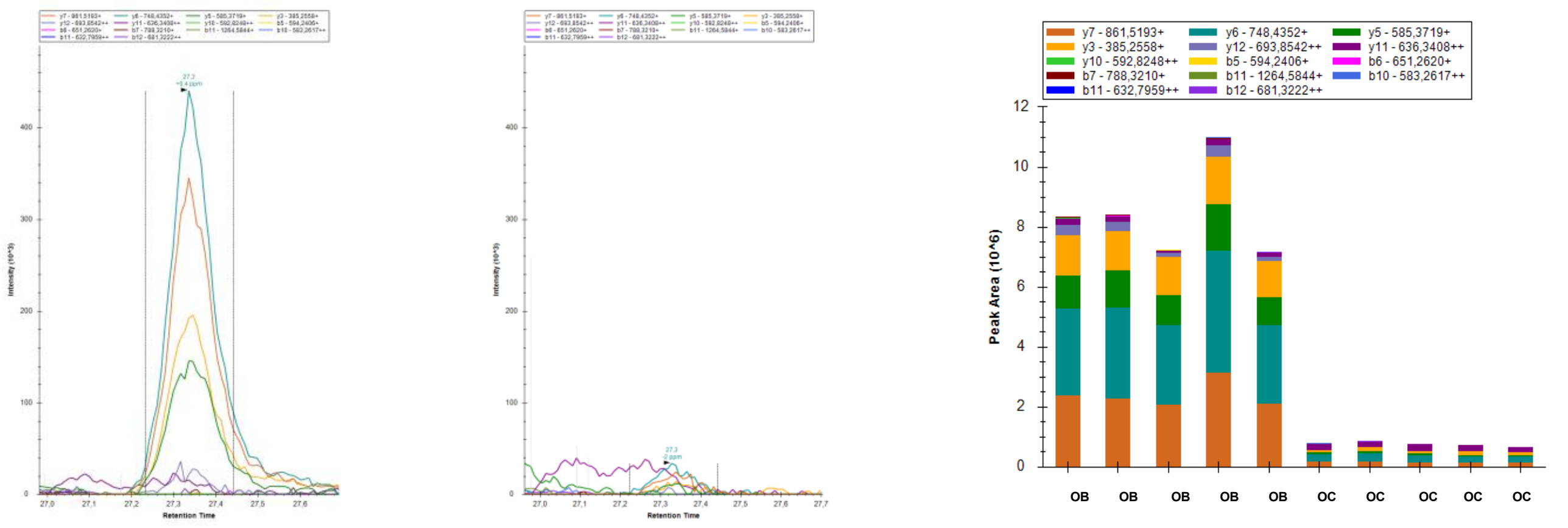

CAV1 (K.AMADELSEK.Q [31, 39], 2+)

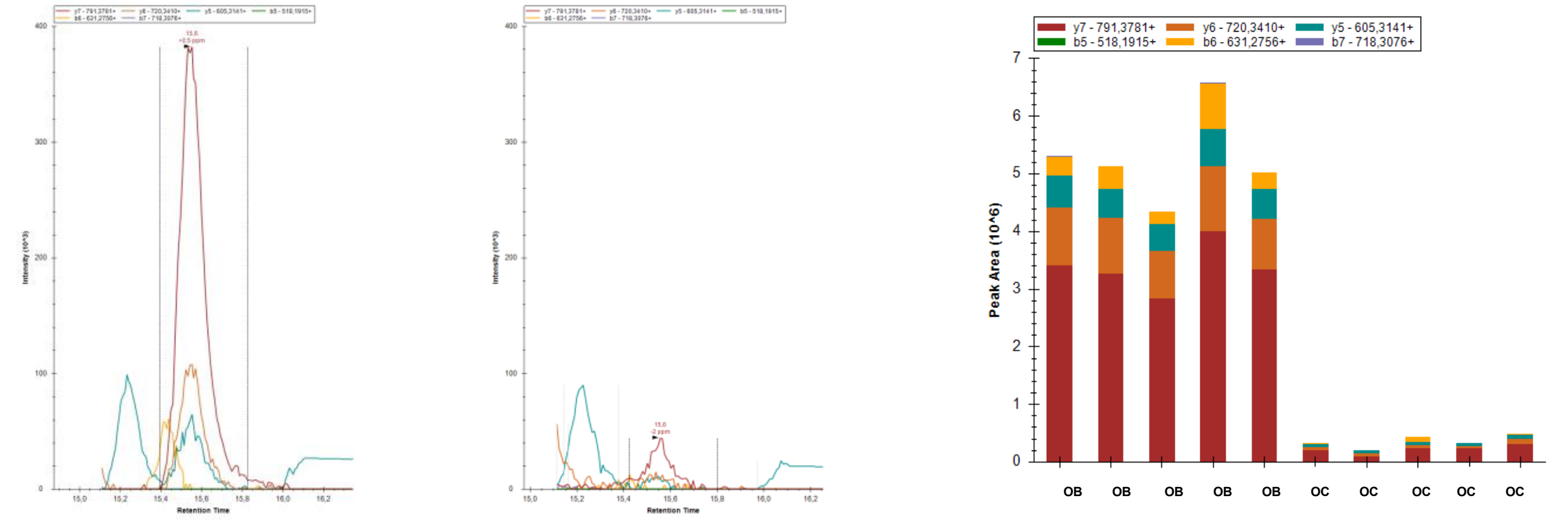

CAV1 (K.HLNDDVVK.I [58, 65], 2+)

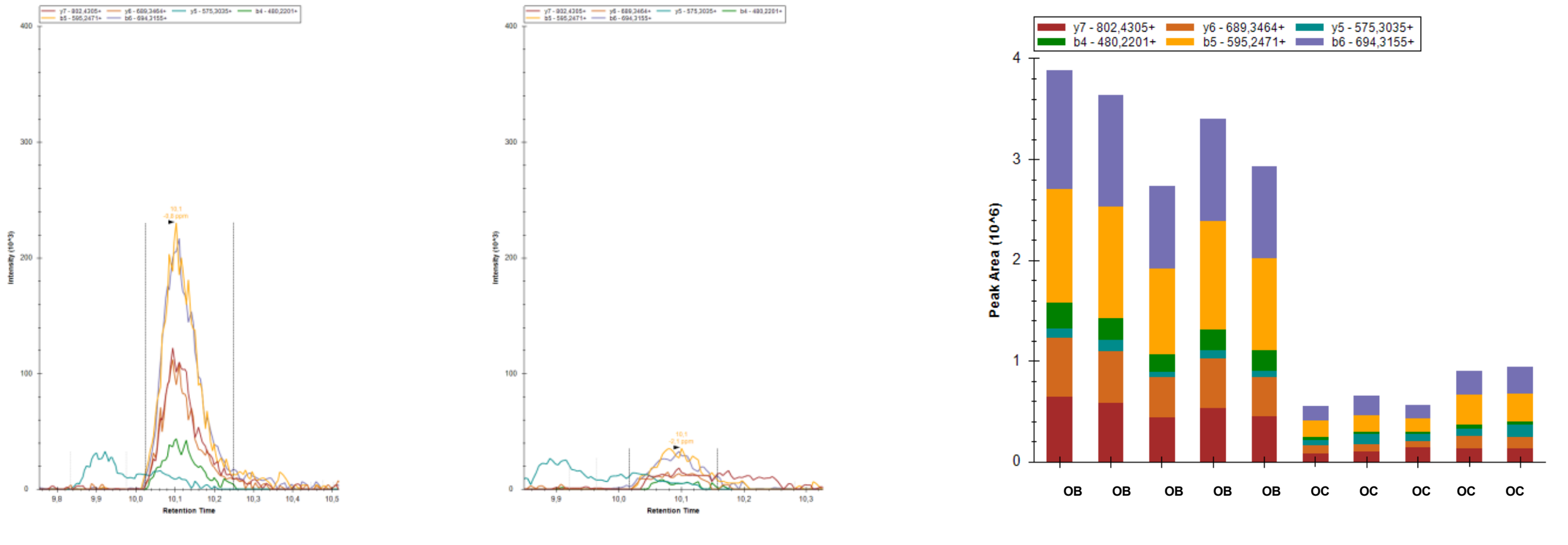

Supplementary figure S6: Representative MS2 Intensities for each peptide included in the paralell reaction monitoring (PRM) measurements for Caveolin-1 (CAV1). The headder includes information on the peptide sequence, as well as peptide position within the protein, and charge state. For each peptide MS2 signals for osteoblast cells (OB, left) and osteocytes (OC, middle) are shown, as well as stacked bar charts (right) for MS2 fragment ion intensities for each replicate (n=5 per sample type).

CCNH (R.FYLNNSVMEYHPR.I [90, 102], 3+)

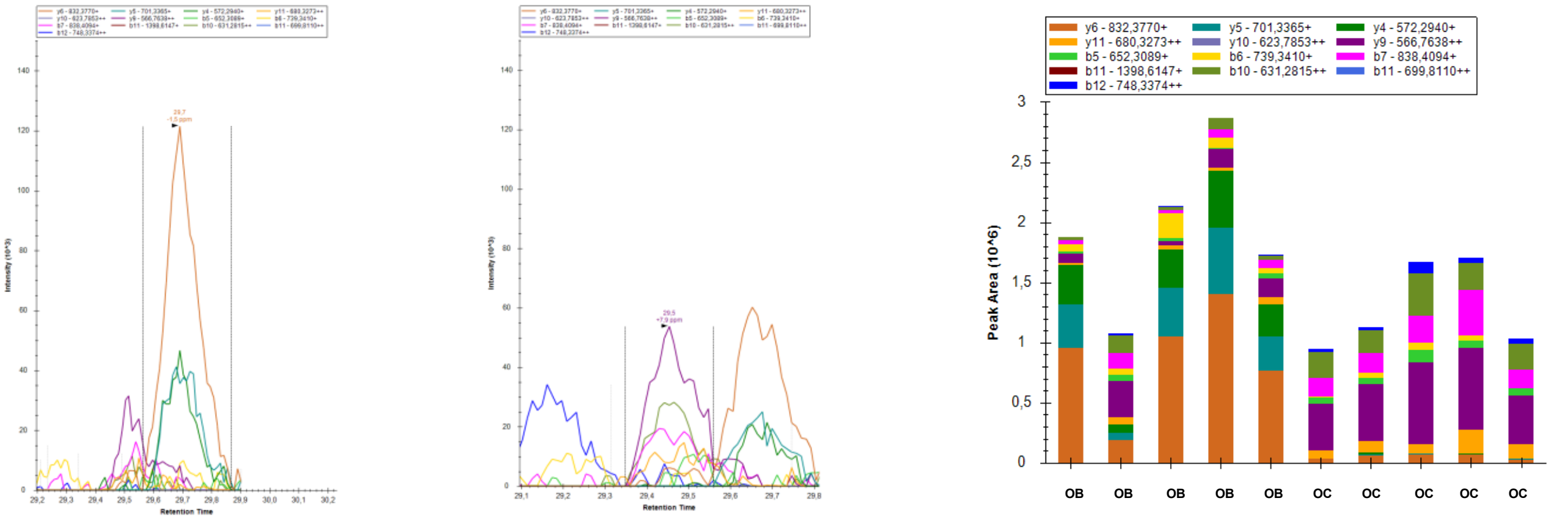

CCNH (K.VDEFNVSSPQFVGNLR.E [115, 130], 3+)

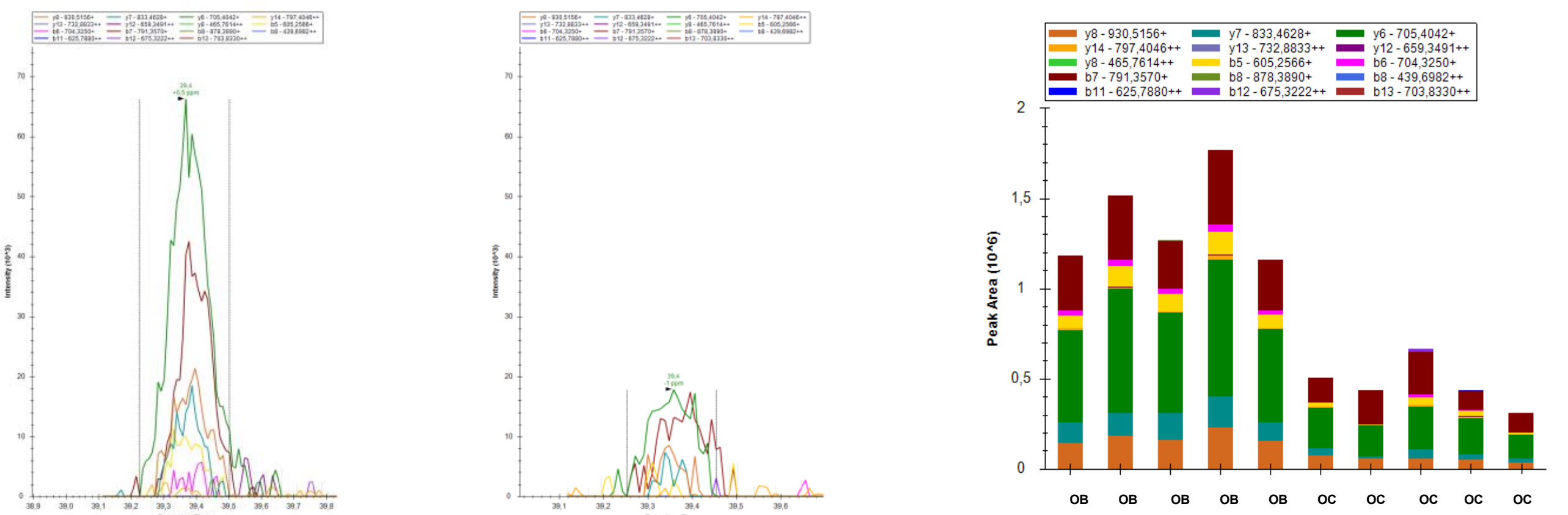

CCNH (R.YPILENPEILR.K [178, 188], 2+)

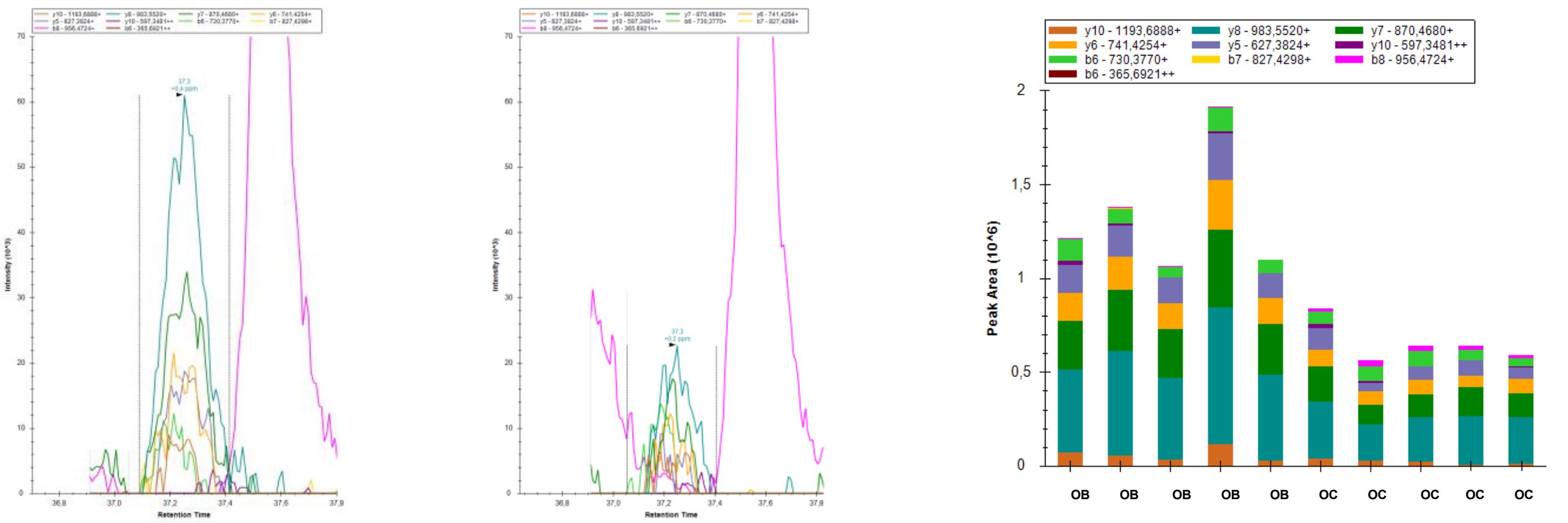

CCNH (K.TADDFLNR.I [190, 197], 2+)

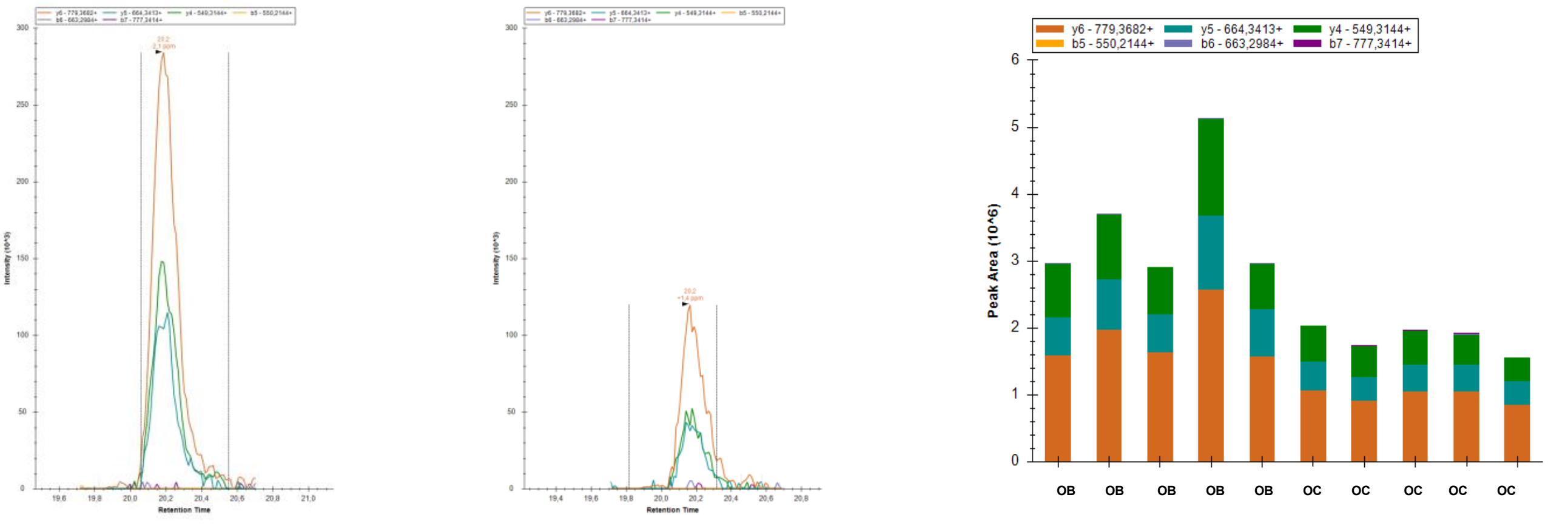

Supplementary figure S7: Representative MS2 Intensities for each peptide included in the parallel reaction monitoring (PRM) measurements for CCNH. The header includes information on the peptide sequence, as well as peptide position within the protein, and charge state. For each peptide MS2 signals for osteoblast cells (OB, left) and osteocytes (OC, middle) are shown, as well as stacked bar charts (right) for MS2 fragment ion intensities for each replicate (n=5 per sample type).

CNN1 (R.GPAYGLSAEVK.N [9, 19], 2+)

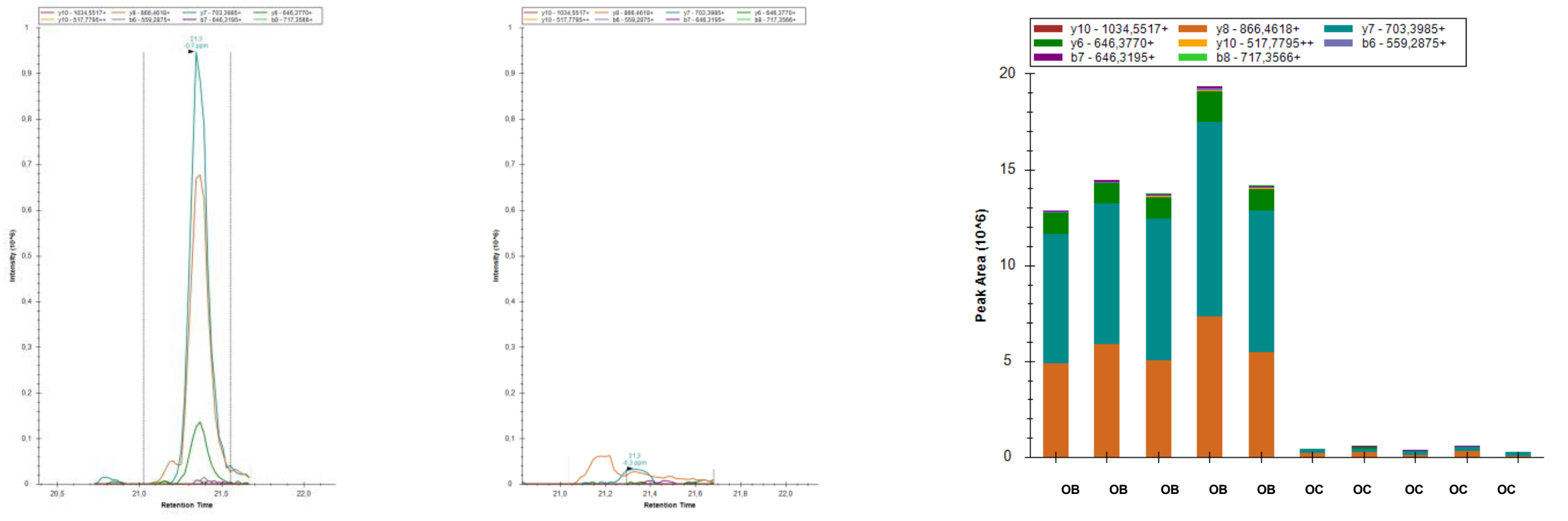

CNN1 (K.GASQAGMTAPGTK.R [213, 225] 2+)

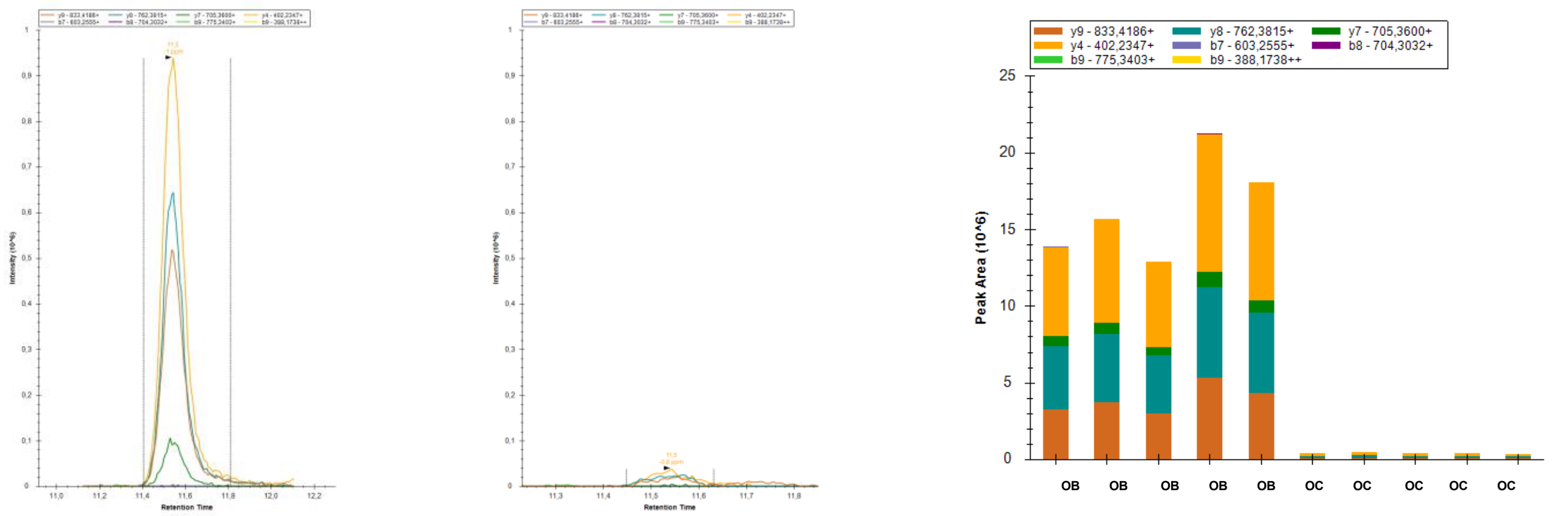

Supplementary figure S8: Representative MS2 Intensities for each peptide included in the parallel reaction monitoring (PRM) measurements for Calponin-1 (CNN1). The header includes information on the peptide sequence, as well as peptide position within the protein, and charge state. For each peptide MS2 signals for osteoblast cells (OB, left) and osteocytes (OC, middle) are shown, as well as stacked bar charts (right) for MS2 fragment ion intensities for each replicate (n=5 per sample type).

MO4 L2 (K.NLEPALPGR.W [50, 58], 2+)

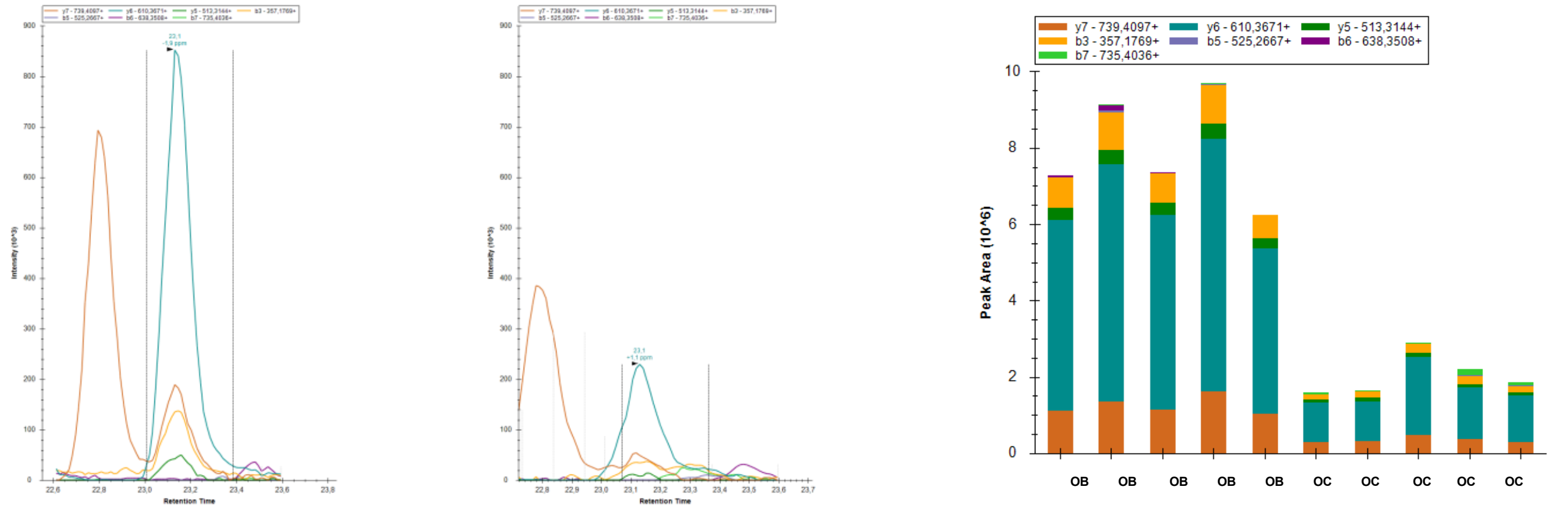

MO4L2 (R.SAENPPSGSVR.K [63, 73], 2+)

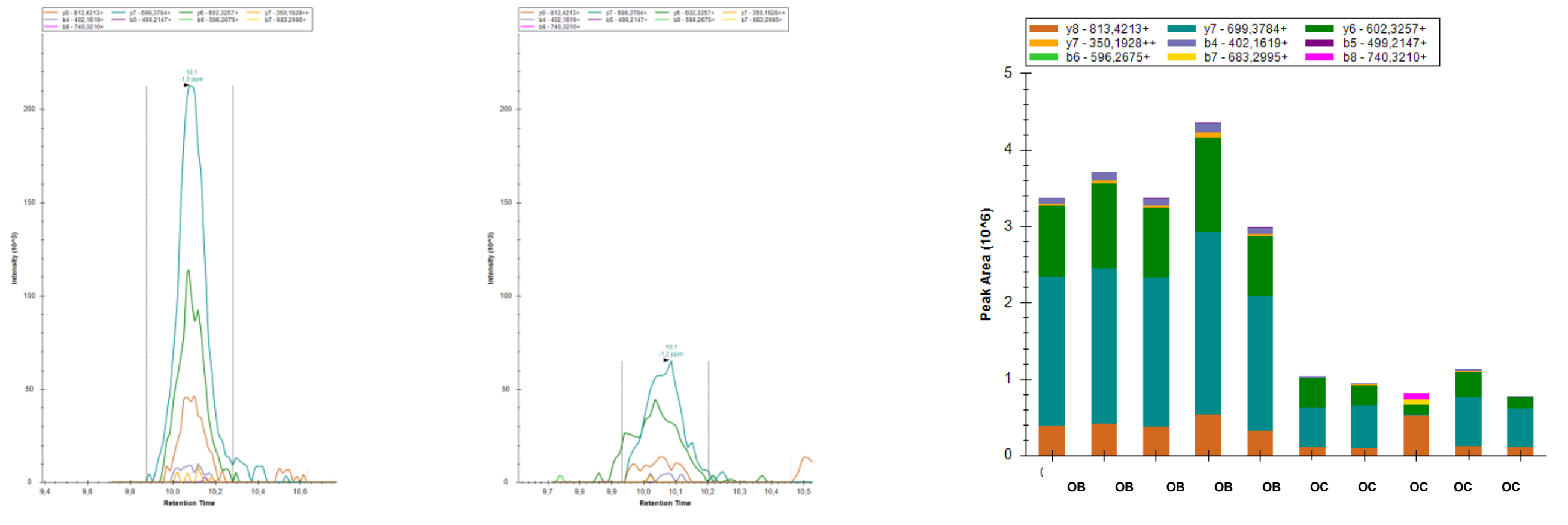

MO4L2 (R.ADPTVESEEAFK.N [105, 116], 2+)

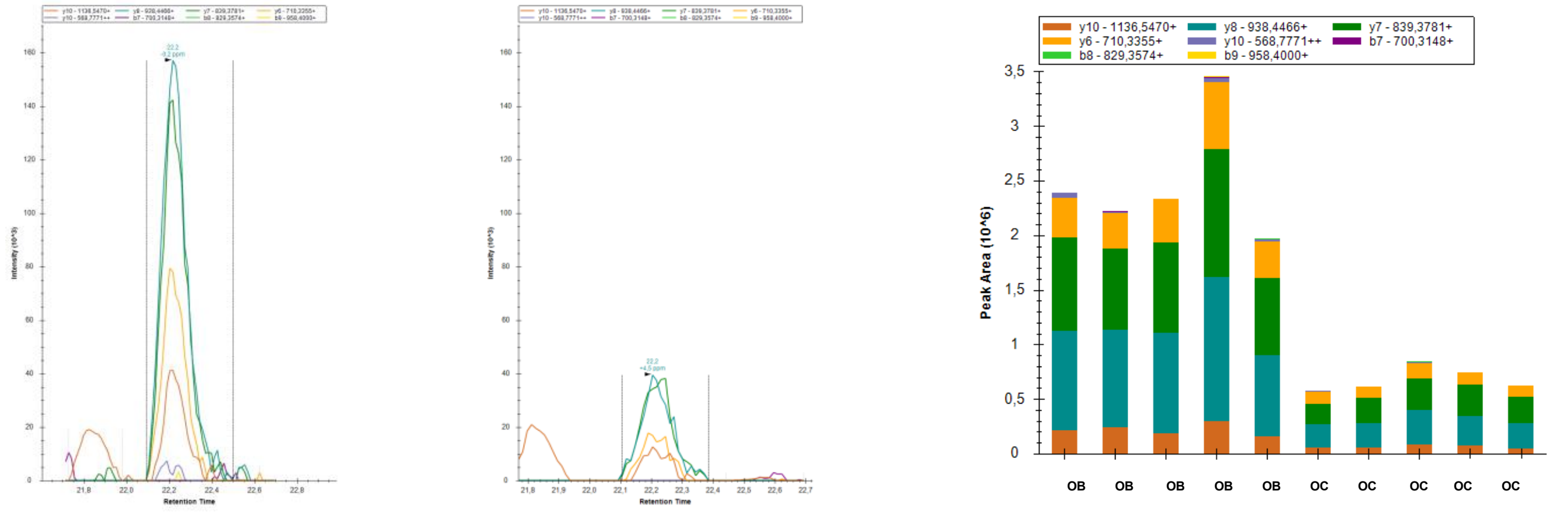

Supplementary figure S9: Representative MS2 Intensities for each peptide included in the parallel reaction monitoring (PRM) measurements for MO4L2. The header includes information on the peptide sequence, as well as peptide position within the protein, and charge state. For each peptide MS2 signals for osteoblast cells (OB, left) and osteocytes (OC, middle) are shown, as well as stacked bar charts (right) for MS2 fragment ion intensities for each replicate (n=5 per sample type).

PDLI 3 (M.PQTVILPGPAPWGFR.L [2, 16], 3+)

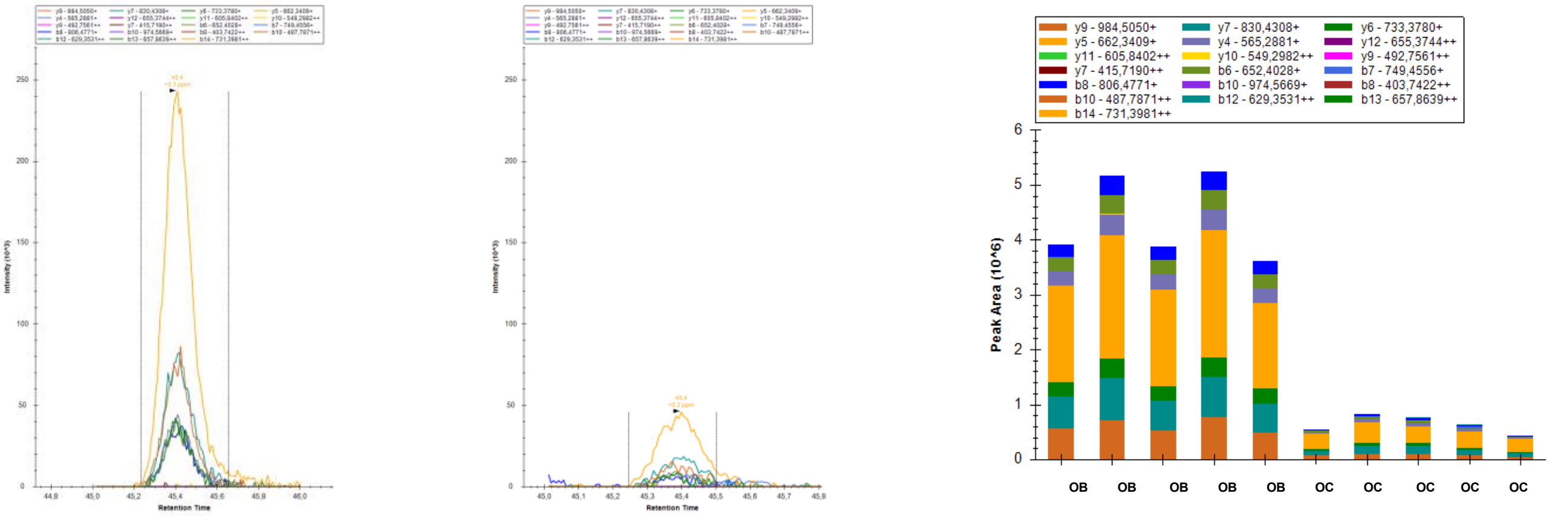

PDLI 3 (R.LSGGIDFNQPLVITR.I [17, 31], 2+)

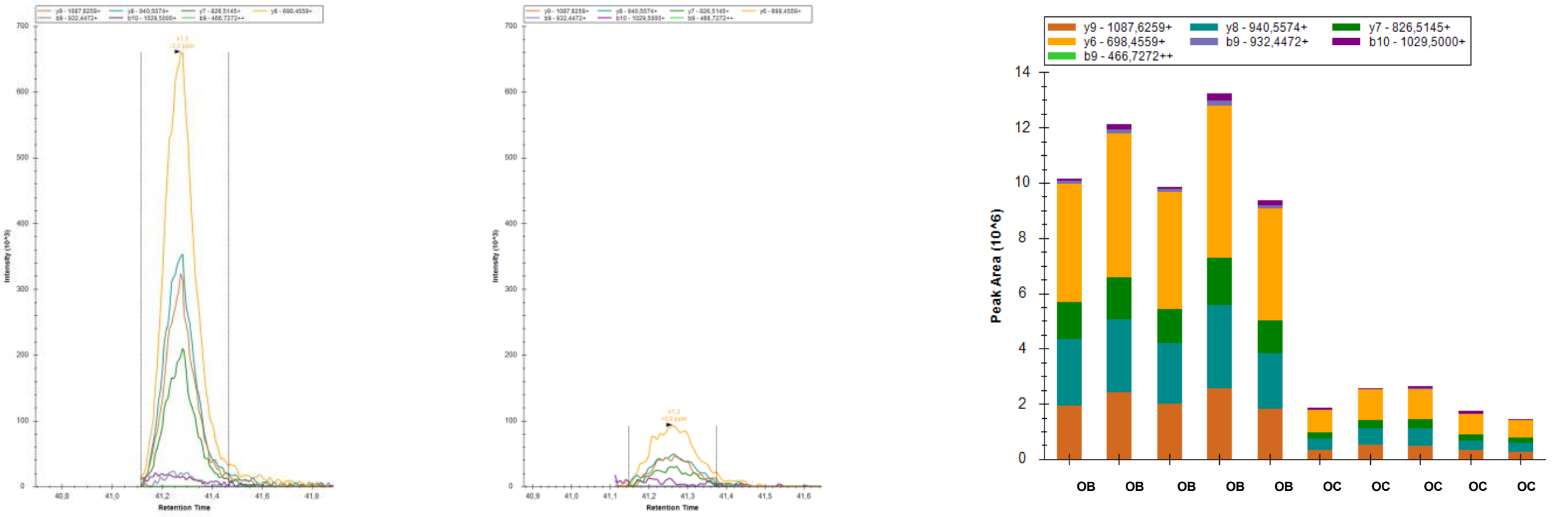

PDLI 3 (R.VLQGMVDDGSDDRPAGTR.S [255, 272], 3+)

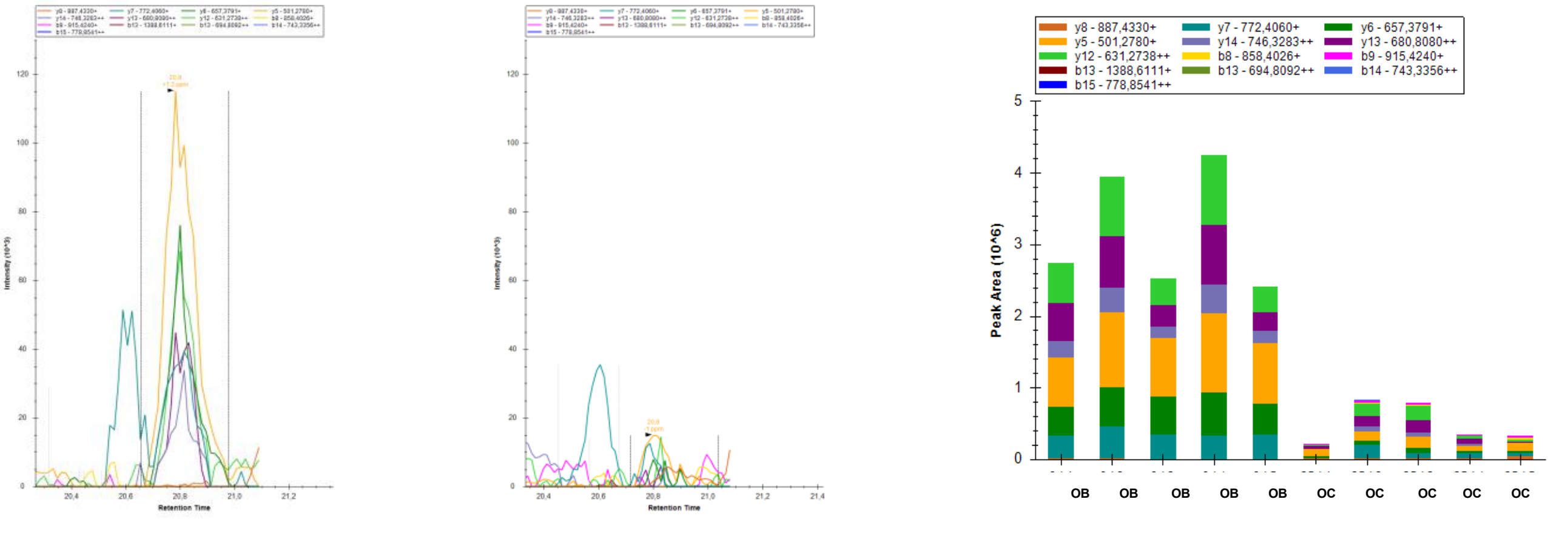

ZN629 (K.SFTQSSHLVQHQR.T [186, 198], 3+)

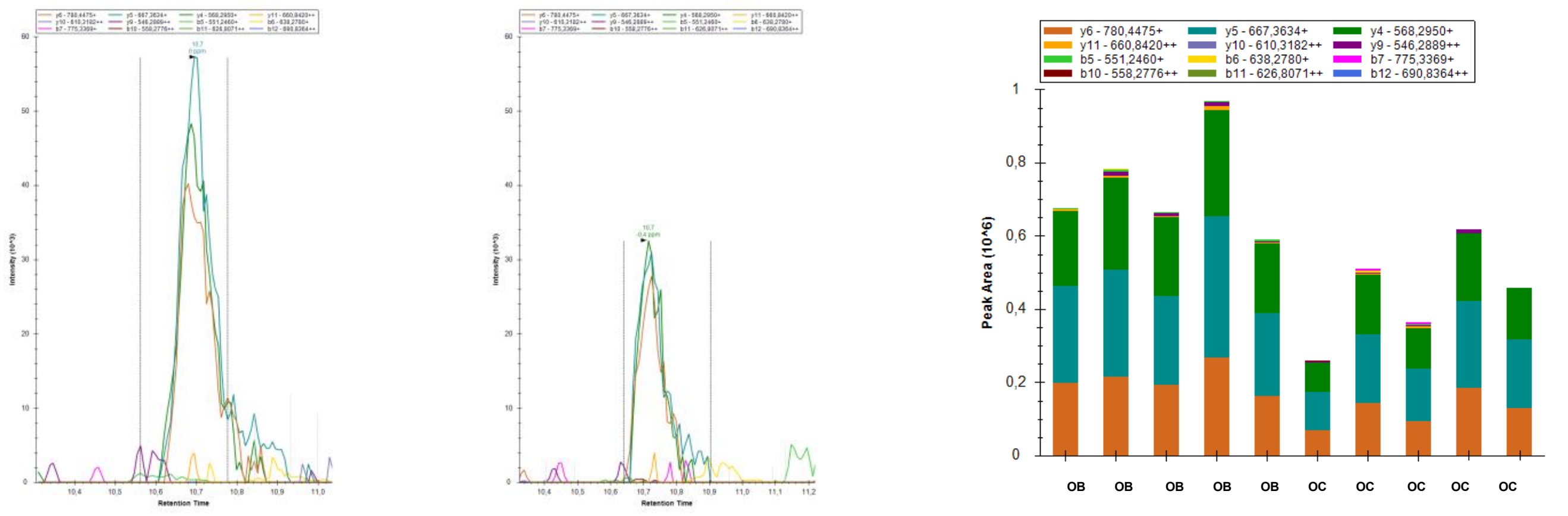

ZN629 (K.SFIQSSELTQHQR.T [326, 338], 3+)

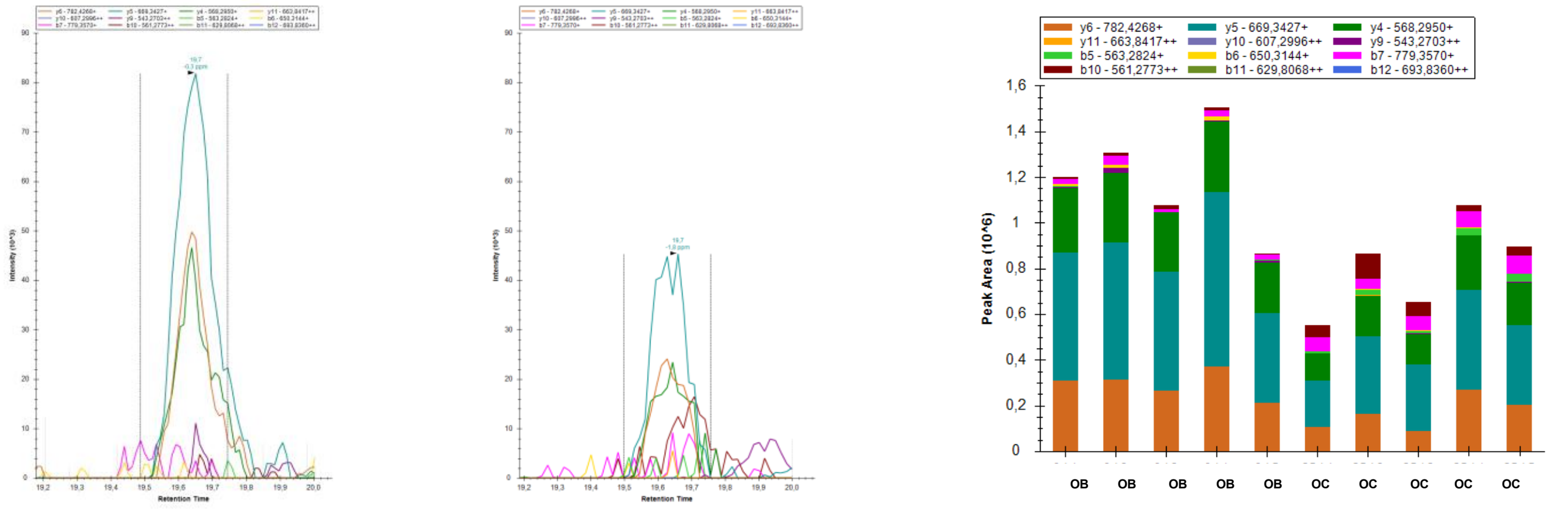

Supplementary figure S10: Representative MS2 Intensities for each peptide included in the parallel reaction monitoring (PRM) measurements for ZN629. The header includes information on the peptide sequence, as well as peptide position within the protein, and charge state. For each peptide MS2 signals for osteoblast cells (OB, left) and osteocytes (OC, middle) are shown, as well as stacked bar charts (right) for MS2 fragment ion intensities for each replicate (n=5 per sample type).

ALDH1L2 (R.HGSIIYHPSLLPR.H [100, 112], 3+)

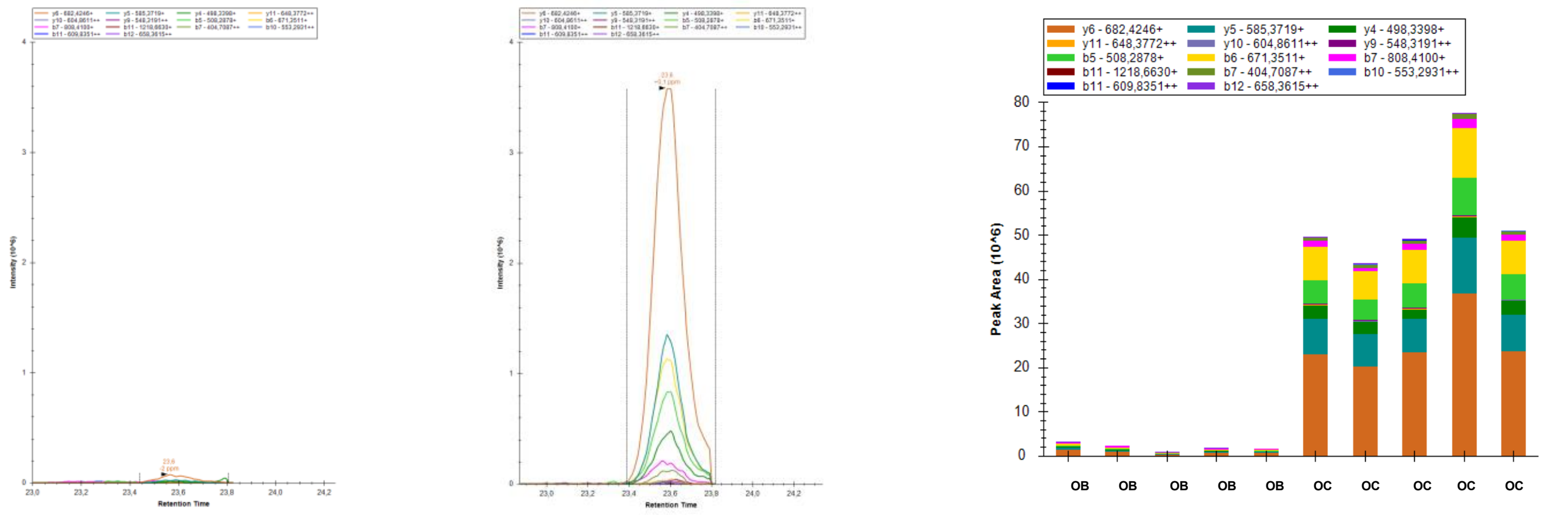

ALDH1L2 (R.LPQPEEGATYEGIQK.K [191, 205], 2+)

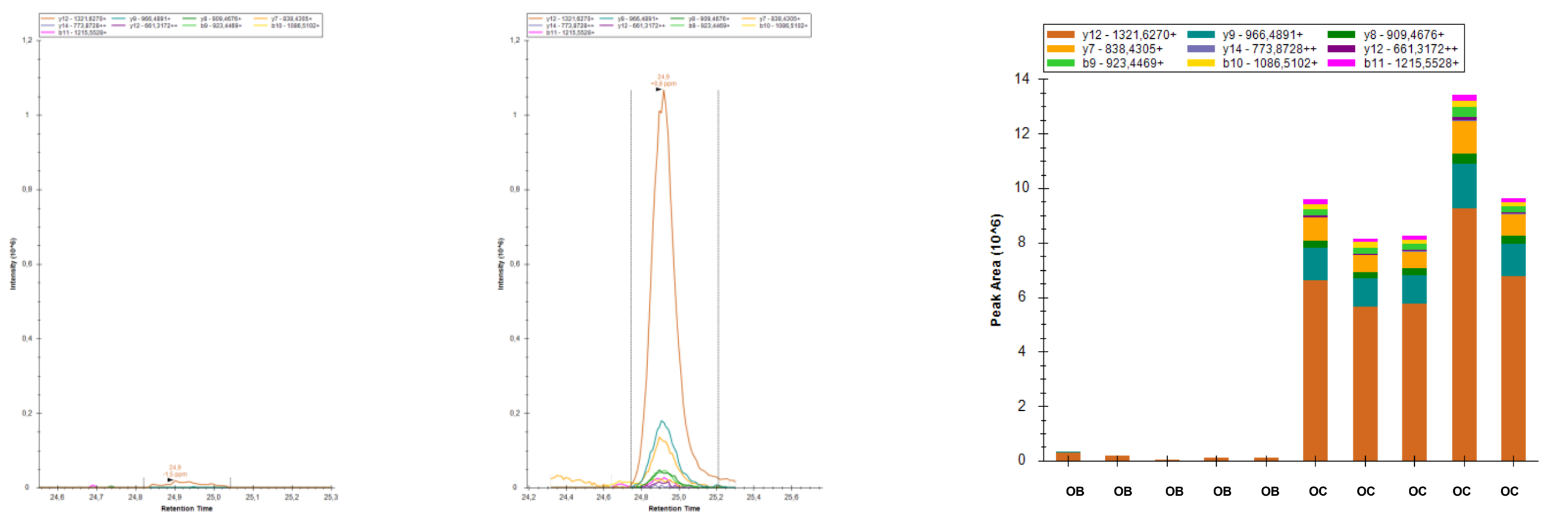

Supplementary figure S11: Representative MS2 Intensities for each peptide included in the parallel reaction monitoring (PRM) measurements for ALDH1L2. The header includes information on the peptide sequence, as well as peptide position within the protein, and charge state. For each peptide MS2 signals for osteoblast cells (OB, left) and osteocytes (OC, middle) are shown, as well as stacked bar charts (right) for MS2 fragment ion intensities for each replicate (n=5 per sample type).

COL1A1 (K.VLSEDTFTEGK.S [278, 288], 2+)

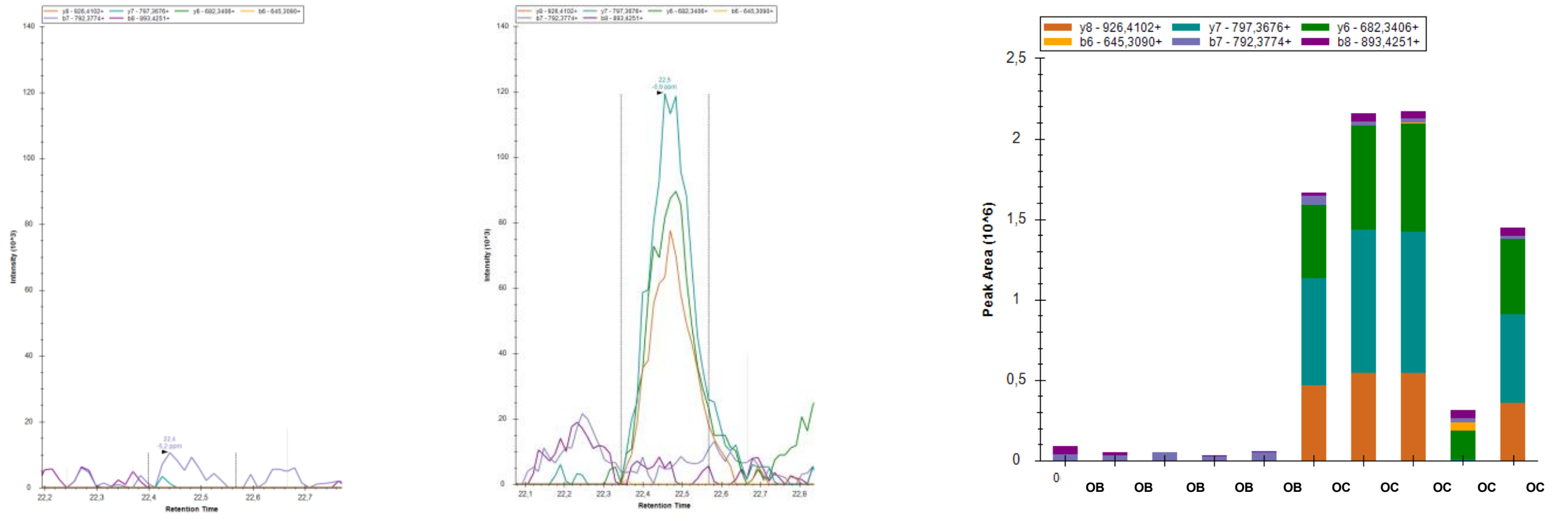

COL1A1 (R.VTGLSLFK.K [384, 391], 2+)

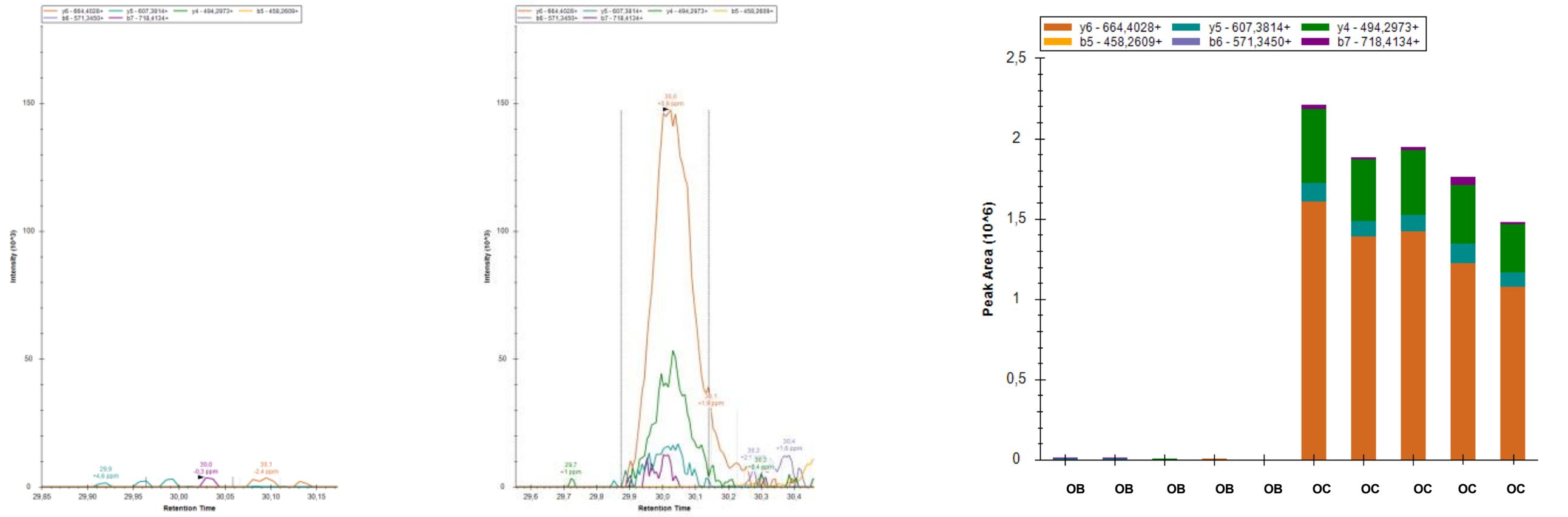

Supplementary figure S12: Representative MS2 Intensities for each peptide included in the parallel reaction monitoring (PRM) measurements for COL1A1. The header includes information on the peptide sequence, as well as peptide position within the protein, and charge state. For each peptide MS2 signals for osteoblast cells (OB, left) and osteocytes (OC, middle) are shown, as well as stacked bar charts (right) for MS2 fragment ion intensities for each replicate (n=5 per sample type).

CRISPLD1 (R.AITDNDMQSILDLHNK.L [56, 71], 3+)

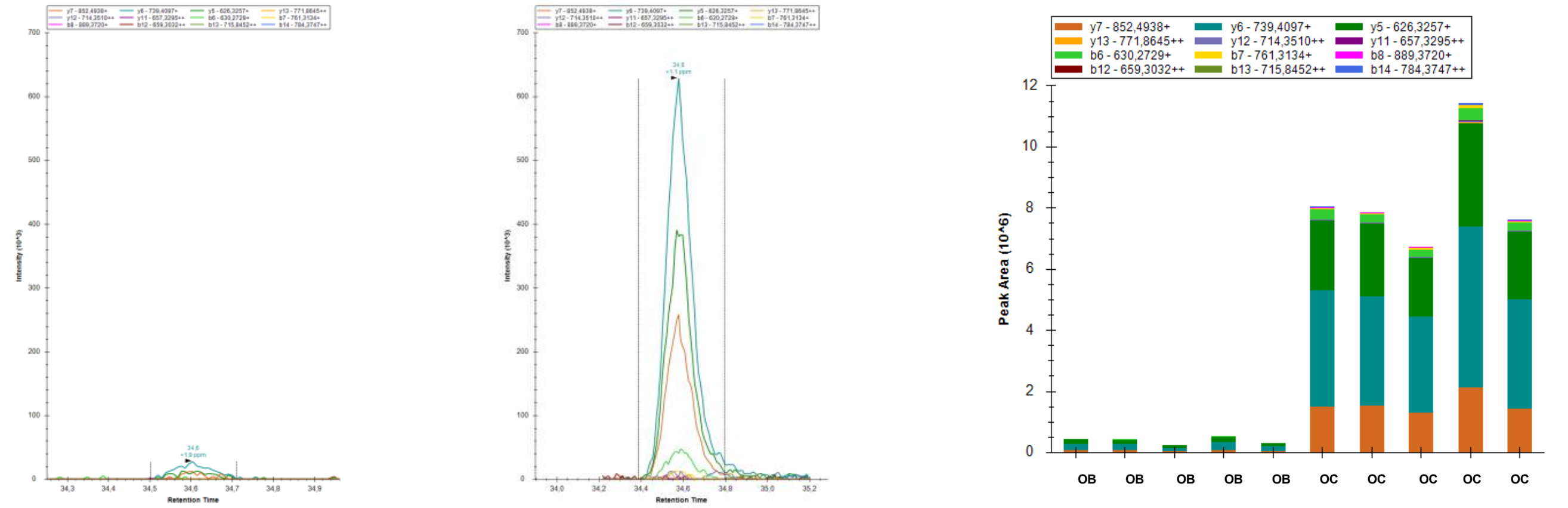

CRISPLD1 (R.AAIHYGIIDNDGGWVDITR.Q [339, 357], 3+)

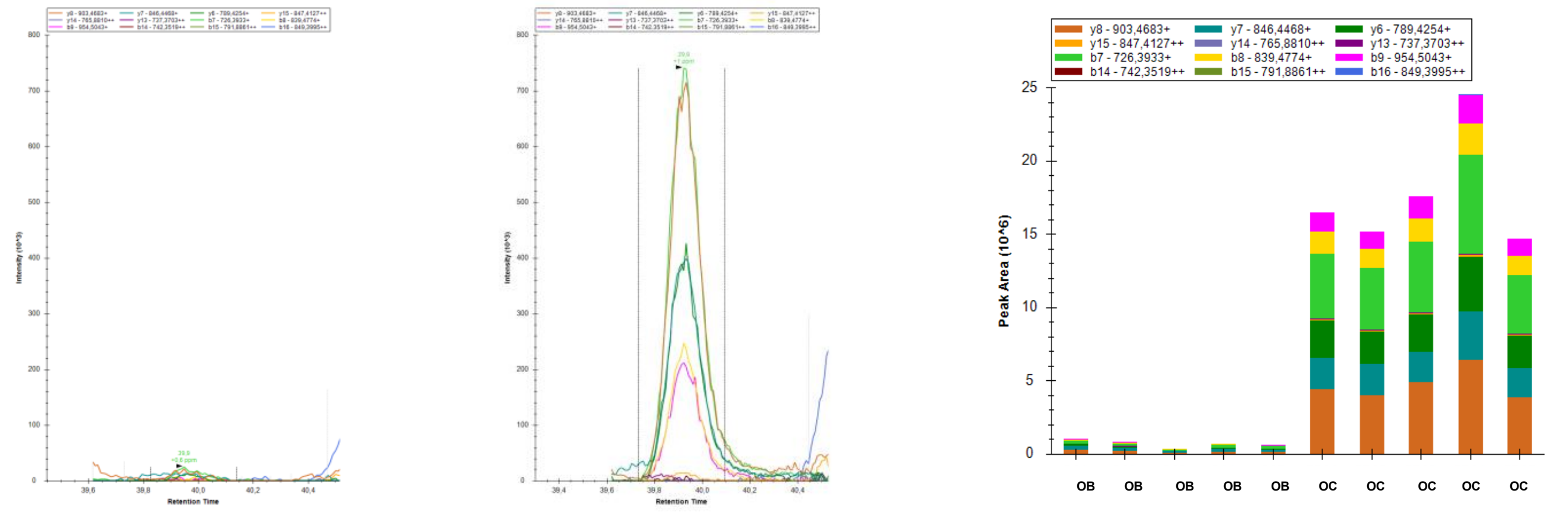

CRISPLD1 (K.YQSANSFTVSK.V [378, 388], 2+)

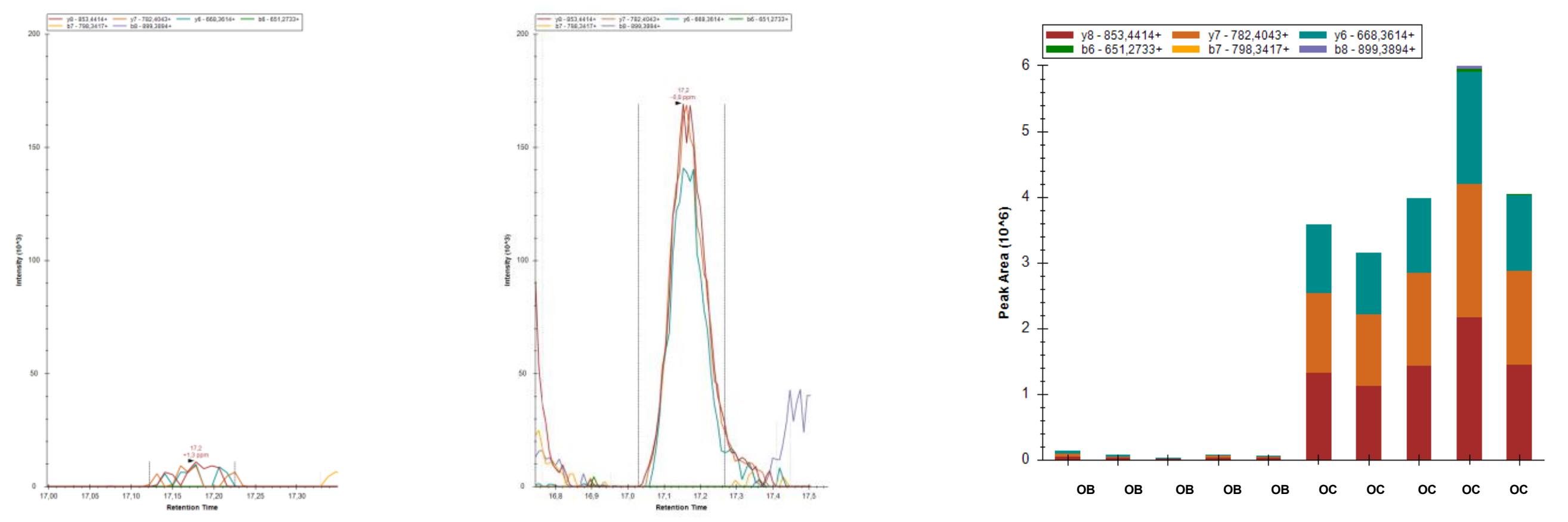

CRISPLD1 (R.AAVHAGVVR.N [447, 455], 2+)

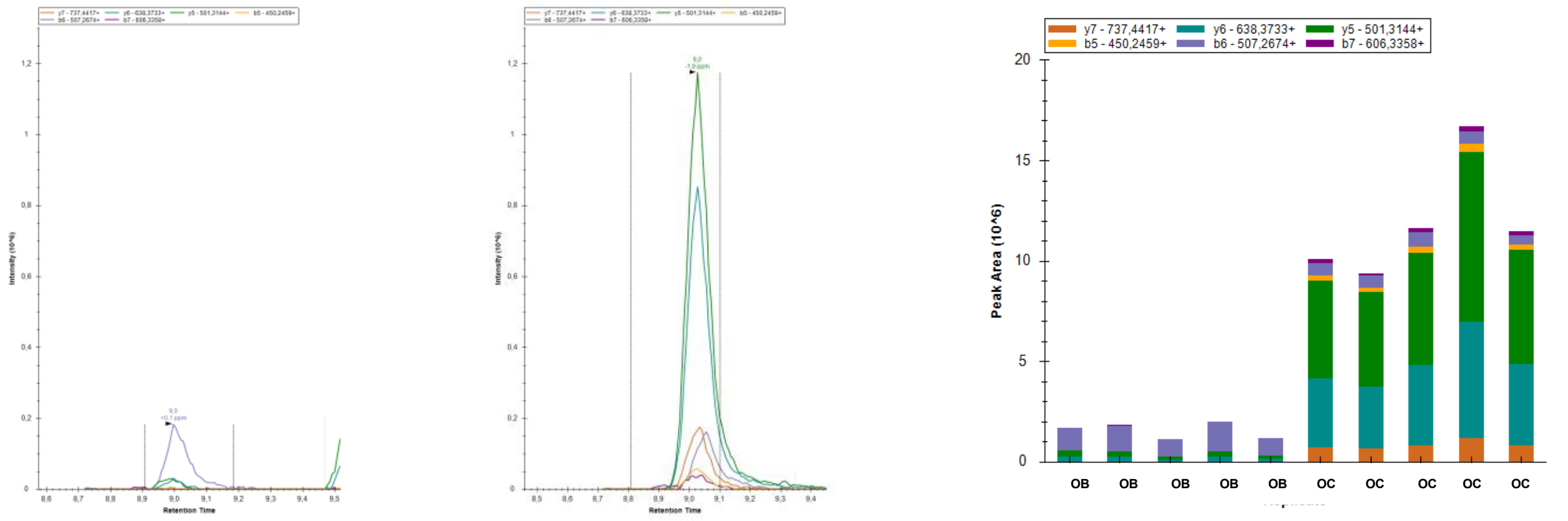

Supplementary figure S13: Representative MS2 Intensities for each peptide included in the parallel reaction monitoring (PRM) measurements for CRLD1. The header includes information on the peptide sequence, as well as peptide position within the protein, and charge state. For each peptide MS2 signals for osteoblast cells (OB, left) and osteocytes (OC, middle) are shown, as well as stacked bar charts (right) for MS2 fragment ion intensities for each replicate (n=5 per sample type).

FABPH (K.SLGVGFATR.Q [23, 31], 2+)

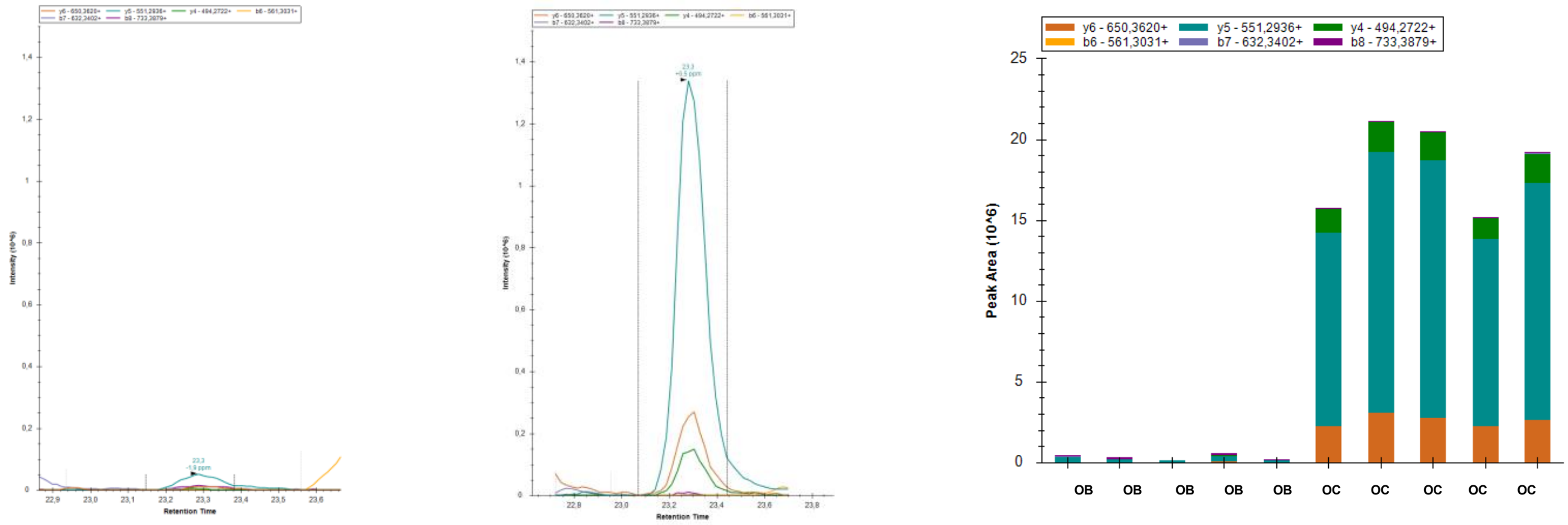

FABPH (R.QVASMTKPTTIIEK.N [32, 45], 2+)

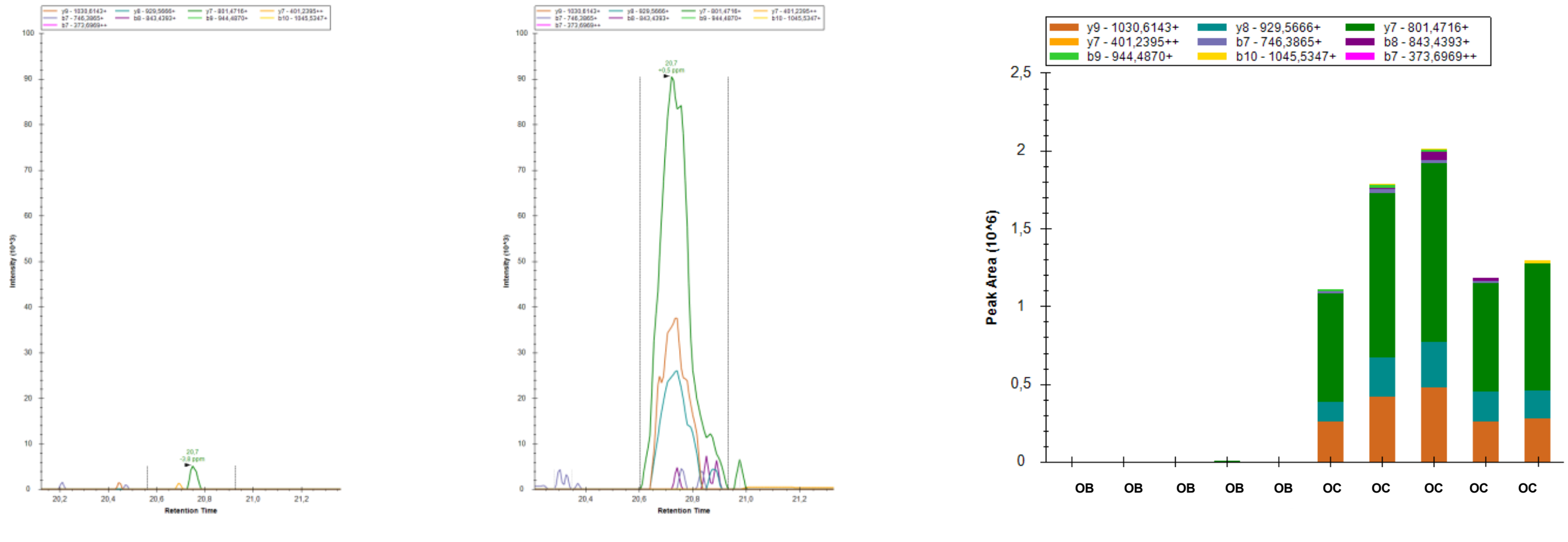

FABPH (K.NGDILTLK.T [46, 53], 2+)

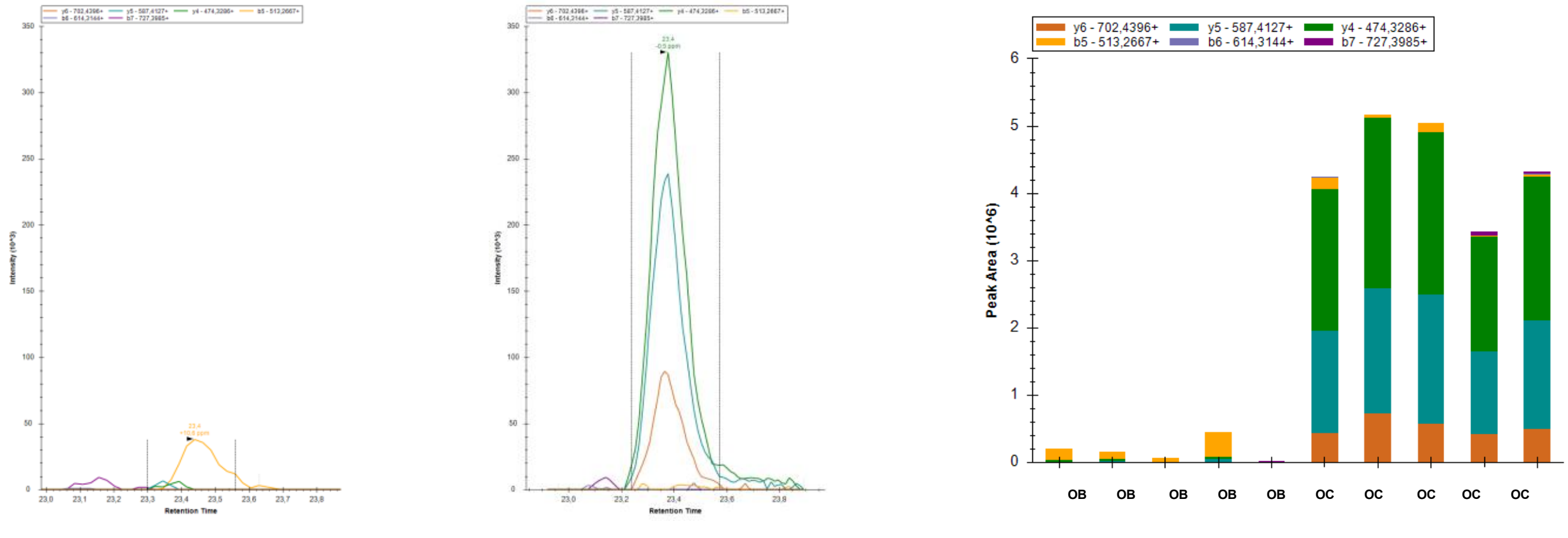

FABPH (K.SIVTLDGGK.L [83, 91], 2+)

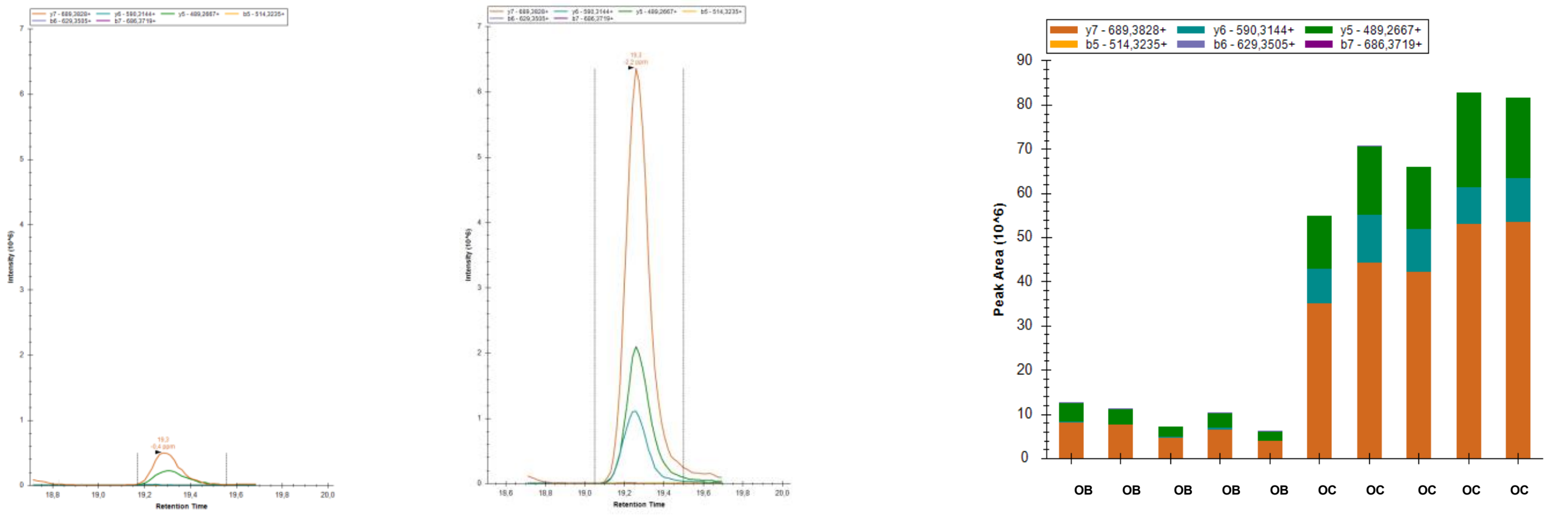

FABPH (K.WDGQETTLVR.E [98, 107], 2+)

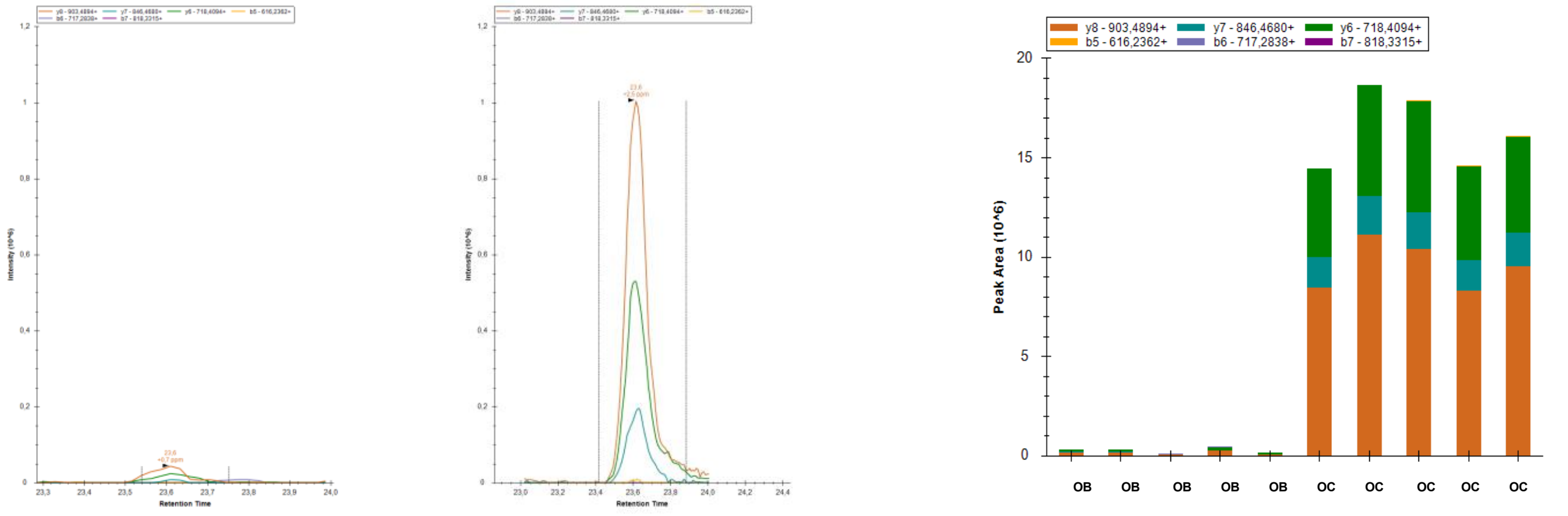

Supplementary figure S14: Representative MS2 Intensities for each peptide included in the parallel reaction monitoring (PRM) measurements for FABPH. The header includes information on the peptide sequence, as well as peptide position within the protein, and charge state. For each peptide MS2 signals for osteoblast cells (OB, left) and osteocytes (OC, middle) are shown, as well as stacked bar charts (right) for MS2 fragment ion intensities for each replicate (n=5 per sample type).

GDF15 (R.ASFPGPSELHSEDSR.F [38, 52], 3+)

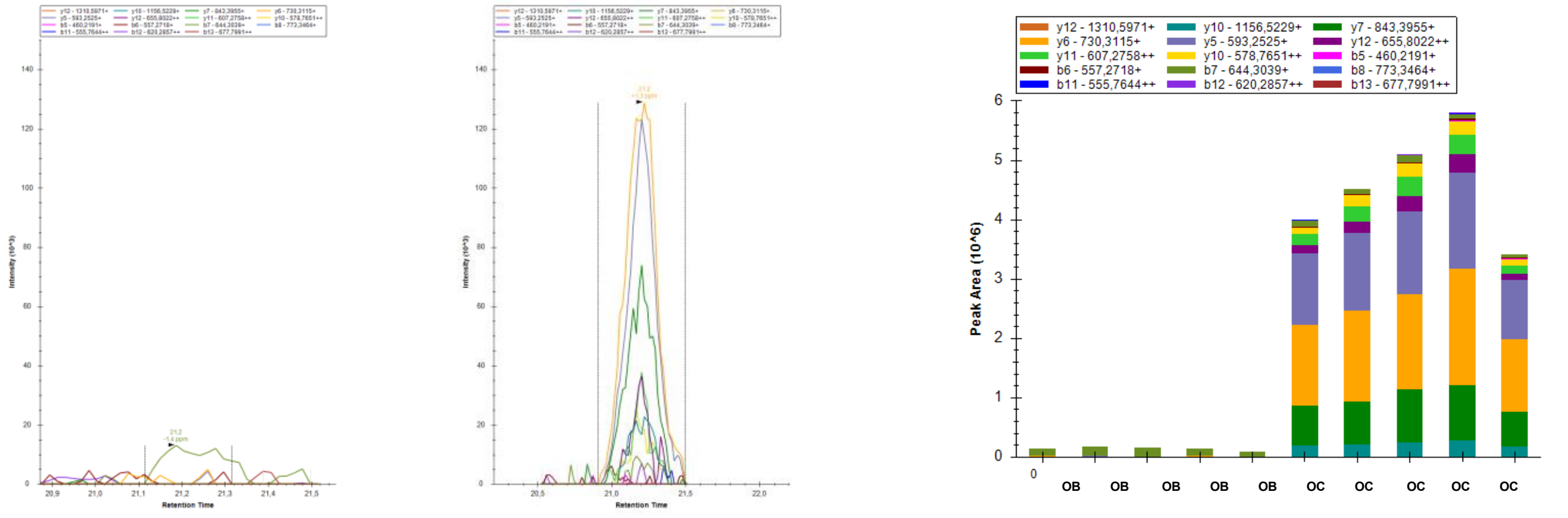

GDF15 (R.YEDLLTR.L [60, 66], 2+)

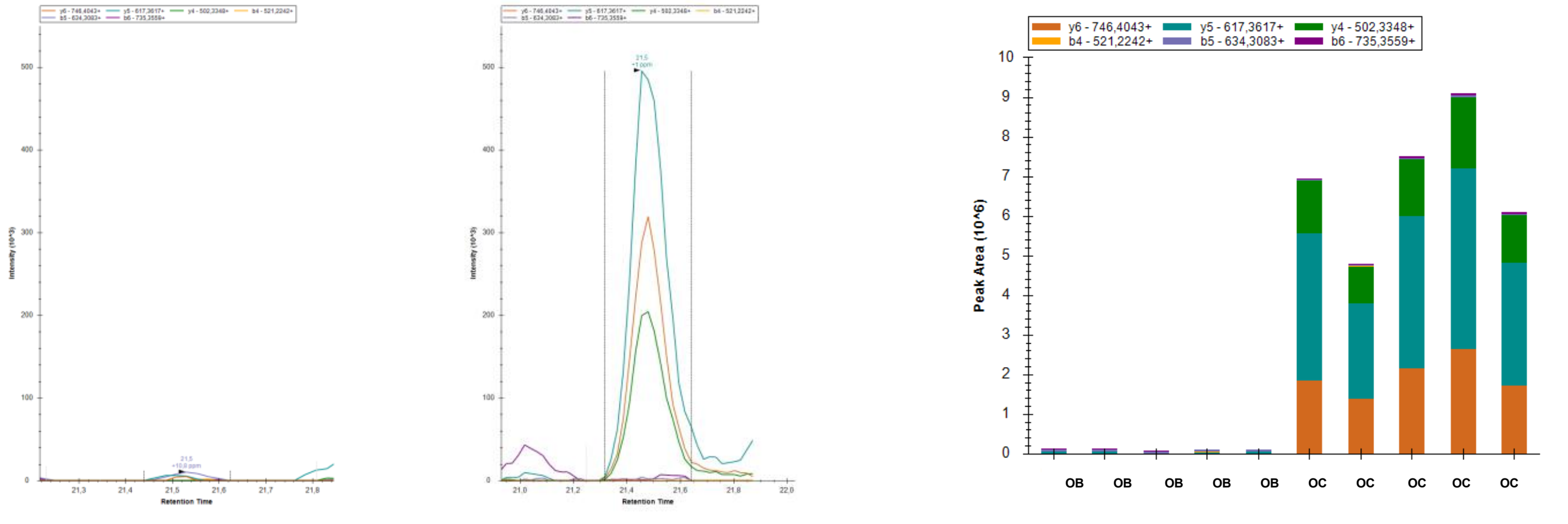

GDF15 (R.ILTPEVR.L [88, 94], 2+)

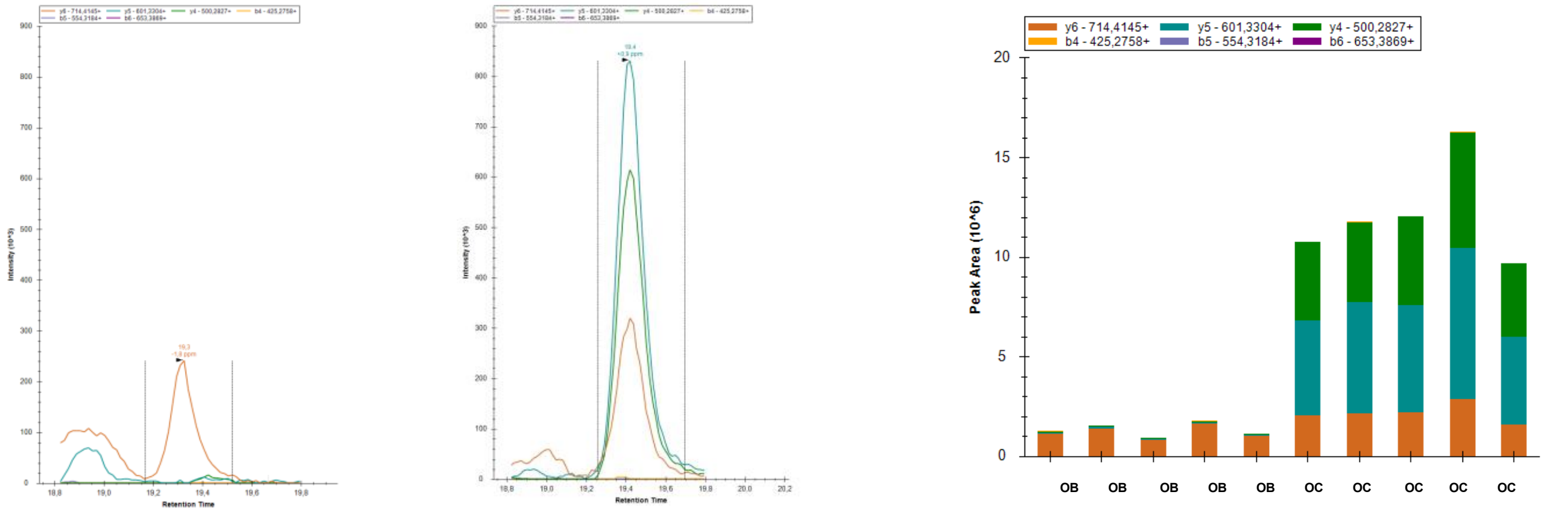

GDF15 (R.AALPEGLPEASR.L [108, 119], 2+)

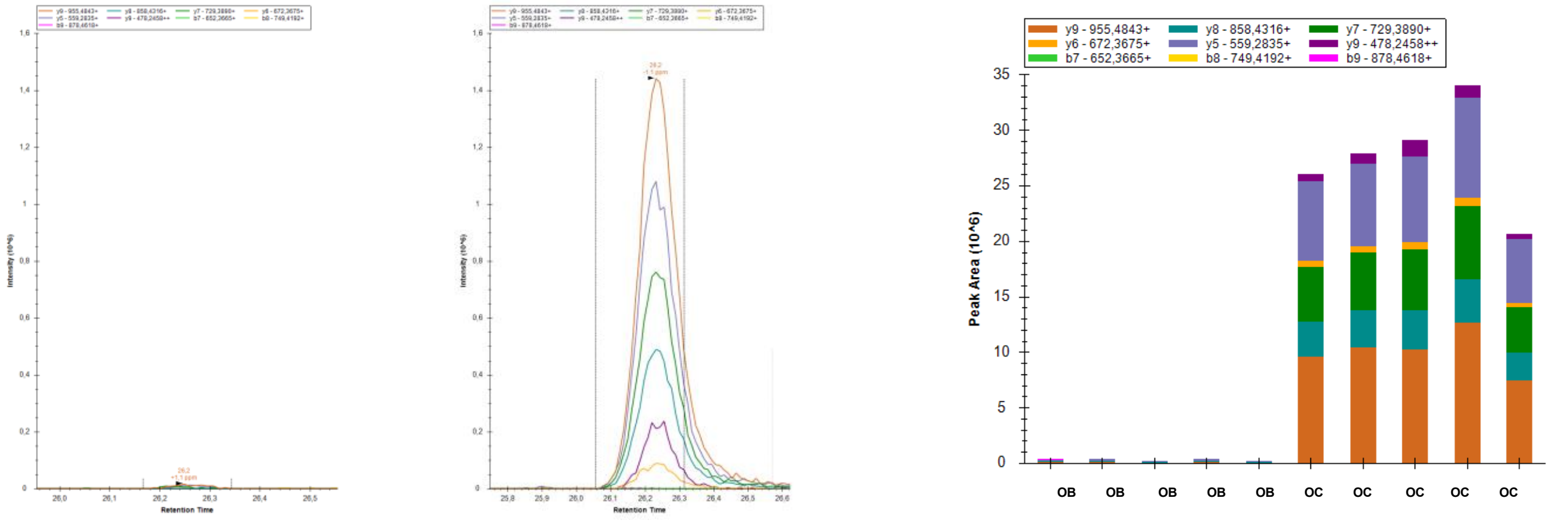

GDF15 (R.AANMHAQIK.T [250, 258], 2+)

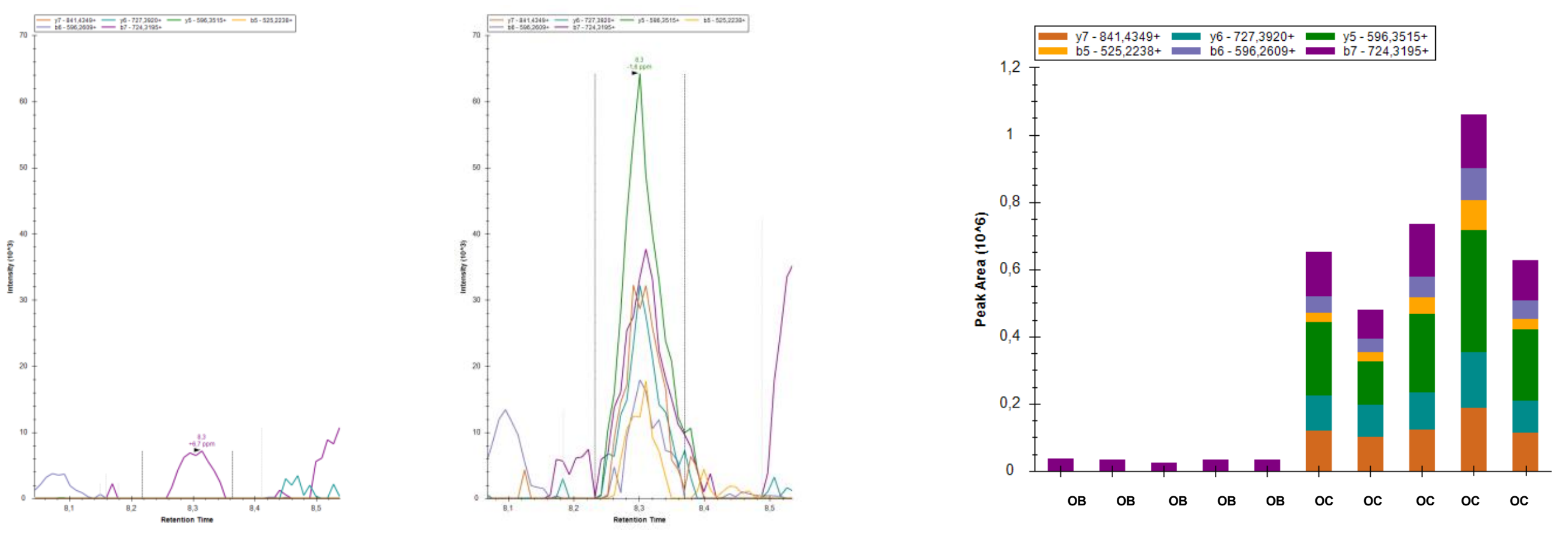

GDF15 (K.TDTGVSLQTYDDLLAK.D [288, 303], 2+)

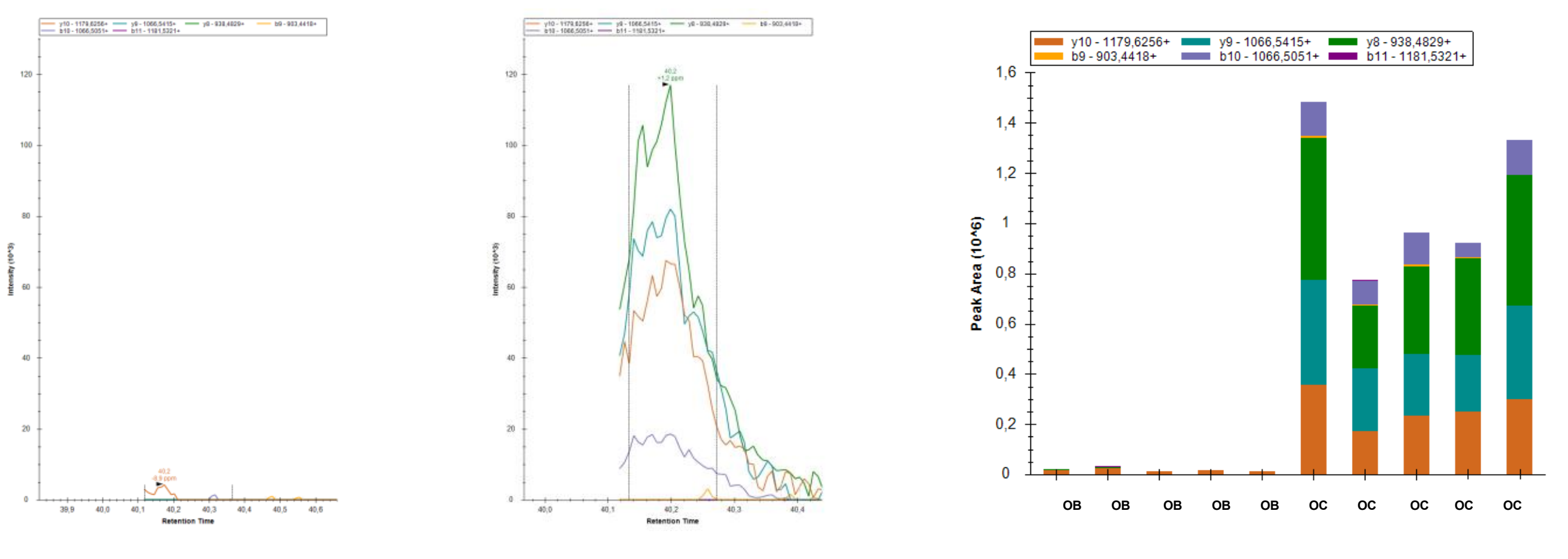

Supplementary figure S15: Representative MS2 Intensities for each peptide included in the parallel reaction monitoring (PRM) measurements for GDF15. The header includes information on the peptide sequence, as well as peptide position within the protein, and charge state. For each peptide MS2 signals for osteoblast cells (OB, left) and osteocytes (OC, middle) are shown, as well as stacked bar charts (right) for MS2 fragment ion intensities for each replicate (n=5 per sample type).

GDF6 (R.VVPHEYMLSIYR.T [92, 103], 2+)

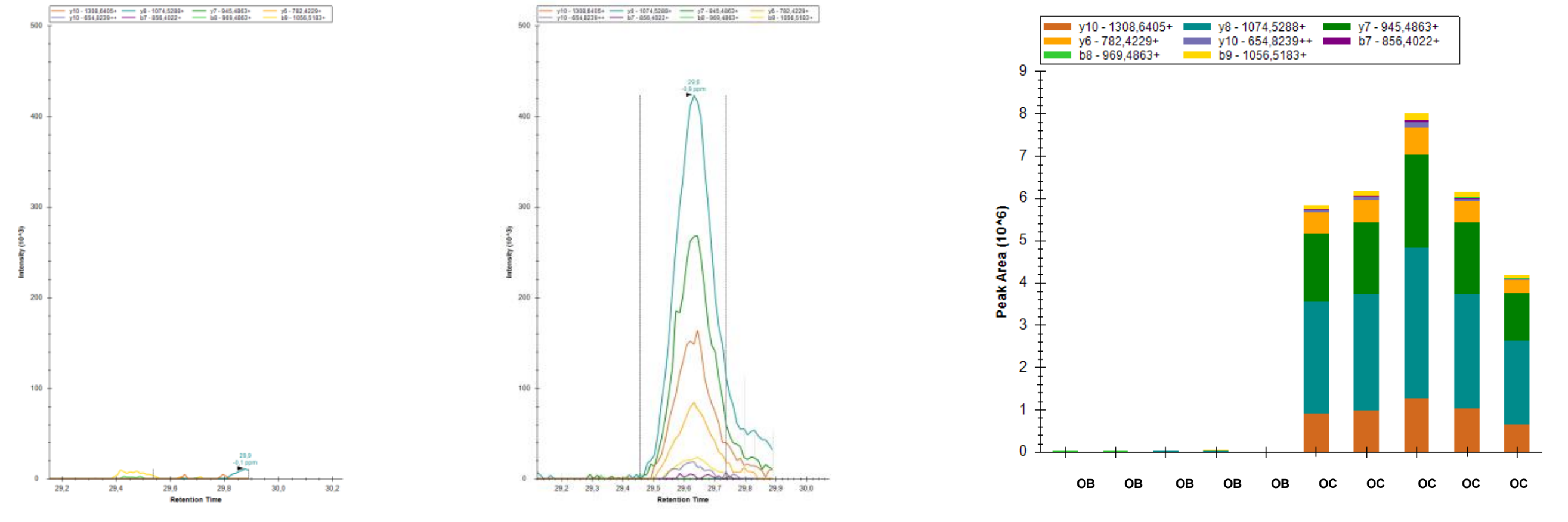

GDF6 (R.GLDDLSHTPLR.R [134, 144], 2+)

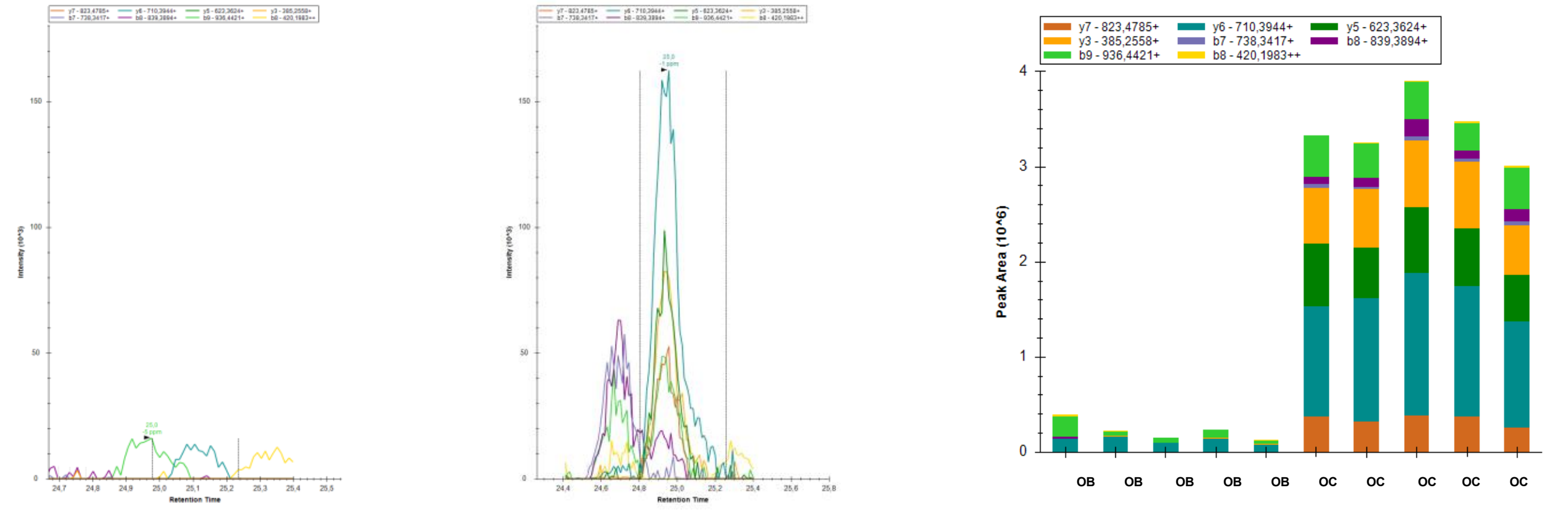

GDF6 (R.AAWGELDAGEAEAR.A [235, 248], 2+)

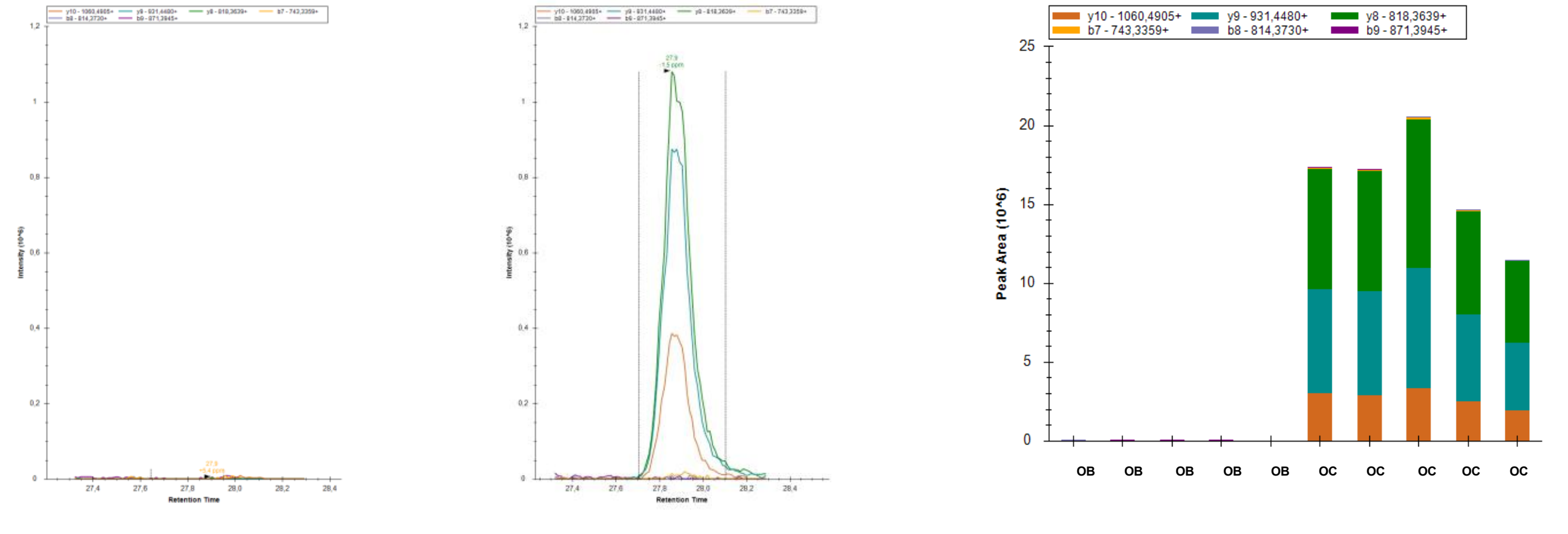

GDF6 (R.GPQQPPPPDLR.S [251, 261], 2+)

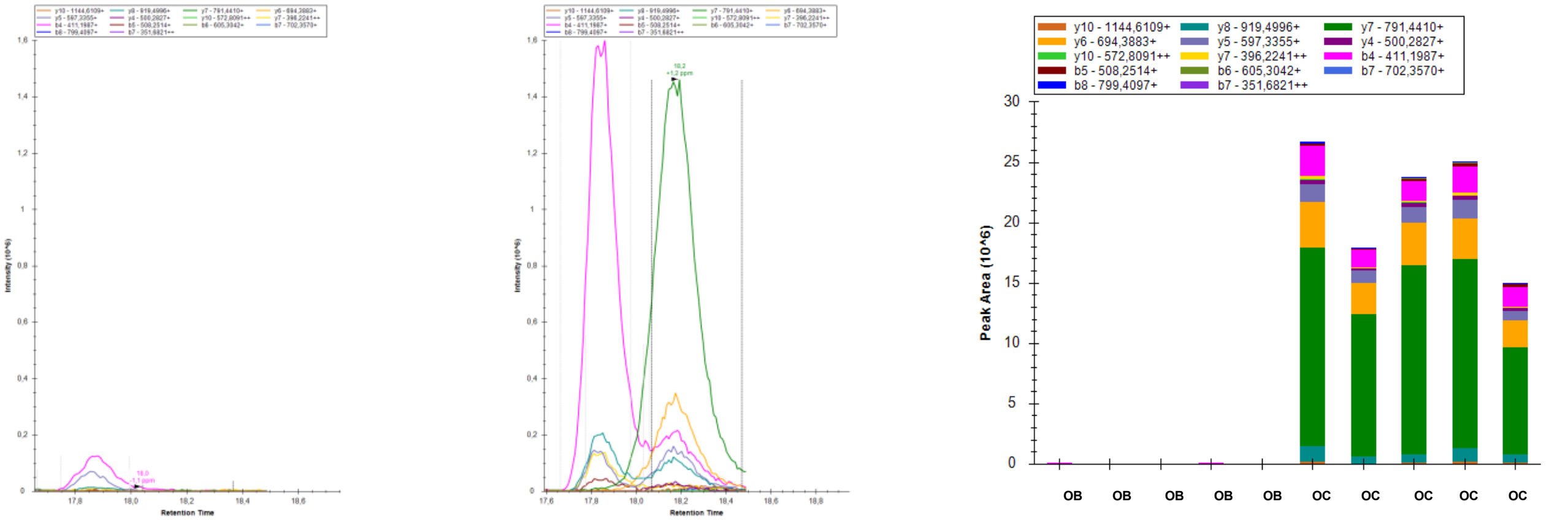

Supplementary figure S16: Representative MS2 Intensities for each peptide included in the parallel reaction monitoring (PRM) measurements for GDF6. The header includes information on the peptide sequence, as well as peptide position within the protein, and charge state. For each peptide MS2 signals for osteoblast cells (OB, left) and osteocytes (OC, middle) are shown, as well as stacked bar charts (right) for MS2 fragment ion intensities for each replicate (n=5 per sample type).

HHIP (R.QPVGALHSGDGSQR.L [228, 241], 3+)

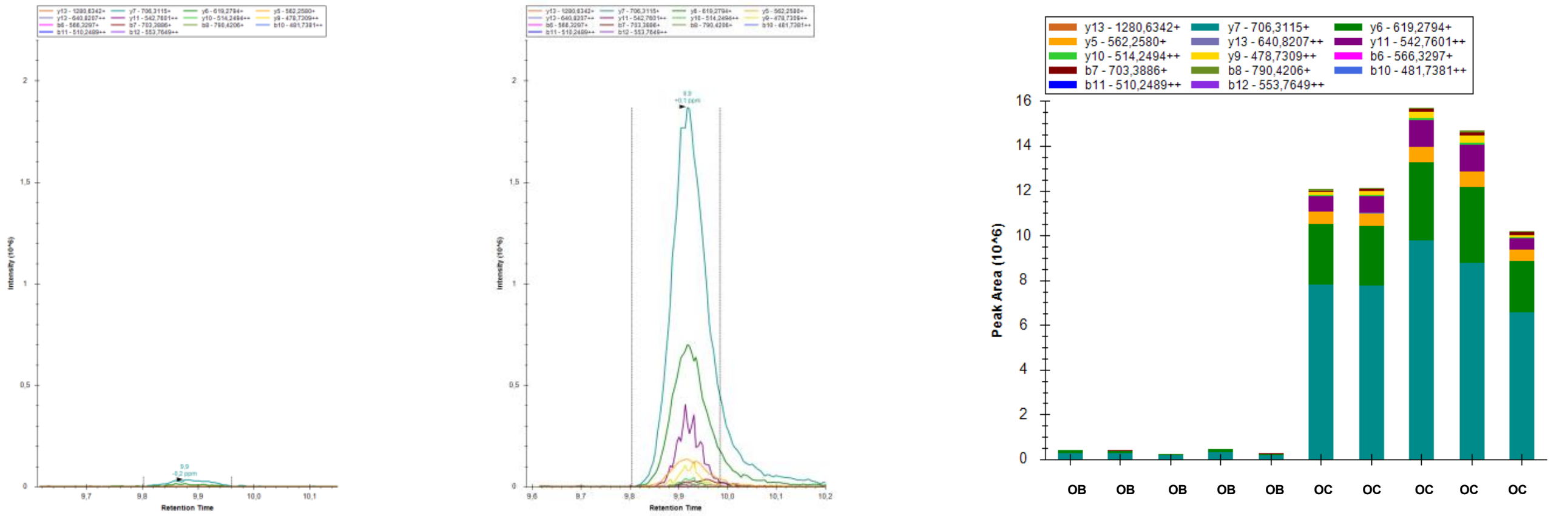

HHIP (R.GLLSLAFHPNYK.K [283, 294], 3+)

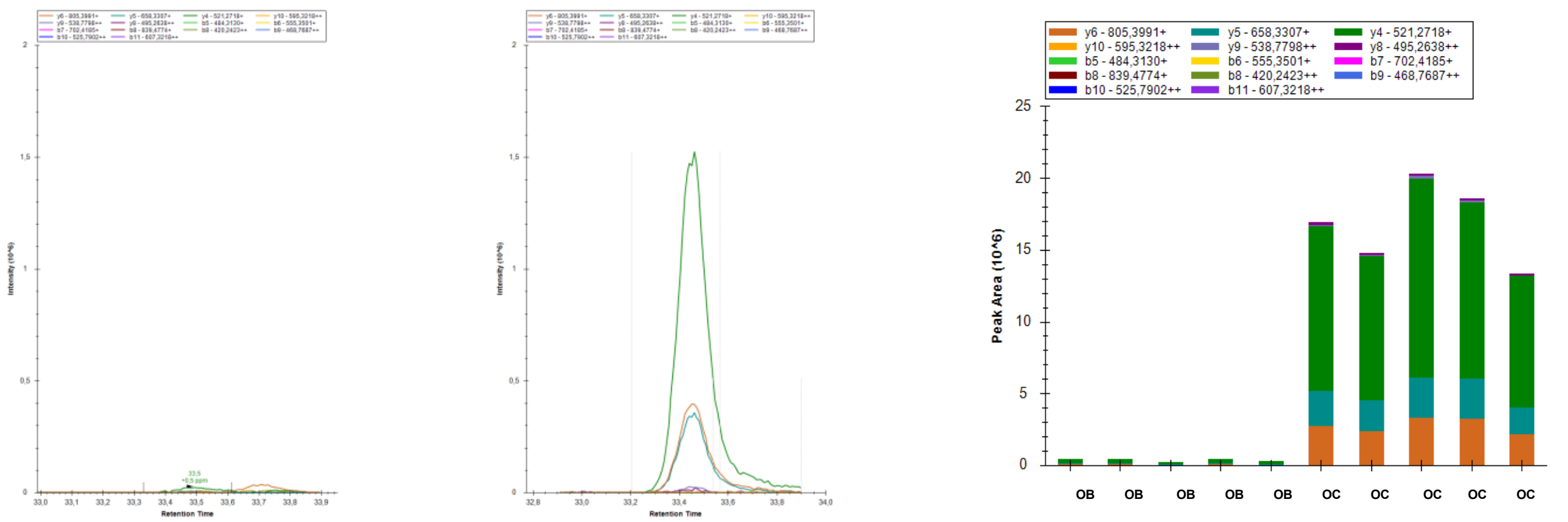

HHIP (K.LYVSYTTNQER.W [299, 309], 2+)

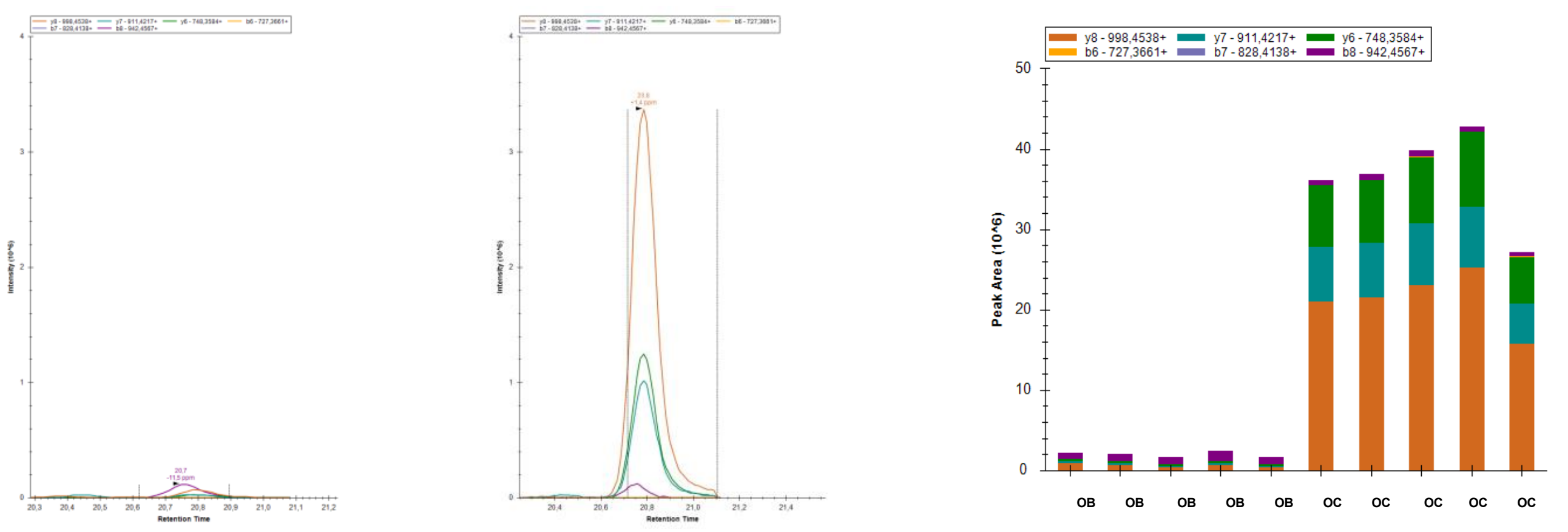

HHIP (R.VVEYTVSR.K [321, 328], 2+)

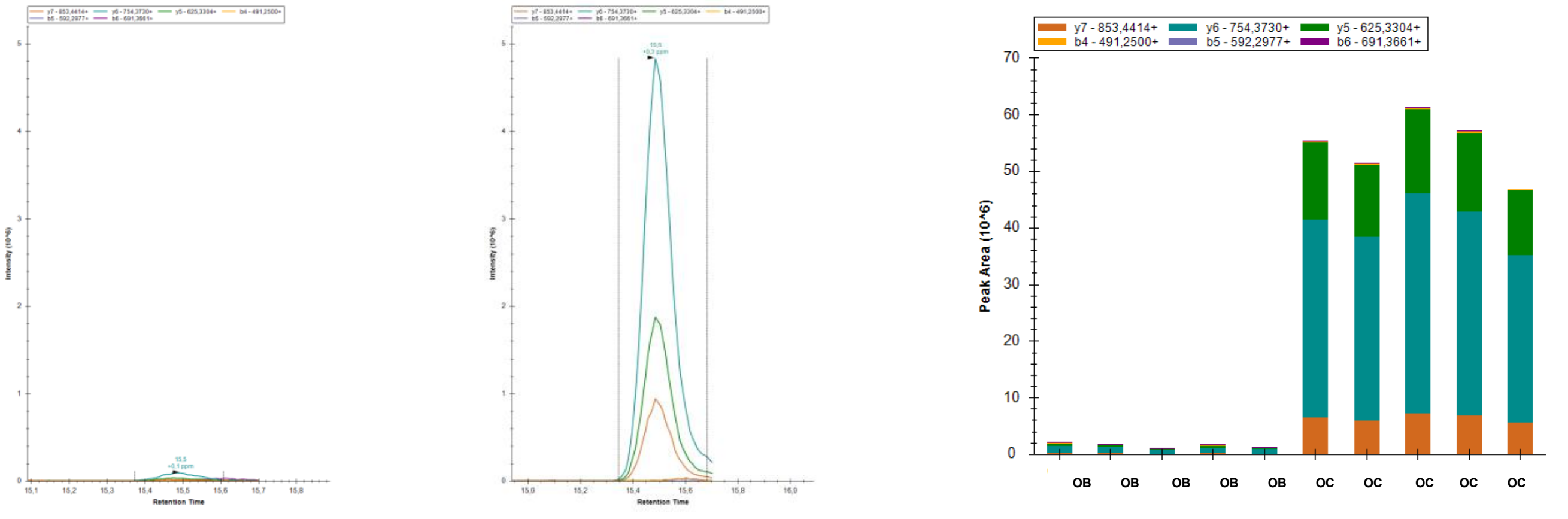

HHIP (R.NGNFLTLQQSPVTK.Q [515, 528], 2+)

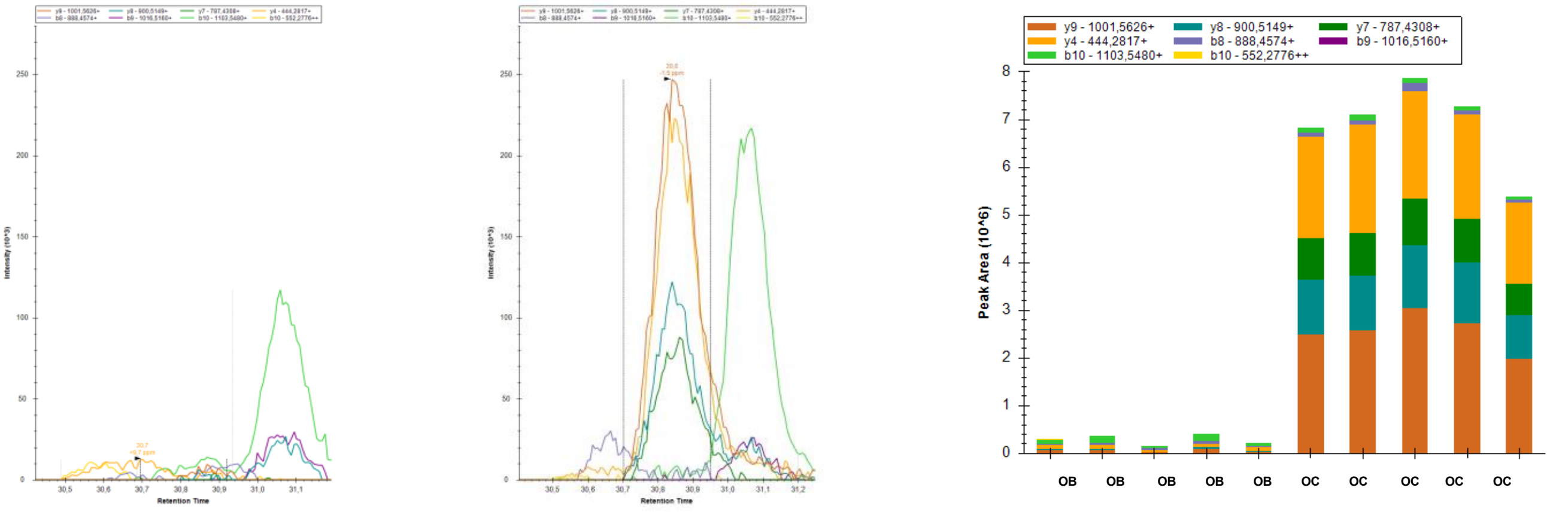

Supplementary figure S17: Representative MS2 Intensities for each peptide included in the parallel reaction monitoring (PRM) measurements for HHIP. The header includes information on the peptide sequence, as well as peptide position within the protein, and charge state. For each peptide MS2 signals for osteoblast cells (OB, left) and osteocytes (OC, middle) are shown, as well as stacked bar charts (right) for MS2 fragment ion intensities for each replicate (n=5 per sample type).

TP53I11 (K.ILGVGGEDDDGEVHR.S [27, 41], 3+)

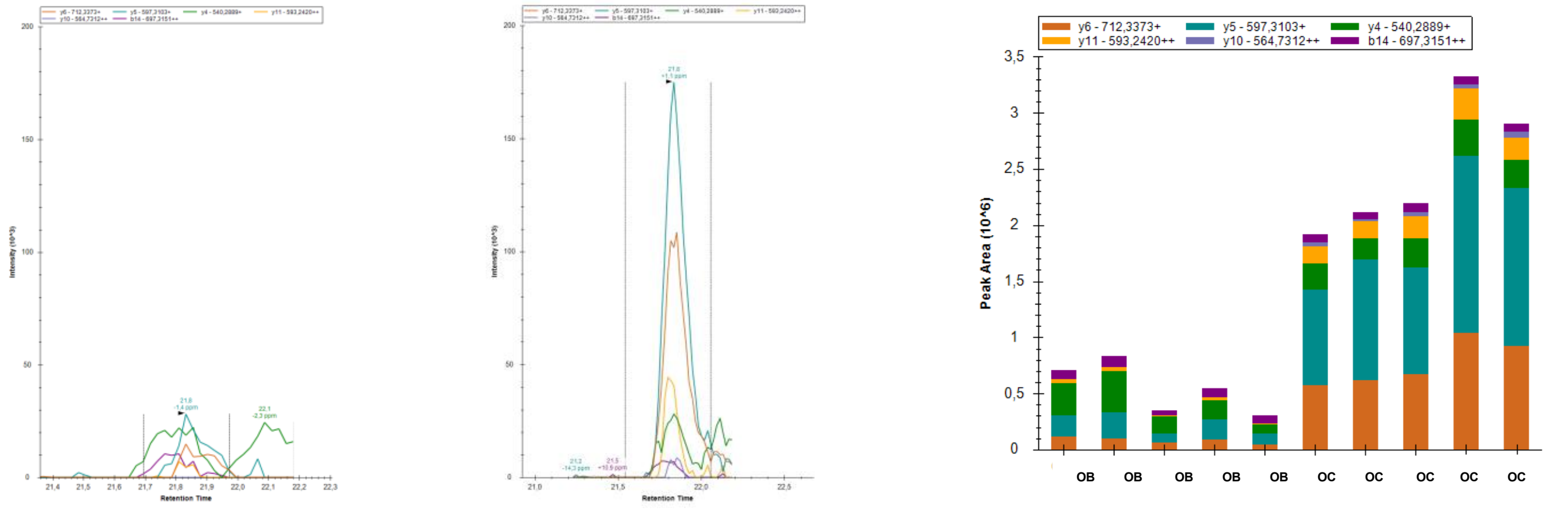

Supplementary figure S18: Representative MS2 Intensities for each peptide included in the parallel reaction monitoring (PRM) measurements for TP52I11. The header includes information on the peptide sequence, as well as peptide position within the protein, and charge state. For each peptide MS2 signals for osteoblast cells (OB, left) and osteocytes (OC, middle) are shown, as well as stacked bar charts (right) for MS2 fragment ion intensities for each replicate (n=5 per sample type).

PUSL 1 (R.LNSVEPVR.F [51, 58], 2+)

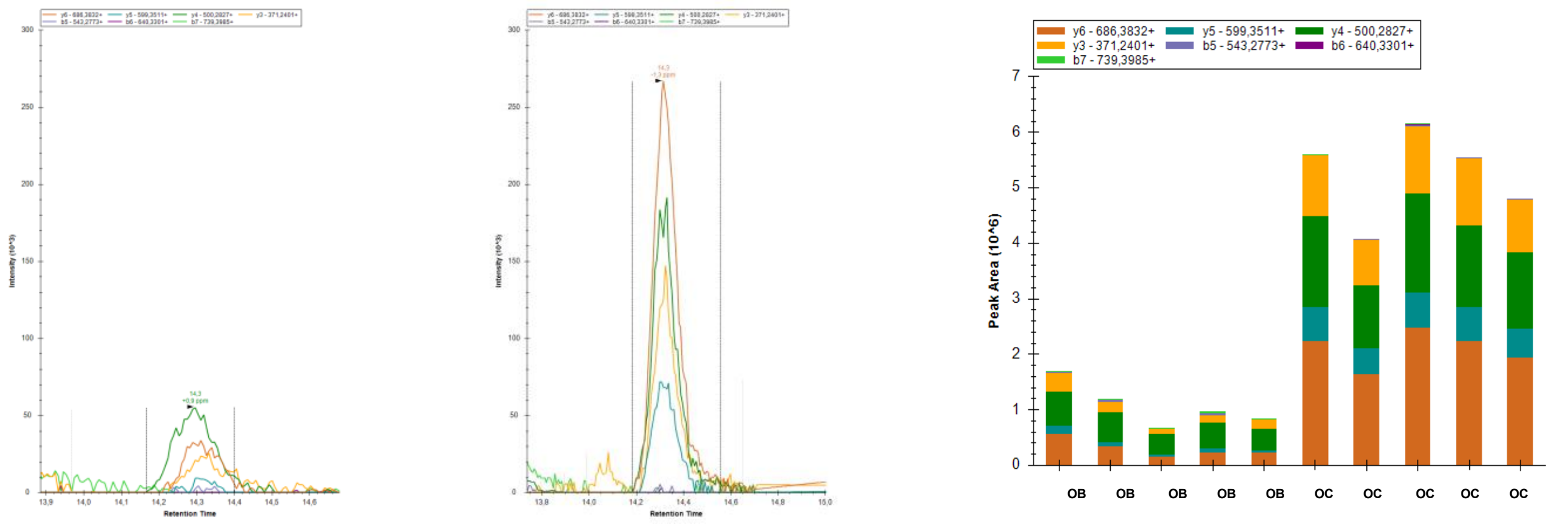

PUSL 1(R.TDAGVHALSNAAHLDVQR.R [65, 82], 3+)

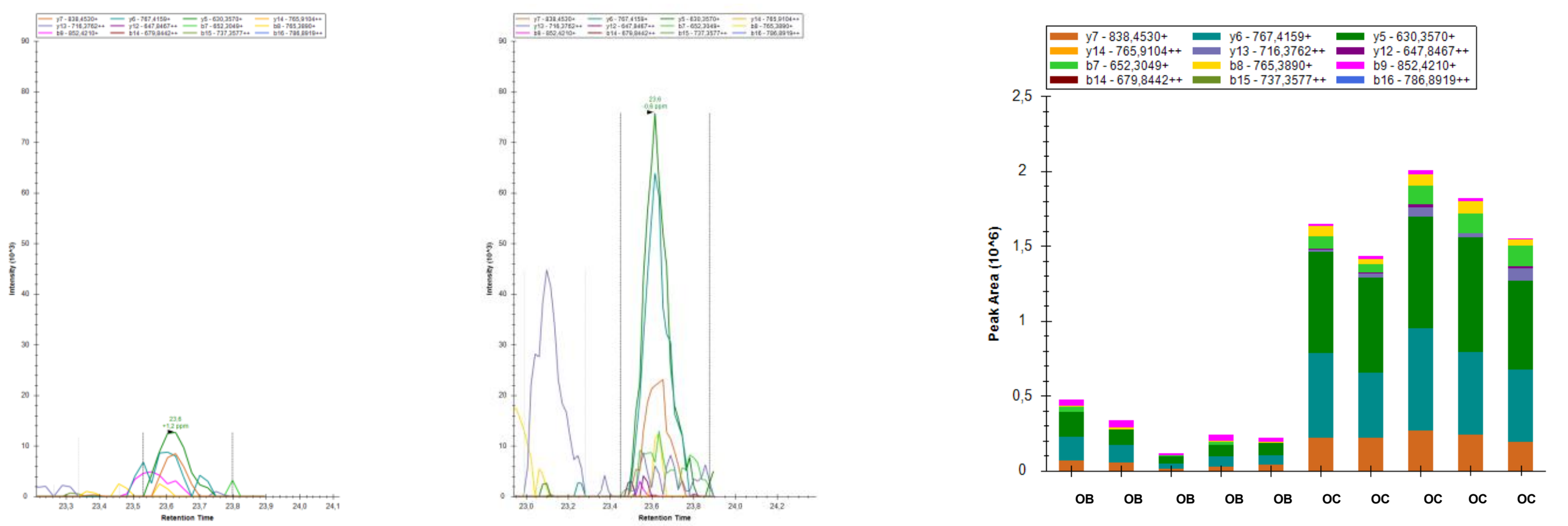

PUSL 1(R.VSVSPGQASPLVTPEESR.K [198, 215], 2+)

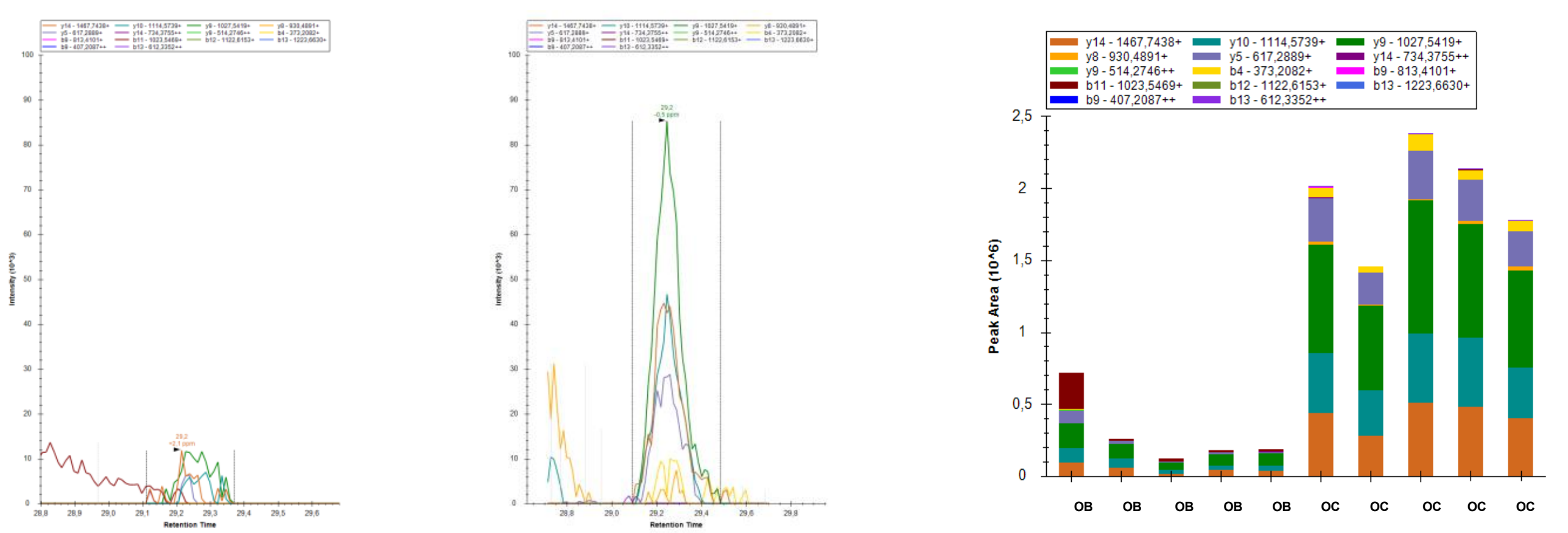

PUSL 1 (K.TILESQDPLGK.H [256, 266], 2+)

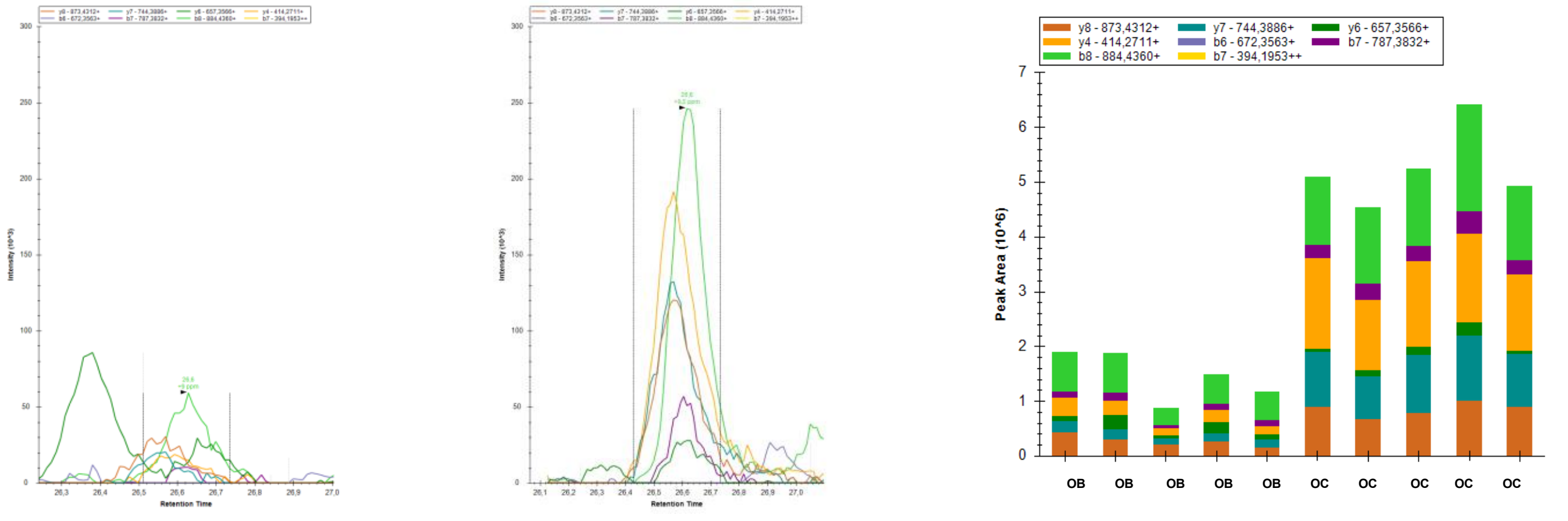

Supplementary figure S19: Representative MS2 Intensities for each peptide included in the parallel reaction monitoring (PRM) measurements for PUSL1. The header includes information on the peptide sequence, as well as peptide position within the protein, and charge state. For each peptide MS2 signals for osteoblast cells (OB, left) and osteocytes (OC, middle) are shown, as well as stacked bar charts (right) for MS2 fragment ion intensities for each replicate (n=5 per sample type).

RFT1(K.LQTLPVSR.I [213, 220], 2+)

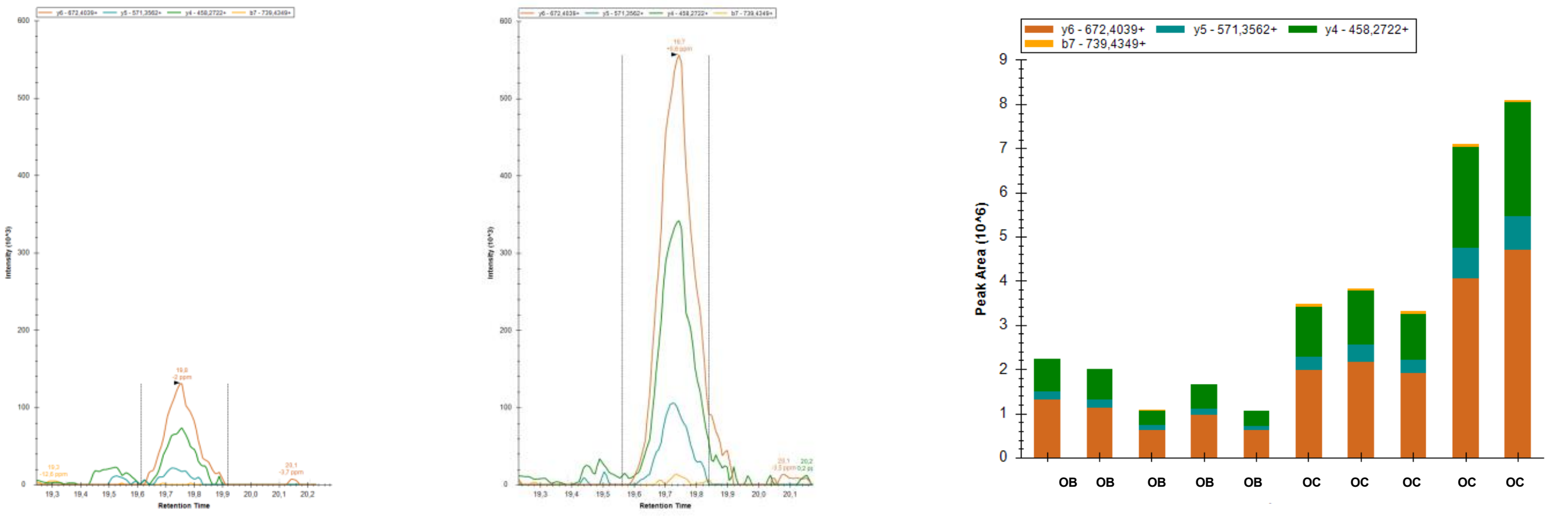

Supplementary figure S20: Representative MS2 Intensities for each peptide included in the parallel reaction monitoring (PRM) measurements for RFT1. The header includes information on the peptide sequence, as well as peptide position within the protein, and charge state. For each peptide MS2 signals for osteoblast cells (OB, left) and osteocytes (OC, middle) are shown, as well as stacked bar charts (right) for MS2 fragment ion intensities for each replicate (n=5 per sample type).

SRPX2 (K.GGNYHSSLGTR.C [76, 86], 2+)

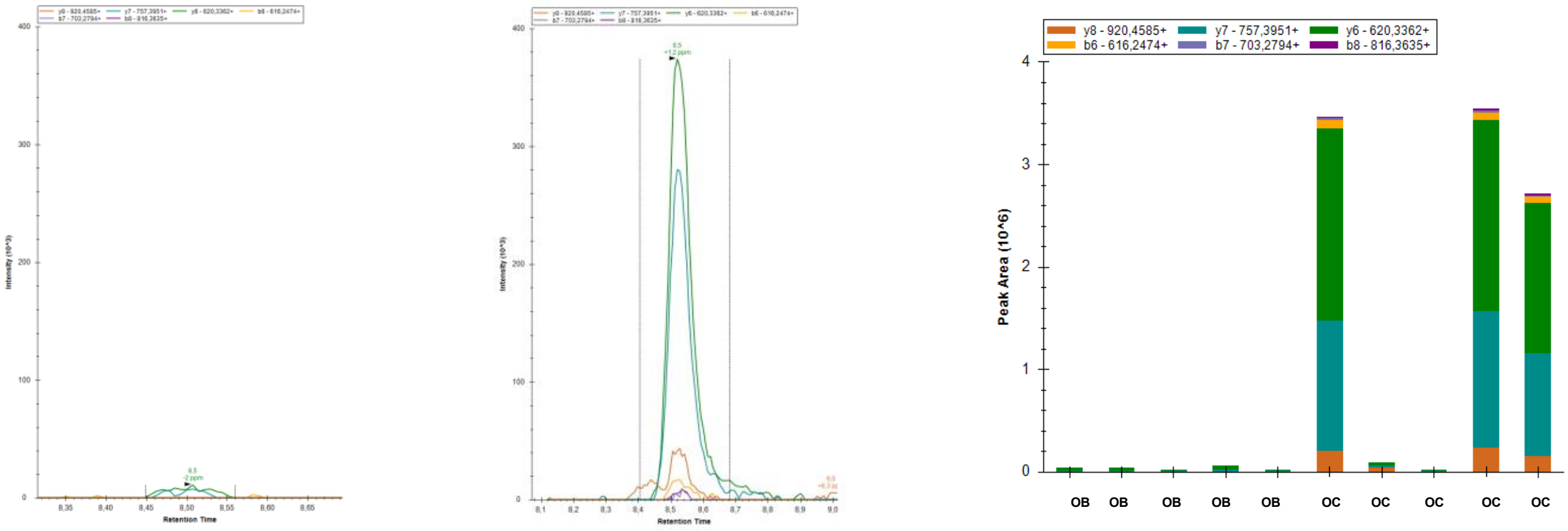

SRPX2 (R.GPEPGSHFPEGEHVIR.Y [225, 240], 2+)

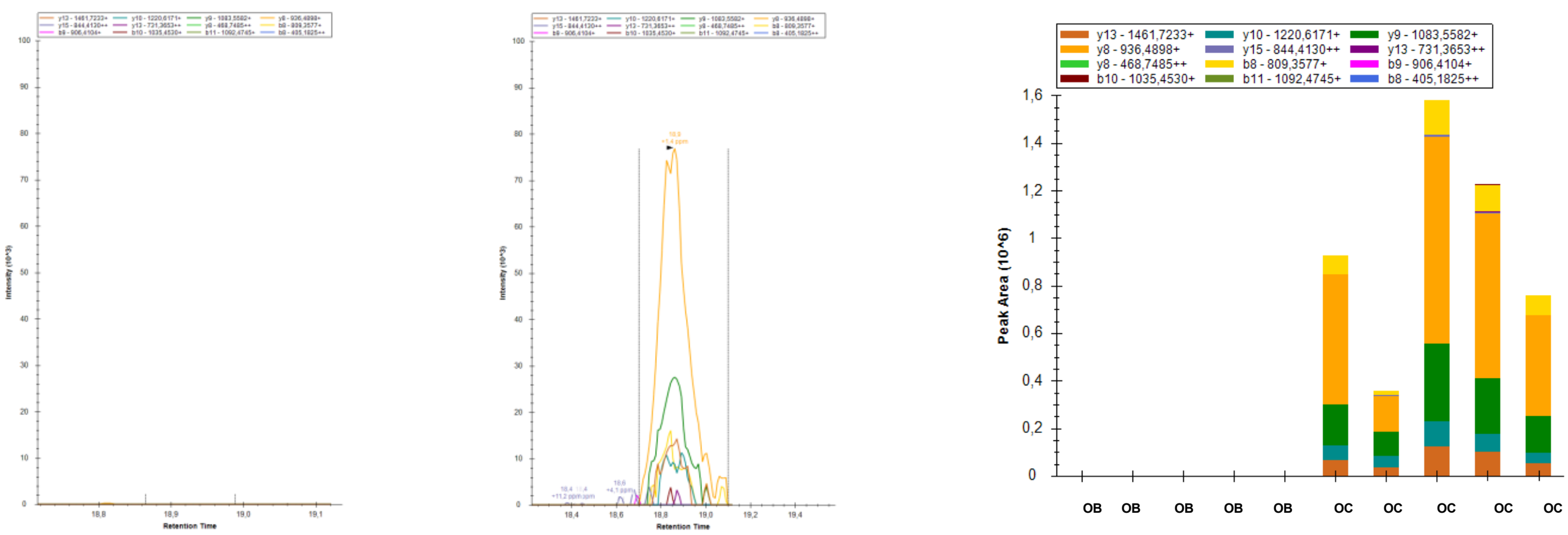

SRPX2 (R.LLIISAPDPSNR.Y [344, 355], 2+)

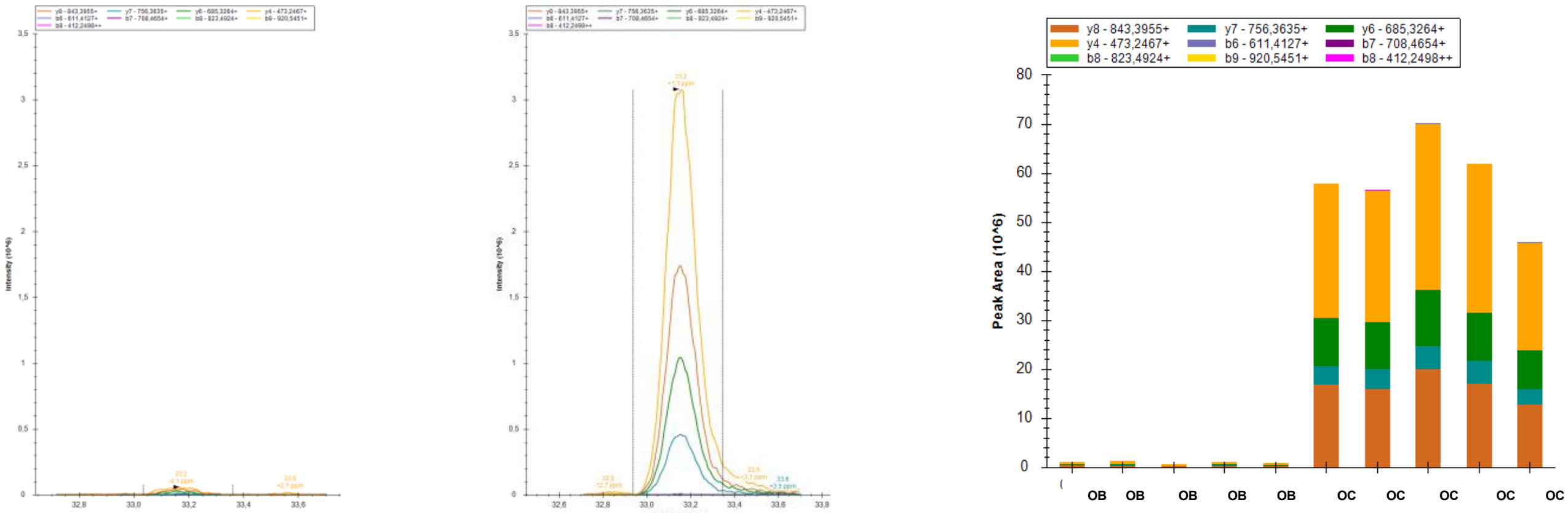

SRPX2 (R.HVTIIELVGQPPQEVGR.I [375, 391], 3+)

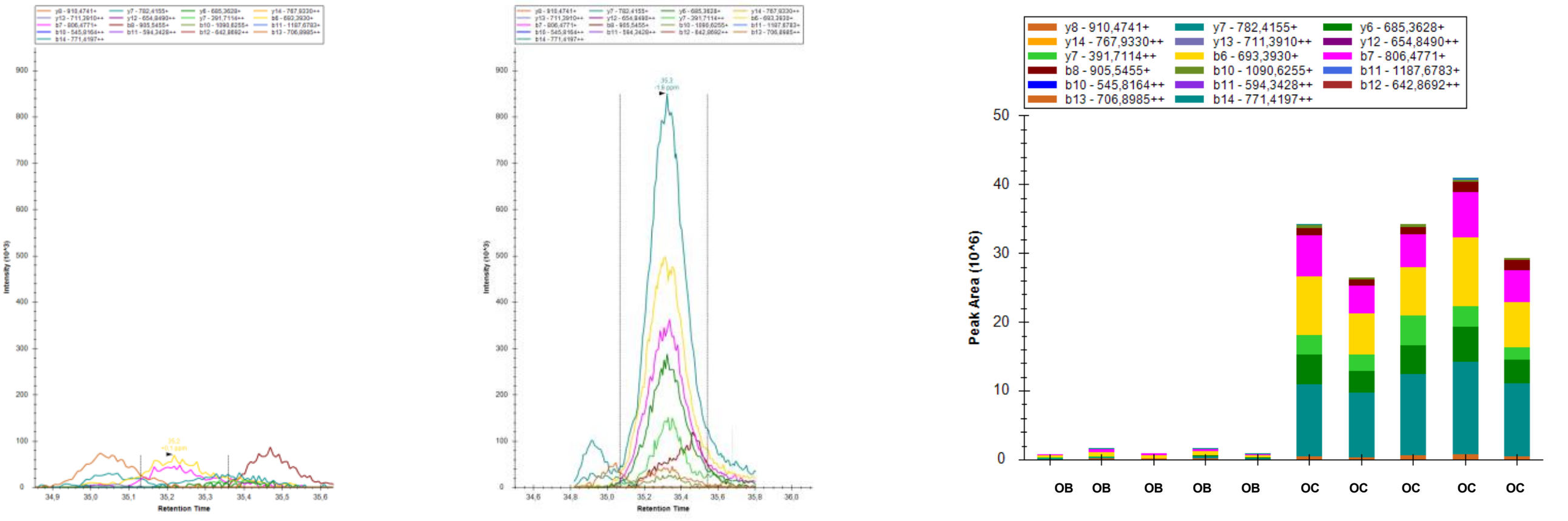

SRPX2 (R.EQQLSANIIEELR.Q [394, 406], 2+)

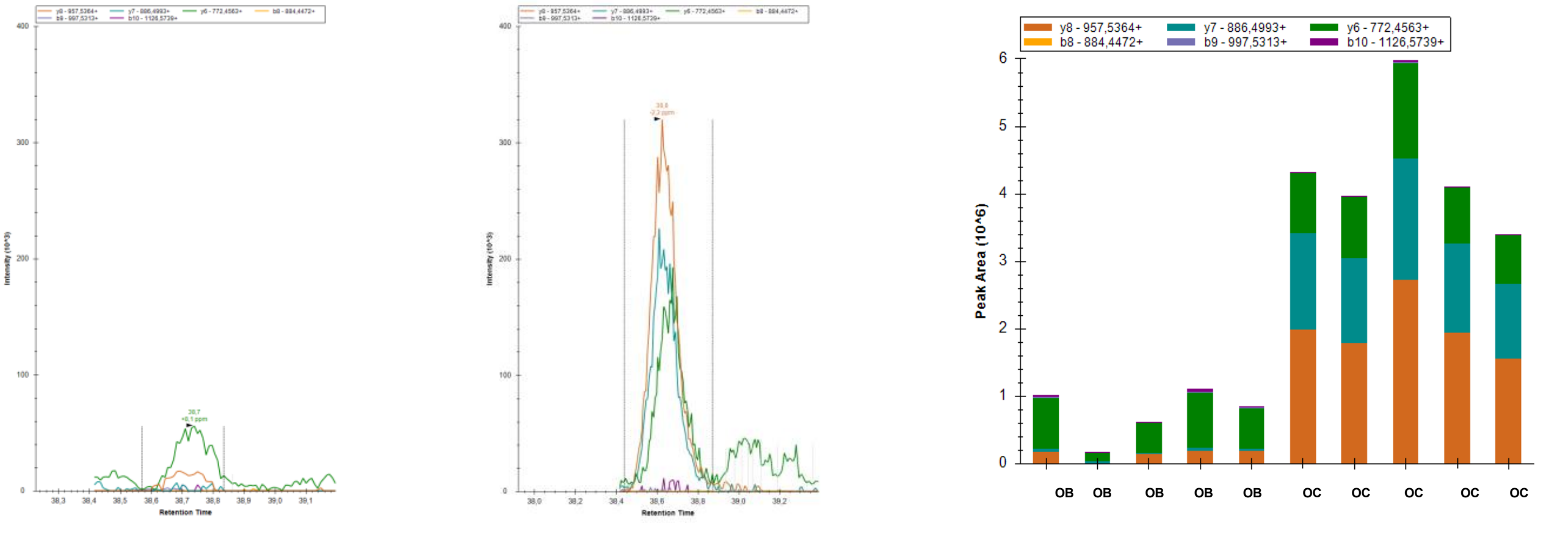

SRPX2 (R.SYFNMVLIDK.Q [414, 423] 2+)

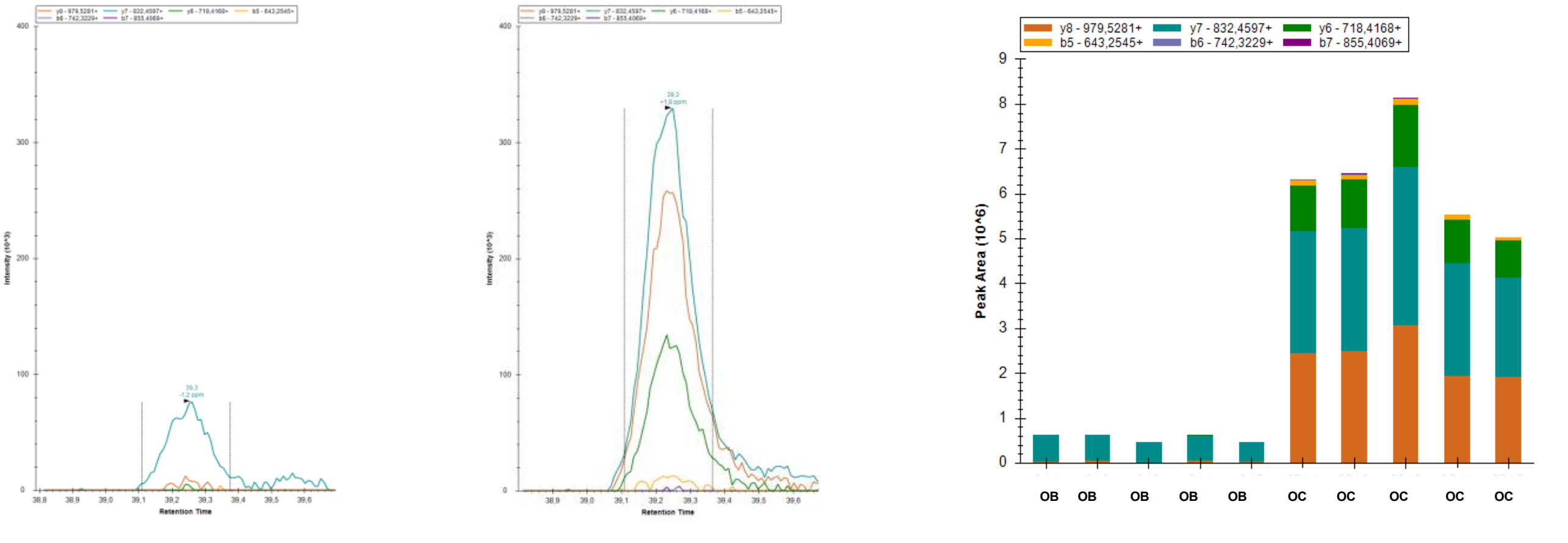

Supplementary figure S21: Representative MS2 Intensities for each peptide included in the parallel reaction monitoring (PRM) measurements for SRPX2. The header includes information on the peptide sequence, as well as peptide position within the protein, and charge state. For each peptide MS2 signals for osteoblast cells (OB, left) and osteocytes (OC, middle) are shown, as well as stacked bar charts (right) for MS2 fragment ion intensities for each replicate (n=5 per sample type).

CNN1

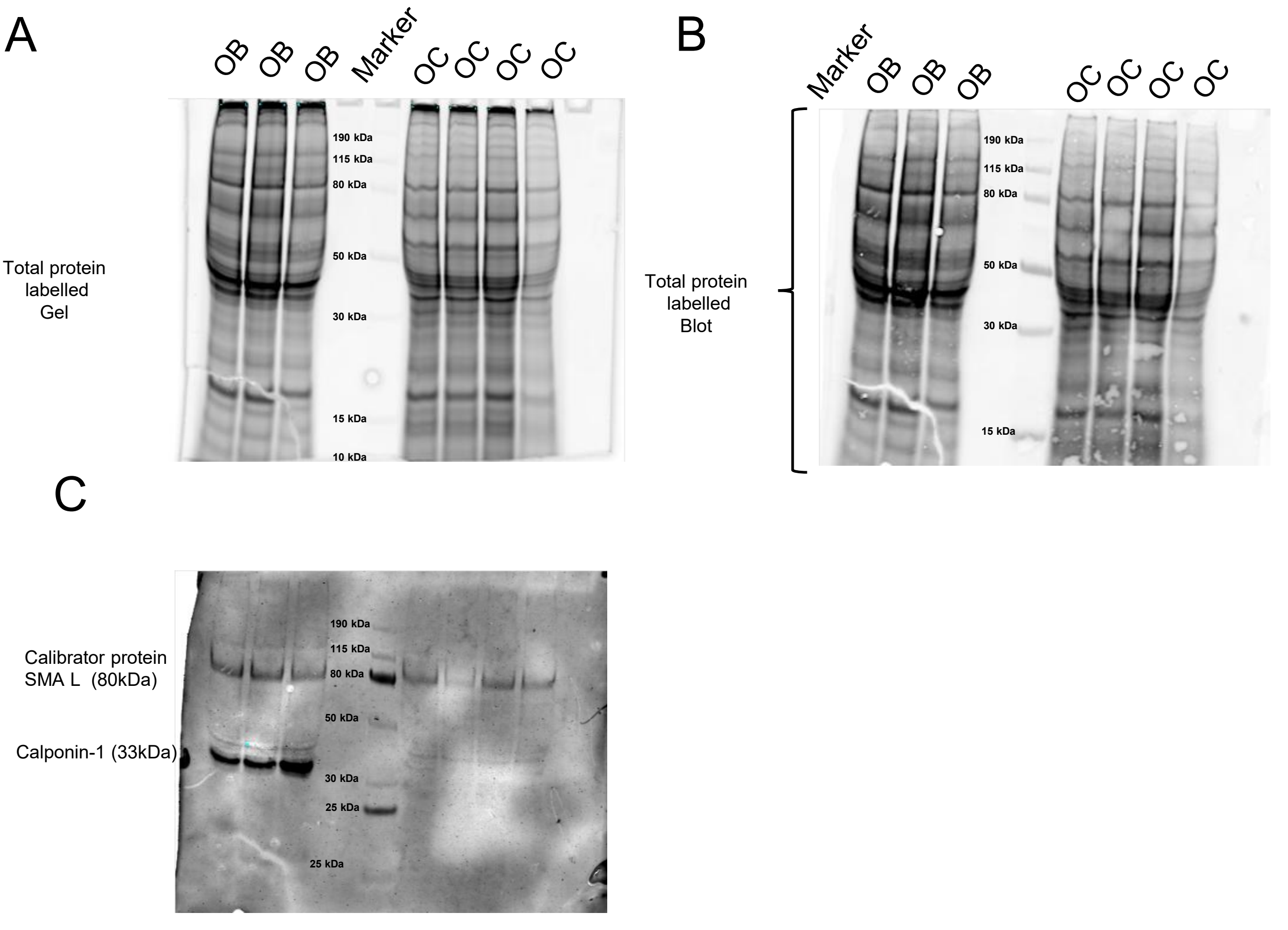

Supplementary figure S22: Quantitative Smart Protein Layers (SPL) Western Blot analysis of osteoblast (OB) and osteocyte (OC) cells,. SPL Western Blotting system (NH DyeAGNOSTICS GmbH) was used for the detection of quantitative differences of Calponin-1 (CNN1) between OB and OC cells. A) Resulting BisTris Gel and B) corresponding Blot, whereby total protein was pre-labeled with a red fluorescent fluorophore (700nm) and a 80 kDa green fluorescence-labeled standard protein (SMA L, 800 nm) was spiked in, enabling error correction of differing sample loading and data normalization between experiments. A primary antibody against CNN1 was visualized by infrared secondary antibodies (IRDye goat anti-mouse 800nm, C). It was evident that a signal for CNN1 was solely present in OB cells.

GDF15

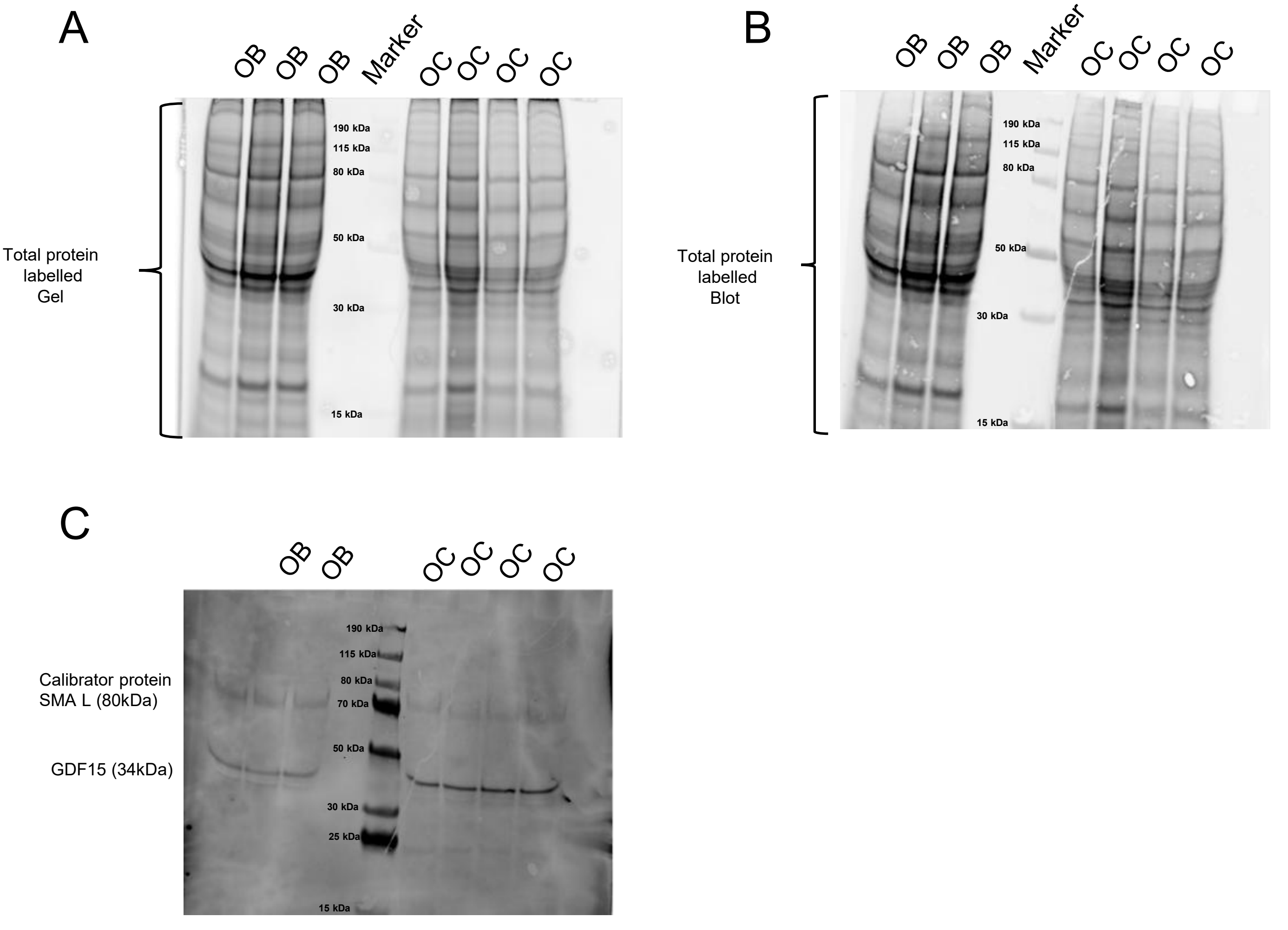

Supplementary figure S23: Quantitative Smart Protein Layers (SPL) Western Blot analysis of osteoblast (OB) and osteocyte (OC) cells,. SPL Western Blotting system (NH DyeAGNOSTICS GmbH) was used for the detection of quantitative differences of Growth differentiation factor 15 (GDF15) between OB and OC cells. A) Resulting BisTris Gel and B) corresponding Blot, whereby total protein was pre-labeled with a red fluorescent fluorophore (700nm) and a 80 kDa green fluorescence-labeled standard protein (SMA L, 800 nm) was spiked in, enabling error correction of differing sample loading and data normalization between experiments. A primary antibody against CNN1 was visualized by infrared secondary antibodies (IRDye goat anti-mouse 800nm, C). Unfortunately, signals for GDF15 were found to be present in both OB and OC cells and therefore GDF15 could not be verified as a specific OC marker.

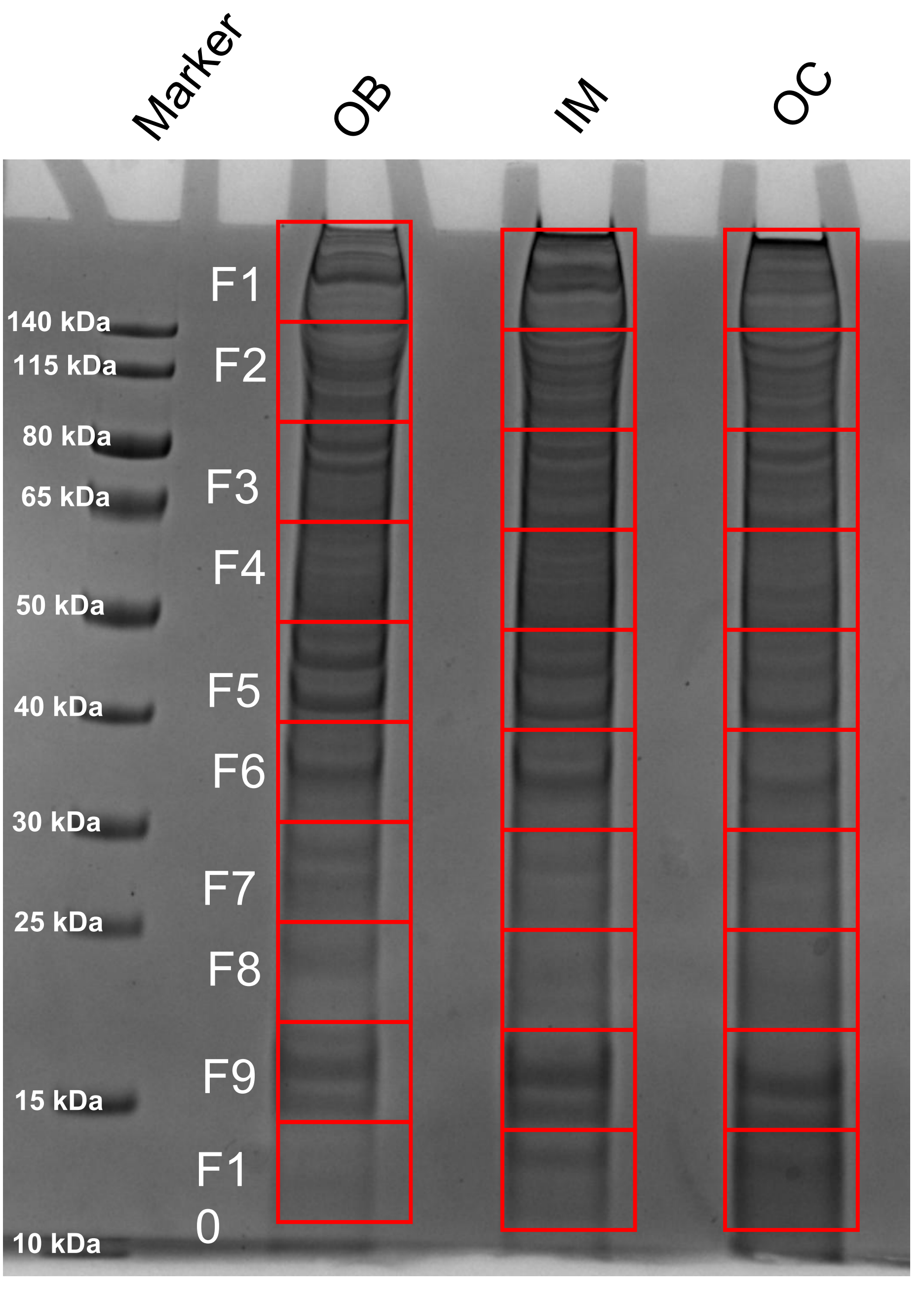

Supplementary figure S24: Gel fractionation of mastermixes of osteoblasts (OB) intermediate cell state (IM) and osteocyte (OC) samples (each n=5). A Samples were subjected to gel electrophoresis on a 12% BisTris Gel for 15min at 50V and 60min at 180V. The resulting gel was stained with Coomassie and 10 gel bands were excised for each sample. In gel digestion of proteins into peptides and mass spectrometric measurements for spectral library generation were carried out.
